# A novel algorithm for model uncertainty reduction in trapezoidal fuzzy fault tree risk assessment

**DOI:** 10.1371/journal.pone.0335759

**Published:** 2025-12-15

**Authors:** Yuanyuan Zhang, Long Zhao, Tao Zhang, Wencheng Li

**Affiliations:** 1 School of Environmental and Safety Engineering, Liaoning Petrochemical University, Fushun, Liaoning, China; 2 School of Traffic Engineering, Fujian Polytechnic of Water Conservancy and Electric Power, Yong’an, Sanming, Fujian, China; 3 CNOOC EnerTech-QHSE Services Co., Shenzhen Branch, Nanshan District, Shenzhen, China; Istanbul University: Istanbul Universitesi, TÜRKIYE

## Abstract

Intelligent risk assessment in complex systems increasingly relies on methods like trapezoidal fuzzy fault trees. However, conventional techniques often struggle with accurately calculating top-event probabilities and handling model uncertainty, which undermines the reliability of risk evaluations. To address this, we propose a novel algorithm that effectively reduces model uncertainty in trapezoidal fuzzy fault tree analysis. By leveraging the cut-set theorem and operating directly on trapezoidal fuzzy numbers without defuzzification, our approach preserves the completeness of fuzzy information throughout the calculation. The algorithm accommodates three common fault tree logics: OR-only, AND-only, and mixed OR/AND gates. In a case study on liquid ammonia leakage, the method achieved a 45.25% reduction in model uncertainty—surpassing existing approaches, which reached only 36.65%. Results showed 99.40% consistency with benchmark literature, affirming the algorithm’s accuracy. Additional analysis using OREDA field data demonstrated the method’s stability across different system structures and risk levels. To further verify robustness under input uncertainty, we conducted 60 perturbation tests on representative basic events with high, medium, and low failure probabilities. The algorithm exhibited exceptional stability, with output variations remaining below ±1% even under ±15% input perturbations, confirming its suitability for real-world applications where data uncertainties are common. This work offers a reliable and scalable solution for risk assessment in high-stakes industries such as nuclear energy and chemical processing.

## Introduction

Intelligent risk assessment plays a crucial role in the era of big data and machine learning [1–3]. Among the various techniques, trapezoidal fuzzy fault tree analysis has emerged as a powerful tool by integrating traditional fault tree logic with fuzzy set theory to handle system uncertainties [4,5]. However, significant challenges persist in its application, particularly concerning model uncertainties that can undermine the reliability of assessment results [6,7].

Uncertainties in trapezoidal fuzzy fault tree assessment generally fall into three categories: data uncertainty [8–10], stemming from insufficient data and limited expert knowledge; relational uncertainty [11,12], arising from complex dependencies among basic events; and model uncertainty [13], resulting from computational approximations that may introduce information distortion. These uncertainties propagate upward through the fault tree hierarchy, from basic events to the top event, ultimately compromising the accuracy of risk assessment outcomes [14,15].

To mitigate data uncertainty, researchers have developed a range of methods centered on extending fuzzy mathematics, integrating expert knowledge, and constructing hybrid models. For instance, Liu et al. [16] incorporated intuitionistic trapezoidal fuzzy numbers into fault tree analysis and introduced a new ranking method based on expected values and compromise possibilities. Although this approach helped quantify the membership and non-membership degrees of basic event probabilities, it did not fully address multi-level uncertainty propagation. Kaushik and Kumar [17] later developed intuitionistic fuzzy importance measures using α-cut operations to process qualitative linguistic data, improving the reliability of criticality analysis, though computational complexity limited its use in large-scale systems. Yu et al. [18] applied the weakest t-norm to trapezoidal fuzzy number aggregation, reducing distortion and bringing results closer to statistical values compared to conventional linear weighting. Komal [19] extended this idea by combining Tω operators with fuzzy reliability theory in a generalized lambda-tau methodology for repairable systems, though its adaptability in dynamic environments remains to be fully tested. Badida et al. [20] applied a fuzzy fault tree framework to pipeline failure estimation, enabling risk assessment without historical data via Fussel-Vesely importance measures, albeit with reliance on subjective expert weighting. To address this, Lu et al. [21] introduced an improved SAM method using a butterfly optimization algorithm to adaptively determine the relaxation factor β, removing the need for manual tuning. Zhang et al. [22] combined regret theory with squared confidence Pythagorean fuzzy hybrid weighted operators in a multi-criteria decision model, improving semantic interpretability and reliability in information aggregation. Sun et al. [23] further proposed a Pythagorean fuzzy Bayesian network model using enhanced trapezoidal fuzzy Einstein geometric operators to integrate subjective and objective weights, effectively reducing prior probability uncertainty in submarine pipeline risk assessment. Kaushik and Kumar [24] built a hybrid intuitionistic fuzzy fault tree-Bayesian network model, using conditional probability tables to quantify system failure risks under imprecise data conditions. Finally, Tan et al. [25] integrated fuzzy fault trees with probabilistic risk assessment into a dynamic framework for battery energy storage safety, allowing real-time tracking of risk variations due to design parameter changes.

To address relational uncertainty, several integrated approaches have been proposed. Ding et al. [26] combined Bayesian networks with trapezoidal fuzzy fault trees to develop a dynamic risk assessment framework for residual heat removal systems, modifying conditional probability tables to represent event polymorphism and non-deterministic logic. Separately, Bi et al. [27] used particle swarm optimization to tune the parameters of a backpropagation neural network coupled with trapezoidal fault trees, capturing nonlinear data relationships and achieving high-accuracy risk prediction with a maximum error of 0.0006. Similarly, Sakar and Sokukcu [28] integrated fault trees with Bayesian networks to model conditional dependencies in pilot transfer risks, quantifying interactions between equipment failures and human operations through conditional probability tables. While effective, these methods mainly addressed static uncertainties. To overcome this, Meng et al. [29] introduced a hybrid event tree–fault tree and dynamic Bayesian network model capable of capturing interdependencies among risk factors while accounting for both stochastic and fuzzy uncertainties. Extending this effort, Ahmed et al. [30] developed a type-2 fuzzy Bayesian method to adaptively update linguistic variables in dynamic environments. Singh et al. [31] employed a hybrid model with bidirectional probabilistic updating to reveal nonlinear propagation patterns of fuzzy uncertainties in dependency networks. In a related innovation, Djemai et al. [32] converted Bowtie models into dynamic Bayesian networks, creating a bridge between system prediction and conditional dependency analysis.

To further address these challenges, particularly in integrating multi-source information and quantifying complex human factors, hybrid fault tree models have been increasingly adopted. For example, Saraliolu et al. [33] integrated the Human Factors Analysis and Classification System (HFACS) with fuzzy FTA to analyze ship engine room fires. Their model uses HFACS to structurally classify human factors and employs fuzzy FTA for quantitative probability calculation, revealing the fire mechanisms in ships older than 20 years caused by mechanical fatigue and insulation aging. Similarly, the combination of D–S evidence theory and the Human Error Assessment and Reduction Technique (HEART) offers a new approach for assessing cargo tank crack risks in chemical tankers, using Bayesian networks to handle uncertainty in expert opinions while quantifying human error probability [34]. Research indicates that D–S evidence theory can provide a unified framework for modeling uncertain, incomplete, or even unknown information, effectively overcoming the limitations of traditional probabilistic reasoning [35]. In container transportation, the integration of Type-2 fuzzy sets with the Success Likelihood Index Method (SLIM) has further improved risk prediction accuracy, with its multi-layer fuzzy logic effectively addressing the quantification of subjective judgments [36].

Although significant progress has been made in handling data and relational uncertainties, current approaches still largely rely on methods such as the SAM or Bayesian networks [37–40]. As a result, model uncertainty—stemming from approximation calculations—remains a persistent challenge. A key limitation is the widespread dependence on defuzzification techniques to obtain crisp probability values [41–43]. A common defuzzification formula employed in the literature [39,44–46] is the Fuzzy Possibility Score (FPS) = [(c + d)^2^ - cd – (a + b)^2^ + ab]/ [3*(c + d-a-b)], where a, b, c, d are the four parameters of a trapezoidal fuzzy number. However, this method has an inherent flaw: it discards the shape information of the fuzzy number itself. For instance, four distinct trapezoidal fuzzy numbers—(0.2,0.4,0.6,1.0), (0.25,0.45,0.668,1.0), (0.15,0.35,0.535,1.0), and (0.3,0.5,0.738,1.0)—all yield an identical FPS value of 0.56. This result is clearly inconsistent with reality, demonstrating that traditional defuzzification causes distortion through information loss. The root cause of this problem is twofold: first, it maps distinct fuzzy numbers to the same crisp value, failing to preserve their essential differences; second, it reduces the nonlinear arithmetic of trapezoidal fuzzy numbers to linear operations, thereby misrepresenting the true mechanism of uncertainty propagation.

This practice often leads to the premature simplification of basic event probabilities, causing information distortion and reducing the reliability of outcomes [47–49]. The issue is particularly pronounced during nonlinear multiplication operations involving trapezoidal fuzzy numbers, where model uncertainty is further amplified [50,51].

This systematic oversight represents a critical gap in current trapezoidal fuzzy fault tree risk assessments. To overcome these limitations, we propose a novel algorithm that specifically targets model uncertainty by preserving the integrity of fuzzy information throughout the analysis. Unlike conventional approaches, our method avoids defuzzification entirely by building on the cut-set theorem and the representation theorem of fuzzy numbers. This allows the original trapezoidal fuzzy shape to be maintained across all calculation stages, thereby minimizing information distortion and curbing the propagation of uncertainty. Key innovations of the algorithm include: (1) high-precision computation that reduces approximation errors, (2) full retention of fuzzy information for more reliable outcomes, and (3) broad applicability across different fault tree configurations.

The main contributions of this work are twofold. First, we introduce an uncertainty-aware algorithm that maintains fuzzy information across the assessment process, offering a new perspective on model uncertainty reduction. Second, we demonstrate the algorithm’s practical effectiveness through case studies, underscoring its potential to improve the accuracy and reliability of risk assessments in complex engineering systems. The structure of the paper is as follows: the Preliminaries section introduces the necessary theoretical background, including trapezoidal fuzzy numbers, the cut-set theorem, and the representation theorem. The Proposed Algorithm section presents the derivation and implementation of the proposed algorithm. The Case Studies and Sensitivity Analysis section validates the method using real and literature-based case studies, and the Conclusions section summarizes the findings and suggests directions for future research.

## Preliminaries

### Trapezoidal fuzzy numbers

Fuzzy set theory, introduced by Zadeh [52,53], provides a mathematical framework for handling data uncertainty. A fuzzy set $\;\tilde{A}\;$ on a universe *X* is defined by a membership function $\;\mu_{\tilde{A}}(x)$: *X*→ [0,1], which quantifies the degree to which an element  x  belongs to $\;\tilde{A}$. This function can be represented using various types of fuzzy numbers, among which trapezoidal fuzzy numbers are widely used due to their practicality and versatility [54–56]. A trapezoidal fuzzy number is denoted as $\;\tilde{A}=(x^{\left(\text{1}\right)},\;x^{\left(\text{2}\right)},\;x^{\left(\text{3}\right)},\;x^{\left(\text{4}\right)})$, and its membership function is defined by Eq. (1) [57]:


$$\begin{array}{c} \mu_{\tilde{A}}(x)=\left\{ \begin{array}{cc} \frac{x-x^{\left(\text{1}\right)}}{x^{\left(\text{2}\right)}-x^{\left(\text{1}\right)}} & x^{\left(\text{1}\right)}\leq x\leq x^{\left(\text{2}\right)}\\ \text{1} & x^{\left(\text{2}\right)}\leq x\leq x^{\left(\text{3}\right)}\\ \frac{x^{\left(\text{4}\right)}-x}{x^{\left(\text{4}\right)}-x^{\left(\text{3}\right)}} & x^{\left(\text{3}\right)}\leq x\leq x^{\left(\text{4}\right)}\\ \text{0} & \text{otherwise} \end{array}\right.\; \end{array}$$ (1)

(1)
**Arithmetic operations of trapezoidal fuzzy numbers**

Let $\;{\tilde{A}}_{\text{1}}=({x^{\left(\text{1}\right)}}_{\text{1}},{x^{\left(\text{2}\right)}}_{\text{1}},{x^{\left(\text{3}\right)}}_{\text{1}},{x^{\left(\text{4}\right)}}_{\text{1}}$ and $\;{\tilde{A}}_{\text{2}}=({x^{\left(\text{1}\right)}}_{\text{2}},\;{x^{\left(\text{2}\right)}}_{\text{2}},{x^{\left(\text{3}\right)}}_{\text{2}},{x^{\left(\text{4}\right)}}_{\text{2}}$ be two trapezoidal fuzzy numbers. Their arithmetic operations are defined as follows [45]:


$${\tilde{A}}_{\text{1}}⊕{\tilde{A}}_{\text{2}}=\left({x^{\left(\text{1}\right)}}_{\text{1}}+{x^{\left(\text{1}\right)}}_{\text{2}},{x^{\left(\text{2}\right)}}_{\text{1}}+{x^{\left(\text{2}\right)}}_{\text{2}},{x^{\left(\text{3}\right)}}_{\text{1}}+{x^{\left(\text{3}\right)}}_{\text{2}},{x^{\left(\text{4}\right)}}_{\text{1}}+{x^{\left(\text{4}\right)}}_{\text{2}}\right)$$ (2)

$${\tilde{A}}_{\text{1}}⊖{\tilde{A}}_{\text{2}}=\left({x^{\left(\text{1}\right)}}_{\text{1}}-{x^{\left(\text{1}\right)}}_{\text{2}},{x^{\left(\text{2}\right)}}_{\text{1}}-{x^{\left(\text{2}\right)}}_{\text{2}},{x^{\left(\text{3}\right)}}_{\text{1}}-{x^{\left(\text{3}\right)}}_{\text{2}},{x^{\left(\text{4}\right)}}_{\text{1}}-{x^{\left(\text{4}\right)}}_{\text{2}}\right)$$ (3)

$${\tilde{A}}_{\text{1}}⊗{\tilde{A}}_{\text{2}}=({x^{\left(\text{1}\right)}}_{\text{1}}\times {x^{\left(\text{1}\right)}}_{\text{2}},{x^{\left(\text{2}\right)}}_{\text{1}}{\times x^{\left(\text{2}\right)}}_{\text{2}},{x^{\left(\text{3}\right)}}_{\text{1}}{{\times x}^{\left(\text{3}\right)}}_{\text{2}},{x^{\left(\text{4}\right)}}_{\text{1}}\times {x^{\left(\text{4}\right)}}_{\text{2}})$$ (4)

(2)
**Traditional Calculation of Top Event Probability**

Consider a basic event *i*, whose failure probability is represented by a trapezoidal fuzzy number $\;{\tilde{A}}_{i}=({x^{\left(\text{1}\right)}}_{i},{x^{\left(\text{2}\right)}}_{i},{x^{\left(\text{3}\right)}}_{i},{x^{\left(\text{4}\right)}}_{i})$. The conventional approach for calculating the top event probability in a trapezoidal fuzzy fault tree is as follows [58]:

For an AND gate:


$$q_{AND}=\prod_{i=\text{1}}^{n}{\tilde{A}}_{i}=(\prod_{i=\text{1}}^{n}{x^{\left(\text{1}\right)}}_{i},\;\prod_{i=\text{1}}^{n}{x^{\left(\text{2}\right)}}_{i},\;\prod_{i=\text{1}}^{n}{x^{\left(\text{3}\right)}}_{i},\;\prod_{i=\text{1}}^{n}{x^{\left(\text{4}\right)}}_{i})$$ (5)

For an OR gate:


$$q_{OR}=\text{1}⊖\prod_{i=\text{1}}^{n}\left(\text{1}⊖\tilde{A}\right)=\left[\text{1}-\prod_{i=\text{1}}^{n}\left(\text{1}-{x^{\left(\text{1}\right)}}_{i}\right),\text{1}-\prod_{i=\text{1}}^{n}\left(\text{1}-{x^{\left(\text{2}\right)}}_{i}\right),\text{1}-\prod_{i=\text{1}}^{n}\left(\text{1}-{x^{\left(\text{1}\right)}}_{i}\right),\;\text{1}-\prod_{i=\text{1}}^{n}\left(\text{1}-{x^{\left(\text{1}\right)}}_{i}\right)\right]$$ (6)

### cut-set theorem

For a fuzzy number $\;\tilde{A}(x$ and a parameter  λ(λ∈[0,1]), the λ-cut set is defined as:


$${\tilde{A}}_{\lambda }=\left\{x\in X|{\tilde{A}}_{\lambda }\geq \lambda \right\}$$ (7)

Here, λ is referred to as the cut level, threshold, or belief level. By varying the value λ, a fuzzy set can be transformed into a classical set.

### Representation theorem of fuzzy numbers

Consider a mapping:  [0,1]→F(X), that satisfies:


$$\forall \lambda_{\text{1}},\lambda_{\text{2}}\in [\text{0},\text{1}],\forall \lambda_{\text{1}}<\lambda_{\text{2}}\Rightarrow H\left(\lambda_{\text{1}}\right)\subseteq H\left(\lambda_{\text{2}}\right)$$ (8)

Then,  H  is called a nest of sets on  X.

Let $\;H:(\text{0},\text{1}]\to I_{R}$, with $\;\lambda \mapsto \tilde{A}(\lambda )=\left[m_{\lambda },n_{\lambda }\right]\neq \emptyset$, satisfy


$$\lambda_{\text{1}}<\lambda_{\text{2}}\Rightarrow \left[m_{\lambda_{\text{1}}},n_{\lambda_{\text{1}}}\right]⊇\left[m_{\lambda_{\text{2}}},n_{\lambda_{\text{2}}}\right].$$

Then, the fuzzy number $\;\tilde{A}\;$ can be represented as:


$$\tilde{A}={⋃}_{\lambda \in [\text{0},\text{1}]}\lambda H(\lambda )\in R$$ (9)

$${\tilde{A}}_{\lambda }={⋂}_{n=\text{1}}^{n=\infty }H\left(\lambda_{n}\right),\;\text{where}\;(\lambda >\text{0}),\left(\lambda_{n}=(\text{1}-\frac{\text{1}}{n+\text{1}}\right)\lambda )$$ (10)

$$\tilde{A}=(\left[m_{\tilde{A}},n_{\tilde{A}}\right],L_{\tilde{A}},R_{\tilde{A}})$$ (11)

where


$$m_{\tilde{A}}=\underset{n\to \infty }{\text{lim}}m_{\lambda_{n}}.$$ (12)

$$n_{\tilde{A}}=\underset{n\to \infty }{\text{lim}}n_{\lambda_{n}}.$$ (13)

$$L_{\tilde{A}}(x)={⋁}_{\text{0}<\lambda <\text{1}}\left\{\lambda |m_{\lambda }\leq x\right\}$$ (14)

$${\;R}_{\tilde{A}}(x)={⋁}_{\text{0}<\lambda <\text{1}}\left\{\lambda |n_{\lambda }\leq x\right\}$$ (15)

## A novel algorithm for precise trapezoidal fuzzy fault tree analysis

Building on the cut-set theorem and the representation theorem of trapezoidal fuzzy numbers, we propose a novel computational algorithm designed to significantly reduce model uncertainty in risk assessment. As illustrated in S1 Fig, the algorithm consists of five key steps, guiding the entire process from fuzzy number input to final risk assessment. This workflow preserves the integrity of fuzzy information while effectively controlling uncertainty propagation through precise computation.

**Step 1: Fault Tree Construction**

Construct the fault tree to identify basic events and their logical relationships leading to the top event. The probabilities of basic events are determined using the expert scoring method [59] and expressed as trapezoidal fuzzy numbers.

**Step 2: Logic Gate Classification**

As the types of logic gates directly influence the computational approach, the fault tree is classified into one of three categories prior to calculation:

Fault trees containing only AND gates (proceed to Step 3, Branch 1)

Fault trees containing only OR gates (proceed to Step 3, Branch 2)

Fault trees containing both AND and OR gates (proceed to Step 3, Branch 3)

**Step 3: Precise Computational Algorithm**

The following exact computation methods are applied according to the logic gate type:

**Branch 1: Fault trees with only AND gates**

If the top event consists of *n* basic events connected by an AND gate, and the failure probability of the *i*-th basic event is represented by a trapezoidal fuzzy number, then the λ-cut interval for the *i*-th basic event is given by Equation (16):


$$\left[{x^{\left(\text{1}\right)}}_{i}+\lambda ({x^{\left(\text{2}\right)}}_{i}-{x^{\left(\text{1}\right)}}_{i}),{x^{\left(\text{4}\right)}}_{i}+\lambda ({x^{\left(\text{3}\right)}}_{i}-{x^{\left(\text{4}\right)}}_{i})\right]$$ (16)

Then the *λ*-cut interval for the product of *n* basic events is:


$$\left[\prod_{i=\text{1}}^{i=n}\left({x^{\left(\text{1}\right)}}_{i}+\lambda \left({x^{\left(\text{2}\right)}}_{i}-{x^{\left(\text{1}\right)}}_{i}\right)\right),\prod_{i=\text{1}}^{i=n}\left({x^{\left(\text{4}\right)}}_{i}+\lambda ({x^{\left(\text{3}\right)}}_{i}-{x^{\left(\text{4}\right)}}_{i})\right)\right]$$ (17)

According to the representation theorem given in Equation (11), we obtain:


$${\tilde{A}}_{\text{and}}(x)=(\left[m_{{\tilde{A}}_{\text{and}}},n_{{\tilde{A}}_{\text{and}}}\right],L_{{\tilde{A}}_{\text{and}}}(x),R_{{\tilde{A}}_{\text{and}}}(x))$$ (18)

From Equation (7), it follows that:


$${{(m}_{{\tilde{A}}_{\text{and}}})}_{\lambda }=\prod_{i=\text{1}}^{i=n}\left({x^{\left(\text{1}\right)}}_{i}+\lambda \left({x^{\left(\text{2}\right)}}_{i}-{x^{\left(\text{1}\right)}}_{i}\right)\right)$$ (19)

$${{(n}_{{\tilde{A}}_{\text{and}}})}_{\lambda }=\prod_{i=\text{1}}^{i=n}\left({x^{\left(\text{4}\right)}}_{i}+\lambda \left({x^{\left(\text{3}\right)}}_{i}-{x^{\left(\text{4}\right)}}_{i}\right)\right)$$ (20)

Based on Equation (10), the values of $\;{{(m}_{{\tilde{A}}_{\text{and}}})}_{\lambda_{n}}$ and $\;{{(n}_{{\tilde{A}}_{\text{and}}})}_{\lambda_{n}}$ are derived as:


$${{(m}_{{\tilde{A}}_{\text{and}}})}_{\lambda_{n}}=\prod_{i=\text{1}}^{i=n}\left({x^{\left(\text{1}\right)}}_{i}+(\text{1}-\frac{\text{1}}{n+\text{1}})\left({x^{\left(\text{2}\right)}}_{i}-{x^{\left(\text{1}\right)}}_{i}\right)\right).$$ (21)

$${{(n}_{{\tilde{A}}_{\text{and}}})}_{\lambda_{n}}=\prod_{i=\text{1}}^{i=n}\left({x^{\left(\text{4}\right)}}_{i}+(\text{1}-\frac{\text{1}}{n+\text{1}})\left({x^{\left(\text{3}\right)}}_{i}-{x^{\left(\text{4}\right)}}_{i}\right)\right).$$ (22)

From Equation (12) and (13), the values of $\;m_{{\tilde{A}}_{\text{and}}}\;$ and $\;n_{{\tilde{A}}_{\text{and}}}\;$ are derived as:


$$m_{{\tilde{A}}_{\text{and}}}=\underset{n\to \infty }{\text{lim}}{{(m}_{{\tilde{A}}_{\text{and}}})}_{\lambda_{n}}=\underset{n\to \infty }{\text{lim}}\prod_{i=\text{1}}^{i=n}\left({x^{\left(\text{1}\right)}}_{i}+(\text{1}-\frac{\text{1}}{n+\text{1}})\left({x^{\left(\text{2}\right)}}_{i}-{x^{\left(\text{1}\right)}}_{i}\right)\right)=\prod_{i=\text{1}}^{i=n}{x^{\left(\text{2}\right)}}_{i}.$$ (23)

$$n_{{\tilde{A}}_{\text{and}}}=\underset{n\to \infty }{\text{lim}}{{(n}_{{\tilde{A}}_{\text{and}}})}_{\lambda_{n}}=\underset{n\to \infty }{\text{lim}}\prod_{i=\text{1}}^{i=n}\left({x^{\left(\text{4}\right)}}_{i}+(\text{1}-\frac{\text{1}}{n+\text{1}})\left({x^{\left(\text{3}\right)}}_{i}-{x^{\left(\text{4}\right)}}_{i}\right)\right)=\prod_{i=\text{1}}^{i=n}{x^{\left(\text{3}\right)}}_{i}.$$ (24)

Using Equation (14) and (15), the values of$\;L_{{\tilde{A}}_{\text{and}}}(x)$ and$\;R_{{\tilde{A}}_{\text{and}}}(x)$ are derived as:


$$L_{{\tilde{A}}_{\text{and}}}(x)={⋁}_{\text{0}<\lambda <\text{1}}\left\{\lambda |\prod_{i=\text{1}}^{i=n}\left({x^{\left(\text{1}\right)}}_{i}+\lambda \left({x^{\left(\text{2}\right)}}_{i}-{x^{\left(\text{1}\right)}}_{i}\right)\right)\leq x\right\}.$$ (25)

$$R_{{\tilde{A}}_{\text{and}}}(x)={⋁}_{\text{0}<\lambda <\text{1}}\left\{\lambda |\prod_{i=\text{1}}^{i=n}\left({x^{\left(\text{4}\right)}}_{i}+\lambda \left({x^{\left(\text{3}\right)}}_{i}-{x^{\left(\text{4}\right)}}_{i}\right)\right)\geq x\right\}.$$ (26)

Within the range of  λ (0, 1), all values of $\;{{(m}_{{\tilde{A}}_{\text{and}}})}_{\lambda }=\prod_{i=\text{1}}^{i=n}\left({x^{\left(\text{1}\right)}}_{i}+\lambda \left({x^{\left(\text{2}\right)}}_{i}-{x^{\left(\text{1}\right)}}_{i}\right)\right)$ and $\;{{(n}_{{\tilde{A}}_{\text{and}}})}_{\lambda }=\prod_{i=\text{1}}^{i=n}\left({x^{\left(\text{4}\right)}}_{i}+\lambda \left({x^{\left(\text{3}\right)}}_{i}-{x^{\left(\text{4}\right)}}_{i}\right)\right)$ are calculated. Since $\;{{(m}_{{\tilde{A}}_{\text{and}}})}_{\lambda }\;$ is a monotonically increasing function of  λ, for any given $\;x_{L\text{1}}(\text{0}\leq x_{L\text{1}}\leq \text{1})$, the maximum value of $\;{{(m}_{{\tilde{A}}_{\text{and}}})}_{\lambda }\;$ that is less than or equal to $\;x_{L\text{1}}\;$ can be determined. The corresponding value of  λ  is denoted as $\;\lambda_{L\text{1}}$, which allows the coordinates $\;(x_{L\text{1}},\lambda_{L\text{1}}$ on the left half of the precise membership curve of the trapezoidal fuzzy number to be determined. Subsequently, the precise membership curve of $\;L_{{\tilde{A}}_{\text{and}}}(x)\;$ can be derived.

Similarly, since $\;{{(n}_{{\tilde{A}}_{\text{and}}})}_{\lambda }=\prod_{i=\text{1}}^{i=n}\left({x^{\left(\text{4}\right)}}_{i}+\lambda \left({x^{\left(\text{3}\right)}}_{i}-{x^{\left(\text{4}\right)}}_{i}\right)\right)$ is a monotonically decreasing function of  λ  for any given $\;x_{R\text{1}}(\text{0}\leq x_{R\text{1}}\leq \text{1})$, the minimum value of $\;{{(n}_{{\tilde{A}}_{\text{and}}})}_{\lambda }\;$that is greater than or equal to $\;x_{R\text{1}}\;$ can be determined. The corresponding value of  λ  is denoted as $\;\lambda_{R\text{1}}$, which allows the coordinates $\;(x_{R\text{1}},\lambda_{R\text{1}}$ on the right half of the precise membership curve of the trapezoidal fuzzy number to be determined. Subsequently, the precise membership curve of $\;R_{{\tilde{A}}_{\text{and}}}(x)\;$ can be derived.

${{(m}_{{\tilde{A}}_{𝐚𝐧𝐝}})}_{\lambda }$ **monotonicity proof is detailed in** S1 Appendix

${{(n}_{{\tilde{A}}_{𝐚𝐧𝐝}})}_{\lambda }$ **monotonicity proof is detailed in** S2 Appendix

**Branch 2: Designed for fault trees consisting exclusively of OR gates.**

If *n* basic events of a fault tree are connected through an OR gate, the failure probability of the *i*-th basic event not occurring can be expressed as a trapezoidal fuzzy number ($\tilde{A}=({x^{\left(\text{1}\right)}}_{i},\;{x^{\left(\text{2}\right)}}_{i},\;{x^{\left(\text{3}\right)}}_{i},\;{x^{\left(\text{4}\right)}}_{i})$, according to the analysis of Branch 1. Based on Formula (7), the *λ* -cut interval of the *i*-th basic event is:


$${\overset{―}{\tilde{A}}}_{i}=\left(\text{1}-{x^{\left(\text{4}\right)}}_{i},\text{1}-{x^{\left(\text{3}\right)}}_{i},\text{1}-{x^{\left(\text{2}\right)}}_{i},\text{1}-{x^{\left(\text{1}\right)}}_{i}\right).$$ (27)

The *λ* -cut interval for the simultaneous non-occurrence of all *n* basic events is:


$$\left[\prod_{i=\text{1}}^{i=n}\left(\text{1}-{x^{\left(\text{4}\right)}}_{i}+\lambda ({x^{\left(\text{4}\right)}}_{i}-{x^{\left(\text{3}\right)}}_{i})\right),\prod_{i=\text{1}}^{i=n}\left(\text{1}-{x^{\left(\text{1}\right)}}_{i}+\lambda ({x^{\left(\text{1}\right)}}_{i}-{x^{\left(\text{2}\right)}}_{i})\right)\right].$$ (28)

Similar to the analysis of Branch 1, we can obtain:


$${\tilde{A}}_{\text{or}}(x)=(\left[m_{{\tilde{A}}_{\text{or}}},n_{{\tilde{A}}_{\text{or}}}\right],L_{{\tilde{A}}_{\text{or}}}(x),R_{{\tilde{A}}_{\text{or}}}(x)).$$ (29)

$${{(m}_{{\tilde{A}}_{\text{or}}})}_{\lambda }=\prod_{i=\text{1}}^{i=n}\left(\text{1}-{x^{\left(\text{4}\right)}}_{i}+\lambda ({x^{\left(\text{4}\right)}}_{i}-{x^{\left(\text{3}\right)}}_{i})\right).$$ (30)

$${{(n}_{{\tilde{A}}_{\text{or}}})}_{\lambda }=\prod_{i=\text{1}}^{i=n}\left(\text{1}-{x^{\left(\text{1}\right)}}_{i}+\lambda ({x^{\left(\text{1}\right)}}_{i}-{x^{\left(\text{2}\right)}}_{i})\right).$$ (31)

$${{(m}_{{\tilde{A}}_{\text{or}}})}_{\lambda_{n}}=\prod_{i=\text{1}}^{i=n}\left(\text{1}-{x^{\left(\text{4}\right)}}_{i}+(\text{1}-\frac{\text{1}}{n+\text{1}})\left({x^{\left(\text{4}\right)}}_{i}-{x^{\left(\text{3}\right)}}_{i}\right)\right)$$ (32)

$${{(n}_{{\tilde{A}}_{\text{or}}})}_{\lambda_{n}}=\prod_{i=\text{1}}^{i=n}\left(\text{1}-{x^{\left(\text{1}\right)}}_{i}+(\text{1}-\frac{\text{1}}{n+\text{1}})\left({x^{\left(\text{1}\right)}}_{i}-{x^{\left(\text{2}\right)}}_{i}\right)\right).$$ (33)

$$m_{{\tilde{A}}_{\text{or}}}=\underset{n\to \infty }{\text{lim}}{{(m}_{{\tilde{A}}_{\text{or}}})}_{\lambda_{n}}=\underset{n\to \infty }{\text{lim}}\prod_{i=\text{1}}^{i=n}\left(\text{1}-{x^{\left(\text{4}\right)}}_{i}+(\text{1}-\frac{\text{1}}{n+\text{1}})\left({x^{\left(\text{4}\right)}}_{i}-{x^{\left(\text{3}\right)}}_{i}\right)\right)=\prod_{i=\text{1}}^{i=n}\text{1}-{x^{\left(\text{3}\right)}}_{i}.$$ (34)

$$n_{{\tilde{A}}_{\text{or}}}=\underset{n\to \infty }{\text{lim}}{{(n}_{{\tilde{A}}_{\text{or}}})}_{\lambda_{n}}=\underset{n\to \infty }{\text{lim}}\prod_{i=\text{1}}^{i=n}\left(\text{1}-{x^{\left(\text{1}\right)}}_{i}+(\text{1}-\frac{\text{1}}{n+\text{1}})\left({x^{\left(\text{1}\right)}}_{i}-{x^{\left(\text{2}\right)}}_{i}\right)\right)=\prod_{i=\text{1}}^{i=n}\text{1}-{x^{\left(\text{2}\right)}}_{i}.$$ (35)

$$L_{{\tilde{A}}_{\text{or}}}(x)={⋁}_{\text{0}<\lambda <\text{1}}\left\{\lambda |\prod_{i=\text{1}}^{i=n}\left(\text{1}-{x^{\left(\text{4}\right)}}_{i}+\lambda ({x^{\left(\text{4}\right)}}_{i}-{x^{\left(\text{3}\right)}}_{i})\right)\leq x\right\}.$$ (36)

$$R_{{\tilde{A}}_{\text{or}}}(x)={⋁}_{\text{0}<\lambda <\text{1}}\left\{\lambda |\prod_{i=\text{1}}^{i=n}\left(\text{1}-{x^{\left(\text{1}\right)}}_{i}+\lambda ({x^{\left(\text{1}\right)}}_{i}-{x^{\left(\text{2}\right)}}_{i})\right)\geq x\right\}.$$ (37)

For any given $\;x_{L\text{2}}(\text{0}\leq x_{L\text{2}}\leq \text{1})$, the maximum value of $\;\prod_{i=\text{1}}^{i=n}(\text{1}-{x^{\left(\text{4}\right)}}_{i}+\lambda ({x^{\left(\text{4}\right)}}_{i}-{x^{\left(\text{3}\right)}}_{i})\backslash )$ satisfying $\;\prod_{i=\text{1}}^{i=n}\left(\text{1}-{x^{\left(\text{4}\right)}}_{i}+\lambda ({x^{\left(\text{4}\right)}}_{i}-{x^{\left(\text{3}\right)}}_{i})\right)\leq x_{L\text{2}}\;$ can be determined, and its corresponding  λ  can be represented by $\lambda_{L\text{2}}$ Thus, the coordinate values ($x_{L\text{2}},\lambda_{L\text{2}}$) on the left half of the precise membership curve of the trapezoidal fuzzy number can be determined, and the precise membership curve of $\;L_{{\tilde{A}}_{\text{or}}}(x)\;$ can be derived.

For any given $\;x_{R\text{2}}(\text{0}\leq x_{R\text{2}}\leq \text{1})$, the minimum value of $\;\prod_{i=\text{1}}^{i=n}(\text{1}-{x^{\left(\text{1}\right)}}_{i}+\lambda ({x^{\left(\text{1}\right)}}_{i}-{x^{\left(\text{2}\right)}}_{i})\backslash )$ satisfying $\;\prod_{i=\text{1}}^{i=n}\left(\text{1}-{x^{\left(\text{1}\right)}}_{i}+\lambda ({x^{\left(\text{1}\right)}}_{i}-{x^{\left(\text{2}\right)}}_{i})\right)\geq x_{R\text{2}}\;$ can be determined, and its corresponding  λ  can be represented by $\;\lambda_{R\text{2}}$. Thus, the coordinate values $\;(x_{R\text{2}},\lambda_{R\text{2}}$) on the right half of the precise membership curve of the trapezoidal fuzzy number can be determined, and the precise membership curve of $\;R_{{\tilde{A}}_{\text{or}}}(x)\;$ can be derived.

${{(m}_{{\tilde{A}}_{𝐨𝐫}})}_{\lambda }$ **monotonicity proof is detailed in** S3 Appendix

${{(n}_{{\tilde{A}}_{𝐨𝐫}})}_{\lambda }$ **monotonicity proof is detailed in** S4 Appendix

**Branch 3: Applicable to fault trees featuring a combination of AND gates and OR gates.**

If the fault tree consists of *n* basic events and includes both AND gates and OR gates, the *λ*-cut interval for the failure probability of the top event occurring can be expressed as follows, based on Formula (7):


$$\left[\sum_{k=\text{1}}^{k=n_{G}}\prod_{x_{i}\in G_{k}}\left({x^{\left(\text{1}\right)}}_{i}+\lambda \left({x^{\left(\text{2}\right)}}_{i}-{x^{\left(\text{1}\right)}}_{i}\right)\right),\sum_{k=\text{1}}^{k=n_{G}}\prod_{x_{i}\in G_{k}}\left({x^{\left(\text{4}\right)}}_{i}+\lambda ({x^{\left(\text{3}\right)}}_{i}-{x^{\left(\text{4}\right)}}_{i})\right)\right].$$ (38)

According to the representation theorem (11), the following can be derived:


$${\tilde{A}}_{\text{all}}(x)=(\left[m_{{\tilde{A}}_{\text{all}}},n_{{\tilde{A}}_{\text{all}}}\right],L_{{\tilde{A}}_{\text{all}}}(x),R_{{\tilde{A}}_{\text{all}}}(x)).$$ (39)

$${{(m}_{{\tilde{A}}_{\text{all}}})}_{\lambda }=\sum_{k=\text{1}}^{k=n_{G}}\prod_{x_{i}\in G_{k}}\left({x^{\left(\text{1}\right)}}_{i}+\lambda \left({x^{\left(\text{2}\right)}}_{i}-{x^{\left(\text{1}\right)}}_{i}\right)\right)$$ (40)

$${{(n}_{{\tilde{A}}_{\text{all}}})}_{\lambda }=\sum_{k=\text{1}}^{k=n_{G}}\prod_{x_{i}\in G_{k}}\left({x^{\left(\text{4}\right)}}_{i}+\lambda ({x^{\left(\text{3}\right)}}_{i}-{x^{\left(\text{4}\right)}}_{i})\right).$$ (41)

Using Formula (10), the values of parameters$\;{{(m}_{{\tilde{A}}_{\text{all}}})}_{\lambda_{n}}$and$\;{{(n}_{{\tilde{A}}_{\text{all}}})}_{\lambda_{n}}\;$can be obtained:


$${{(m}_{{\tilde{A}}_{\text{all}}})}_{\lambda_{n}}=\sum_{k=\text{1}}^{k=n_{G}}\prod_{x_{i}\in G_{k}}\left({x^{\left(\text{1}\right)}}_{i}+(\text{1}-\frac{\text{1}}{n+\text{1}})\left({x^{\left(\text{2}\right)}}_{i}-{x^{\left(\text{1}\right)}}_{i}\right)\right).$$ (42)

$${{(n}_{{\tilde{A}}_{\text{all}}})}_{\lambda_{n}}=\sum_{k=\text{1}}^{k=n_{G}}\prod_{x_{i}\in G_{k}}\left({x^{\left(\text{4}\right)}}_{i}+(\text{1}-\frac{\text{1}}{n+\text{1}})({x^{\left(\text{3}\right)}}_{i}-{x^{\left(\text{4}\right)}}_{i})\right).$$ (43)

Similarly, the values of parameters $\;m_{{\tilde{A}}_{\text{all}}}\;$and $\;n_{{\tilde{A}}_{\text{all}}}\;$can be derived from Formula (12) and (13):


$$m_{{\tilde{A}}_{\text{all}}}=\underset{n\to \infty }{\text{lim}}{{(m}_{{\tilde{A}}_{\text{all}}})}_{\lambda_{n}}=\underset{n\to \infty }{\text{lim}}\sum_{k=\text{1}}^{k=n_{G}}\prod_{x_{i}\in G_{k}}\left({x^{\left(\text{1}\right)}}_{i}+(\text{1}-\frac{\text{1}}{n+\text{1}})\left({x^{\left(\text{2}\right)}}_{i}-{x^{\left(\text{1}\right)}}_{i}\right)\right)=\sum_{k=\text{1}}^{k=n_{G}}\prod_{x_{i}\in G_{k}}{x^{\left(\text{2}\right)}}_{i}.$$ (44)

$$n_{{\tilde{A}}_{\text{all}}}=\underset{n\to \infty }{\text{lim}}{{(n}_{{\tilde{A}}_{\text{all}}})}_{\lambda_{n}}=\underset{n\to \infty }{\text{lim}}\sum_{k=\text{1}}^{k=n_{G}}\prod_{x_{i}\in G_{k}}\left({x^{\left(\text{4}\right)}}_{i}+(\text{1}-\frac{\text{1}}{n+\text{1}})({x^{\left(\text{3}\right)}}_{i}-{x^{\left(\text{4}\right)}}_{i})\right)=\sum_{k=\text{1}}^{k=n_{G}}\prod_{x_{i}\in G_{k}}{x^{\left(\text{3}\right)}}_{i}.$$ (45)

Furthermore, the values of parameters $\;L_{{\tilde{A}}_{\text{all}}}(x)\;$ and $\;R_{{\tilde{A}}_{\text{all}}}(x)\;$ can be determined using Formula (14) and (15):


$$L_{{\tilde{A}}_{\text{all}}}(x)={⋁}_{\text{0}<\lambda <\text{1}}\left\{\lambda |\sum_{k=\text{1}}^{k=n_{G}}\prod_{x_{i}\in G_{k}}\left({x^{\left(\text{1}\right)}}_{i}+\lambda \left({x^{\left(\text{2}\right)}}_{i}-{x^{\left(\text{1}\right)}}_{i}\right)\right)\leq x\right\}.$$ (46)

$$R_{{\tilde{A}}_{\text{all}}}(x)={⋁}_{\text{0}<\lambda <\text{1}}\left\{\lambda |\sum_{k=\text{1}}^{k=n_{G}}\prod_{x_{i}\in G_{k}}\left({x^{\left(\text{4}\right)}}_{i}+\lambda ({x^{\left(\text{3}\right)}}_{i}-{x^{\left(\text{4}\right)}}_{i})\right)\geq x\right\}.$$ (47)

**For the Left Half of the Membership Curve:**

Within the interval λ∈(0,1), all values of $\;{{(m}_{{\tilde{A}}_{\text{all}}})}_{\lambda }$= $\sum_{k=\text{1}}^{k=n_{G}}\prod_{x_{i}\in G_{k}}\left({x^{\left(\text{1}\right)}}_{i}+\lambda \left({x^{\left(\text{2}\right)}}_{i}-{x^{\left(\text{1}\right)}}_{i}\right)\right)\;$ and $\;{{(n}_{{\tilde{A}}_{\text{all}}})}_{\lambda }=\sum_{k=\text{1}}^{k=n_{G}}\prod_{x_{i}\in G_{k}}\left({x^{\left(\text{4}\right)}}_{i}+\lambda ({x^{\left(\text{3}\right)}}_{i}-{x^{\left(\text{4}\right)}}_{i})\right)$ are computed. Given that $\;{{(m}_{{\tilde{A}}_{\text{all}}})}_{\lambda }\;$ increases monotonically with  λ, for any specified $\;x_{L\text{3}}(\text{0}\leq x_{L\text{3}}\leq \text{1})$, the largest $\;{{(m}_{{\tilde{A}}_{\text{all}}})}_{\lambda }\;$ satisfying $\;{{(m}_{{\tilde{A}}_{\text{all}}})}_{\lambda }\leq x_{L\text{3}}\;$ can be determined. The corresponding  λ  is denoted as $\;\lambda_{L\text{2}}$, which enables the identification of the coordinates $\;(x_{L\text{3}},\lambda_{L\text{3}}$ on the left half of the precise membership curve for the trapezoidal fuzzy number. From these coordinates, the precise membership curve of $\;L_{{\tilde{A}}_{\text{all}}}(x)\;$ can be constructed.

**For the Right Half of the Membership Curve:**

On the other hand, since $\;{{(n}_{{\tilde{A}}_{\text{all}}})}_{\lambda }\;$ decreases monotonically with  λ, for any given $\;x_{R\text{3}}(\text{0}\leq x_{R\text{3}}\leq \text{1})$, the smallest $\;{{(n}_{{\tilde{A}}_{\text{all}}})}_{\lambda }\;$ satisfying $\;x_{R\text{3}}\geq {{(n}_{{\tilde{A}}_{\text{all}}})}_{\lambda }\;$ can be found. The corresponding  λ  is denoted as $\;\lambda_{R\text{3}}$, which facilitates the determination of the coordinates $\;(x_{R\text{3}},\lambda_{R\text{3}}$on the right half of the precise membership curve for the trapezoidal fuzzy number. Using these coordinates, the precise membership curve of $\;R_{{\tilde{A}}_{\text{all}}}(x)\;$ can be derived.

${{(m}_{{\tilde{A}}_{𝐚𝐥𝐥}})}_{\lambda }$ **monotonicity proof is detailed in** S5 Appendix

${{(n}_{{\tilde{A}}_{𝐚𝐥𝐥}})}_{\lambda }$ **monotonicity proof is detailed in** S6 Appendix

**Step 4: Membership Function Curve Plotting**

Based on the precise computational results, the membership function curves for the trapezoidal fuzzy fault tree are plotted, providing a visual representation of the fuzzy distribution of the top event failure probability.

(1) By connecting the points  (0,0), $\;(x_{L\text{11}},\lambda_{L\text{11}})$, $\;(x_{L\text{12}},\lambda_{L\text{12}})$, …, $\;(x_{L\text{1}e},\lambda_{L\text{1}e})$, $\;(\prod_{i=\text{1}}^{i=n}\text{1}-{x^{\left(\text{3}\right)}}_{i},\text{1})$, $\;(\prod_{i=\text{1}}^{i=n}\text{1}-{x^{\left(\text{2}\right)}}_{i},\text{1})$, $\;(x_{R\text{11}},\lambda_{R\text{11}})$, $\;(x_{R\text{12}},\lambda_{R\text{12}})$, …, $\;(x_{R\text{1}f},\lambda_{R\text{1}f})$, and  (0,0), the precise membership curve of the trapezoidal fuzzy fault tree composed of AND gates can be plotted.
(2) By linking the points (0,0), ($x_{L\text{21}},\lambda_{L\text{21}}$), ($x_{L\text{22}},\lambda_{L\text{22}}$), …, ($x_{L\text{2}g},\lambda_{L\text{2}g}$), ($\prod_{i=\text{1}}^{i=n}{x^{\left(\text{2}\right)}}_{i}$,1), ($\prod_{i=\text{1}}^{i=n}{x^{\left(\text{3}\right)}}_{i}$,1), $\;(x_{R\text{21}},\lambda_{R\text{21}}$), ($x_{R\text{22}},\lambda_{R\text{22}}$), …, ($x_{R\text{2}h},\lambda_{R\text{2}h}$), and (0,0), the precise membership curve of the trapezoidal fuzzy fault tree composed of OR gates can be constructed.
(3) By joining the points (0,0), ($x_{L\text{31}},\lambda_{L\text{31}}$), ($x_{L\text{32}},\lambda_{L\text{32}}$), …, ($x_{L\text{3}u},\lambda_{L\text{3}u}$), $\;(\sum_{k=\text{1}}^{k=n_{G}}\prod_{x_{i}\in G_{k}}{x^{\left(\text{2}\right)}}_{i},\text{1})$, ($\sum_{k=\text{1}}^{k=n_{G}}\prod_{x_{i}\in G_{k}}{x^{\left(\text{3}\right)}}_{i},\text{1}$), $\;(x_{R\text{31}},\lambda_{R\text{31}}$), $\;{(x}_{R\text{32}},\lambda_{R\text{32}})$, …, $\;(x_{R\text{3}v},\lambda_{R\text{3}v})$, and (0,0), the precise membership curve of the trapezoidal fuzzy fault tree composed of both AND and OR gates can be generated.

Here, the values of parameters *e*, *f*, *g*, ℎ, *u*, *v* are determined by the required analysis precision.

**Step 5: Risk Assessment**

The precise membership functions obtained in Step 4 provide a basis for predicting the top event’s failure probability and conducting importance analyses. These results can also be integrated with other risk assessment methods for further refinement.

## Comprehensive case studies and sensitivity analysis

### Validation and comparative analysis against benchmark literature data

To validate the reliability of the proposed algorithm and perform a comparative analysis against established methods, we replicated the electrostatic spark explosion case study from Zhang et al. [60]. This fault tree, containing both OR and AND gates across nine basic events, provides a robust benchmark for evaluating both accuracy and the ability to control model uncertainty.

To ensure a fair comparison, the normal fuzzy numbers from the reference study [60] were converted into trapezoidal fuzzy numbers based on the equal-area and centroid-equivalence principle [61]. The conversion results, summarized in Table 1 and visualized in S2 Fig, ensure the input data are equivalent while allowing us to apply our trapezoidal fuzzy-based algorithm.

**Table 1:** Conversion of Normal Fuzzy Numbers to Trapezoidal Fuzzy Numbers.

Basic event [60]	Mean of normal fuzzy numbers [60]	Variance of normal fuzzy numbers [60]	Area of normal fuzzy numbers	Mean of trapezoidal fuzzy numbers	Area of trapezoidal fuzzy numbers	Trapezoidal fuzzy numbers
***X***_**1**_	0.2500	0.1183	0.2091	0.2500	0.2091	[0.0528,0.2381,0.2619,0.4472]
***X***_**2**_	0.0038	0.0034	0.0060	0.0038	0.0060	[0,0.0035,0.0041,0.0095]
***X***_**3**_	0.5917	0.1069	0.1893	0.5917	0.1893	[0.4131,0.5810,0.6024,0.7703]
***X***_**4**_	0.4000	0.2366	0.4123	0.4000	0.4123	[0.0114,0.3763,0.4237,0.7886]
***X***_**5**_	0.5000	0.3317	0.5685	0.5000	0.5685	[0,0.4667,0.5333,1]
***X***_**6**_	0.0036	0.0038	0.0067	0.0036	0.0067	[0,0.0032,0.0040,0.0099]
***X***_**7**_	0.0233	0.0125	0.0222	0.0233	0.0222	[0.0024,0.0220,0.0246,0.0442]
***X***_**8**_	0.4750	0.2525	0.4450	0.4750	0.4450	[0.0553,0.4497,0.5003,0.8947]
***X***_**9**_	0.0950	0.00547	0.0097	0.0950	0.0097	[0.0858,0.0945,0.0955,0.1042]

The proposed algorithm was then applied to compute the top event probability and the fuzzy importance measures of basic events. Critically, to highlight the issue of model uncertainty, we compared our precise results against those generated by the traditional approximate method. The comparative results are detailed in Tables 2 and 3.

**Table 2:** Calculation results of top event failure probability.

Fault Tree category	Top event	Trapezoidal fuzzy number		Normal fuzzy number [60]		Similarity (Proposed algorithm)
		traditional method	Proposed algorithm	traditional method	Proposed algorithm	
**Tree1**	*X*_1_, *X*_2_, *X*_3_, *X*_4_, *X*_5_, *X*_6_, *X*_7_, *X*_8_, *X*_9_ occurred simultaneously	0.2216	0.2192	0.2145	0.2135	97.86%
**Tree2**	*X*_1_, *X*_2_, *X*_3_, *X*_4_, *X*_5_, *X*_6_, *X*_7_, *X*_8_ occurred simultaneously	0.8339	0.8682	0.8632	0.9335	99.42%
**Tree3**	*X*_2_, *X*_3_, *X*_4_, *X*_5_, *X*_6_, *X*_7_, *X*_8_ occurred simultaneously	0.8199	0.8535	0.8487	0.9154	99.44%
**Tree4**	*X*_1_, *X*_3_, *X*_4_, *X*_5_, *X*_6_, *X*_7_, *X*_8_ occurred simultaneously	0.8338	0.8680	0.8631	0.9333	99.44%
**Tree5**	*X*_1_, *X*_2_, *X*_4_, *X*_5_, *X*_6_, *X*_7_, *X*_8_ occurred simultaneously	0.7043	0.7555	0.7555	0.8494	100%
**Tree6**	*X*_1_, *X*_2_, *X*_3_, *X*_5_, *X*_6_, *X*_7_, *X*_8_ occurred simultaneously	0.8207	0.8500	0.8461	0.9015	99.54%
**Tree7**	X_1_, *X*_2_, *X*_3_, *X*_4_, *X*_6_, *X*_7_, *X*_8_ occurred simultaneously	0.8154	0.8425	0.8425	0.8910	100%
**Tree8**	*X*_1_, *X*_2_, *X*_3_, *X*_4_, *X*_5_, *X*_7_, *X*_8_ occurred simultaneously	0.8338	0.8681	0.8631	0.9333	99.44%
**Tree9**	*X*_1_, *X*_2_, *X*_3_, *X*_4_, *X*_5_, *X*_6_, *X*_8_ occurred simultaneously	0.8331	0.8670	0.8621	0.9332	99.43%
**Tree10**	*X*_1_, *X*_2_, *X*_3_, *X*_4_, *X*_5_, *X*_6_, *X*_7_ occurred simultaneously	0.8095	0.8388	0.8339	0.8904	99.42%

**Table 3:** Degree of Uncertainty Reduction in Trapezoidal Fuzzy Importance.

Basic event	Traditional method	Proposed algorithm	Degree of model uncertainty reduction	Basic event	Traditional method	Proposed algorithm	Model uncertainty reduction
***X***_**1**_	0.014	0.0147	5.00%	*X*_5_	0.0185	0.0257	38.92%
***X***_**2**_	0.0001	0.0002	100.00%	*X*_6_	0.0001	0.0002	100.00%
***X***_**3**_	0.1296	0.1127	9.70%	*X*_7_	0.0008	0.0012	50.00%
***X***_**4**_	0.0132	0.0182	37.88%	*X*_8_	0.0244	0.0294	20.49%

The data reveals a key finding: while the central values (e.g., fuzzy medians) between methods are close, the model uncertainty inherent in the traditional approximate method is substantial. Our algorithm consistently reduces this uncertainty, as quantified in Table 3, with reductions reaching up to 100% for some events and averaging 45.25%. This performance notably surpasses the 36.65% reduction reported in the benchmark study [60], underscoring the advantage of our approach in preserving information integrity.

This reduction effect is further visualized in S3 Fig to S12 Fig, which contrast the precise, well-defined trapezoidal outputs of our method against the distorted shapes produced by the traditional approximation.

The most comprehensive comparison is presented in S13 Fig. It demonstrates that the precise computational results from our method are in near-perfect agreement (99.40% consistency) with the precise results from the benchmark study. Conversely, the approximate results from both methods deviate significantly from their respective precise curves.

This divergence is not a discrepancy but a validation of our core thesis: model uncertainty introduced by approximation is a systematic error. The high agreement between the two precise methods confirms the correctness of our algorithm, while the larger uncertainty gap (blue shaded region) in the benchmark study underscores the superior ability of our method to mitigate this error. Therefore, the proposed algorithm not only validates successfully against a known benchmark but also provides a demonstrably more reliable and informative output for risk assessment.

### OREDA-based fault tree analysis: demonstrating robustness to system configuration changes

Building on the benchmark validation, this section employs real-world data from the OREDA handbook [62] to evaluate the algorithm’s performance under realistic conditions. The primary objective is to assess its robustness—specifically, how stable the algorithm’s output remains when key system parameters are varied. We begin by establishing a baseline using industrial valve failure data, a critical scenario in petrochemical industries.

#### Baseline analysis with OREDA data.

To ground the analysis in practical engineering, valve failure was selected as the top event, with nine associated basic events identified from the OREDA handbook. Table 4 presents the raw data, where each basic event’s failure probability is represented as a trapezoidal fuzzy number, forming the foundation for all subsequent sensitivity tests.

**Table 4:** Raw Data of Valve Failures from the OREDA Handbook [62].

Basic event	Failure causes	Failure rate	Central value of failure probability	Failure Probability of Trapezoidal Fuzzy Numbers
***E***_**1**_	Delayed operation	0.09	0.023	[0.01,0.022,0.024,0.09]
***E***_**2**_	External leakage-Process medium	0.35	0.088	[0.01,0.087,0.089,0.09]
***E***_**3**_	External leakage-Utility medium	0.06	0.016	[0.01,0.015,0.017,0.09]
***E***_**4**_	Fail to close on demand	4.24	0.672	[0.1,0.6620,0.6820,0.9]
***E***_**5**_	Fail to open on demand	2.66	0.503	[0.1,0.493,0.5130,0.9]
***E***_**6**_	Fail to regulate	2.28	0.451	[0.1,0.4410,0.4610,0.9]
***E***_**7**_	High output	0.03	0.008	[0.001,0.0079,0.0081,0.009]
***E***_**8**_	Internal leakage	0.09	0.023	[0.01,0.0220,0.0240,0.09]
***E***_**9**_	Spurious operation	0.73	0.175	[0.1,0.1650,0.1850,0.9]

#### Robustness to changes in system scale and complexity.

A key indicator of a robust algorithm is its ability to handle systems of varying scale without unpredictable performance degradation. To test this, we systematically increased the complexity of the fault tree by varying the number of basic events from 4 to 9. For each configuration, the precise result from the proposed algorithm was compared against the traditional approximate method.

**Results: Stable and Predictable Performance Enhancement**

The data in Table 5 reveals a crucial pattern: as system complexity increases, the proposed algorithm not only maintains stability but also delivers a progressively greater reduction in model uncertainty (from 1.20% to 7.49%). This trend is visualized in S14 Fig.

**Table 5:** Algorithm Performance with Increasing System Complexity (Number of Basic Events).

The number of basic events	Structure function	Proposed algorithm	Traditional method	Model uncertainty reduction
**4**	$T=E_{\text{1}}+E_{\text{2}}+E_{\text{3}}+E_{\text{4}}$	0.6190	0.6119	1.20%
**5**	$T=E_{\text{1}}+E_{\text{2}}+E_{\text{3}}+E_{\text{4}}+E_{\text{5}}$	0.7334	0.7162	2.40%
**6**	$T=E_{\text{1}}+E_{\text{2}}+E_{\text{3}}+E_{\text{4}}+E_{\text{5}}+E_{\text{6}}$	0.8068	0.7655	5.26%
**7**	$T=E_{\text{1}}+E_{\text{2}}+E_{\text{3}}+E_{\text{4}}+E_{\text{5}}+E_{\text{6}}+E_{\text{7}}$	0.8075	0.7660	5.27%
**8**	$T=E_{\text{1}}+E_{\text{2}}+E_{\text{3}}+E_{\text{4}}+E_{\text{5}}+E_{\text{6}}+E_{\text{7}}+E_{\text{8}}$	0.8093	0.7687	5.28%
**9**	$T=E_{\text{1}}+E_{\text{2}}+E_{\text{3}}+E_{\text{4}}+E_{\text{5}}+E_{\text{6}}+E_{\text{7}}+E_{\text{8}}+E_{\text{9}}$	0.8348	0.7943	7.49%

This demonstrates a critical aspect of robustness: graceful scaling. The algorithm consistently outperforms the traditional method, and the benefit of using the precise method becomes more pronounced for larger, more complex systems. This predictable enhancement—where increased complexity leads to greater relative accuracy—provides engineers with high confidence when applying the algorithm to real-world systems of unknown or variable scale. The algorithm effectively controls uncertainty propagation, ensuring reliable risk assessment even as the system model grows in complexity.

#### Robustness to variations in input fuzzy parameters.

To further interrogate the algorithm’s robustness, we tested its sensitivity to changes in the fundamental input parameters—the trapezoidal fuzzy numbers themselves. This assesses whether slight variations or uncertainties in defining the failure probabilities of basic events would lead to unstable or erratic outputs. We introduced an additional AND gate event, *Eor*0, into the valve failure fault tree (Structure: $T=\left(E_{\text{1}}+E_{\text{2}}+E_{\text{3}}+E_{\text{4}}+E_{\text{5}}+E_{\text{6}}+E_{\text{7}}+E_{\text{8}}+E_{\text{9}}\right)*E_{\text{or}\text{0}}$.) and systematically varied the centroid value of *Eor*0’s failure probability across two orders of magnitude.

**Results: Predictable Response and Consistent Superiority**

The results, detailed in Tables 6 and 7, demonstrate that the algorithm’s performance exhibits a highly stable and predictable pattern in response to input variations.

**Table 6:** Algorithm Performance with Varying AND Event Probability (Centroid 0.02 to 0.08).

Centroid values	Proposed algorithm	Traditional method	Model uncertainty reduction
$q_{𝐨𝐫0}=[0.01,0.029,0.031,0.09]$	0.1147	0.1282	10.6%
$q_{𝐨𝐫0}=[0.01,0.039,0.041,0.09]$	0.1222	0.1347	9.28%
$q_{𝐨𝐫0}=[0.01,0.049,0.051,0.09]$	0.1305	0.1427	8.55%
$q_{𝐨𝐫0}=[0.01,0.059,0.061,0.09]$	0.1393	0.1510	7.75%
$q_{𝐨𝐫0}=[0.01,0.069,0.071,0.09]$	0.1488	0.1598	6.89%
$q_{𝐨𝐫0}=[0.01,0.079,0.081,0.09]$	0.1589	0.1689	5.92%

**Table 7:** Algorithm Performance with Varying AND Event Probability (Centroid 0.003 to 0.008).

Centroid values	Proposed algorithm	Traditional method	Model uncertainty reduction
$q_{𝐨𝐫0}=[0.001,0.0029,0.0031,0.009]$	0.0115	0.0127	9.45%
$q_{𝐨𝐫0}=[0.001,0.0039,0.0041,0.009]$	0.0122	0.0135	8.89%
$q_{𝐨𝐫0}=[0.001,0.0049,0.0051,0.009]$	0.0130	0.0143	8.39%
$q_{𝐨𝐫0}=[0.001,0.0059,0.0061,0.009]$	0.0139	0.0151	7.28%
$q_{𝐨𝐫0}=[0.001,0.0069,0.0071,0.009]$	0.0148	0.0160	6.88%
$q_{𝐨𝐫0}=[0.001,0.0079,0.0081,0.009]$	0.0159	0.0169	5.92%

As shown in S15 Fig and S16 Fig, the algorithm consistently reduces model uncertainty across the entire tested range. A key finding is the smooth, monotonic response of the output to input changes. While the absolute value of the top event probability reasonably increases with the AND event’s probability, the uncertainty reduction effect provided by our algorithm remains substantial and stable.

Based on the data presented in Table 7, a graph illustrating the reduction in uncertainty is plotted, as shown in S16 Fig.

Critically, the data reveals that the algorithm’s advantage over the traditional method is preserved regardless of the input probability’s magnitude. The difference in results between the precise and traditional methods follows a consistent pattern, and the percentage of uncertainty reduction remains significant. This demonstrates that the algorithm is not overly sensitive to the specific values of input parameters. It performs reliably whether the additional event has a high or low probability, confirming its robust stability and suitability for assessing systems with diverse risk levels.

**Robustness to Combined Variations in System Scale and Input Parameters**

The most rigorous test of an algorithm’s robustness is its performance under combined variations of multiple parameters. This section investigates the algorithm’s response to the simultaneous increase in system complexity (by adding AND gates) and variation in input characteristics (by changing the centroid values of the added AND events from 0.2 to 0.8). This two-dimensional sensitivity analysis probes the algorithm’s stability boundaries.

The results, detailed across Tables 8–14, reveal a remarkable finding: the algorithm’s effectiveness in reducing model uncertainty remains highly stable and consistently superior despite the concurrent changes in both scale and input parameters.

**Table 8:** Algorithm Performance with Increasing AND Gates and Varying Centroid Values (centroid value of 0.2).

Structure Function	Proposed algorithm	Traditional method	Model uncertainty reduction
$T=\left(E_{1}+E_{2}+E_{3}+E_{4}+E_{5}+E_{6}+E_{7}+E_{8}+E_{9}\right)*E_{𝐨𝐫1}*E_{𝐨𝐫2}$	0.0899	0.1006	10.63%
$T=\left(E_{1}+E_{2}+E_{3}+E_{4}+E_{5}+E_{6}+E_{7}+E_{8}+E_{9}\right)*E_{𝐨𝐫1}*E_{𝐨𝐫2}*E_{𝐨𝐫2}$	0.0770	0.0860	10.46%
$T=\left(E_{1}+E_{2}+E_{3}+E_{4}+E_{5}+E_{6}+E_{7}+E_{8}+E_{9}\right)*E_{𝐨𝐫1}*E_{𝐨𝐫2}*E_{𝐨𝐫2}*E_{𝐨𝐫2}$	0.0687	0.0765	10.19%
$T=\left(E_{1}+E_{2}+E_{3}+E_{4}+E_{5}+E_{6}+E_{7}+E_{8}+E_{9}\right)*E_{𝐨𝐫1}*E_{𝐨𝐫2}*E_{𝐨𝐫2}*E_{𝐨𝐫2}*E_{𝐨𝐫2}$	0.0616	0.0685	10.07%
$𝐓=\left(E_{1}+E_{2}+E_{3}+E_{4}+E_{5}+E_{6}+E_{7}+E_{8}+E_{9}\right)*E_{𝐨𝐫1}*E_{𝐨𝐫2}*E_{𝐨𝐫2}*E_{𝐨𝐫2}*E_{𝐨𝐫2}*E_{𝐨𝐫2}$	0.056	0.0617	9.23%

**Table 9:** Algorithm Performance with Increasing AND Gates and Varying Centroid Values (centroid value of 0.3).

Structure Function	Proposed algorithm	Traditional method	Model uncertainty reduction
$T=\left(E_{1}+E_{2}+E_{3}+E_{4}+E_{5}+E_{6}+E_{7}+E_{8}+E_{9}\right)*E_{𝐨𝐫1}*E_{𝐨𝐫3}$	0.0927	0.1043	11.12%
$T=\left(E_{1}+E_{2}+E_{3}+E_{4}+E_{5}+E_{6}+E_{7}+E_{8}+E_{9}\right)*E_{𝐨𝐫1}*E_{𝐨𝐫3}*E_{𝐨𝐫3}$	0.0783	0.0875	10.51%
$T=\left(E_{1}+E_{2}+E_{3}+E_{4}+E_{5}+E_{6}+E_{7}+E_{8}+E_{9}\right)*E_{𝐨𝐫1}*E_{𝐨𝐫3}*E_{𝐨𝐫3}*E_{𝐨𝐫3}$	0.0691	0.0771	10.37%
$T=\left(E_{1}+E_{2}+E_{3}+E_{4}+E_{5}+E_{6}+E_{7}+E_{8}+E_{9}\right)*E_{𝐨𝐫1}*E_{𝐨𝐫3}*E_{𝐨𝐫3}*E_{𝐨𝐫3}*E_{𝐨𝐫3}$	0.0617	0.0688	10.31%
$𝐓=\left(E_{1}+E_{2}+E_{3}+E_{4}+E_{5}+E_{6}+E_{7}+E_{8}+E_{9}\right)*E_{𝐨𝐫1}*E_{𝐨𝐫3}*E_{𝐨𝐫3}*E_{𝐨𝐫3}*E_{𝐨𝐫3}*E_{𝐨𝐫3}$	0.0555	0.0618	10.19%

**Table 10:** Algorithm Performance with Increasing AND Gates and Varying Centroid Values (centroid value of 0.4).

Structure Function	Proposed algorithm	Traditional method	Model uncertainty reduction
$T=\left(E_{1}+E_{2}+E_{3}+E_{4}+E_{5}+E_{6}+E_{7}+E_{8}+E_{9}\right)*E_{𝐨𝐫1}*E_{𝐨𝐫4}$	0.0960	0.1080	11.11%
$T=\left(E_{1}+E_{2}+E_{3}+E_{4}+E_{5}+E_{6}+E_{7}+E_{8}+E_{9}\right)*E_{𝐨𝐫1}*E_{𝐨𝐫4}*E_{𝐨𝐫4}$	0.0801	0.0900	11.00%
$T=\left(E_{1}+E_{2}+E_{3}+E_{4}+E_{5}+E_{6}+E_{7}+E_{8}+E_{9}\right)*E_{𝐨𝐫1}*E_{𝐨𝐫4}*E_{𝐨𝐫4}*E_{𝐨𝐫4}$	0.0699	0.0781	10.49%
$T=\left(E_{1}+E_{2}+E_{3}+E_{4}+E_{5}+E_{6}+E_{7}+E_{8}+E_{9}\right)*E_{𝐨𝐫1}*E_{𝐨𝐫4}*E_{𝐨𝐫4}*E_{𝐨𝐫4}$	0.0621	0.0693	10.38%
$𝐓=\left(E_{1}+E_{2}+E_{3}+E_{4}+E_{5}+E_{6}+E_{7}+E_{8}+E_{9}\right)*E_{𝐨𝐫1}*E_{𝐨𝐫4}*E_{𝐨𝐫4}*E_{𝐨𝐫4}*E_{𝐨𝐫4}*E_{𝐨𝐫4}$	0.0556	0.0620	10.32%

**Table 11:** Algorithm Performance with Increasing AND Gates and Varying Centroid Values (centroid value of 0.5).

Structure Function	Proposed algorithm	Traditional method	Model uncertainty reduction
$T=\left(E_{1}+E_{2}+E_{3}+E_{4}+E_{5}+E_{6}+E_{7}+E_{8}+E_{9}\right)*E_{𝐨𝐫1}*E_{𝐨𝐫5}$	0.0994	0.1116	10.93%
$T=\left(E_{1}+E_{2}+E_{3}+E_{4}+E_{5}+E_{6}+E_{7}+E_{8}+E_{9}\right)*E_{𝐨𝐫1}*E_{𝐨𝐫5}*E_{𝐨𝐫5}$	0.0827	0.0930	11.4%
$T=\left(E_{1}+E_{2}+E_{3}+E_{4}+E_{5}+E_{6}+E_{7}+E_{8}+E_{9}\right)*E_{𝐨𝐫1}*E_{𝐨𝐫5}*E_{𝐨𝐫5}$	0.0716	0.0804	10.94%
$T=\left(E_{1}+E_{2}+E_{3}+E_{4}+E_{5}+E_{6}+E_{7}+E_{8}+E_{9}\right)*E_{𝐨𝐫1}*E_{𝐨𝐫5}*E_{𝐨𝐫5}*E_{𝐨𝐫5}*E_{𝐨𝐫5}$	0.0631	0.0706	10.62%
$𝐓=\left(E_{1}+E_{2}+E_{3}+E_{4}+E_{5}+E_{6}+E_{7}+E_{8}+E_{9}\right)*E_{𝐨𝐫1}*E_{𝐨𝐫5}*E_{𝐨𝐫5}*E_{𝐨𝐫5}*E_{𝐨𝐫5}*E_{𝐨𝐫5}$	0.0561	0.0627	10.52%

**Table 12:** Algorithm Performance with Increasing AND Gates and Varying Centroid Values (centroid value of 0.6).

Structure Function	Proposed algorithm	Traditional method	Model uncertainty reduction
$T=\left(E_{\text{1}}+E_{\text{2}}+E_{\text{3}}+E_{\text{4}}+E_{\text{5}}+E_{\text{6}}+E_{\text{7}}+E_{\text{8}}+E_{\text{9}}\right)*E_{\text{or}\text{1}}*E_{\text{or}\text{6}}$	0.1029	0.1153	10.75%
$T=\left(E_{\text{1}}+E_{\text{2}}+E_{\text{3}}+E_{\text{4}}+E_{\text{5}}+E_{\text{6}}+E_{\text{7}}+E_{\text{8}}+E_{\text{9}}\right)*E_{\text{or}\text{1}}*E_{\text{or}\text{6}}*E_{\text{or}\text{6}}$	0.0861	0.0969	11.14%
$T=\left(E_{\text{1}}+E_{\text{2}}+E_{\text{3}}+E_{\text{4}}+E_{\text{5}}+E_{\text{6}}+E_{\text{7}}+E_{\text{8}}+E_{\text{9}}\right)*E_{\text{or}\text{1}}*E_{\text{or}\text{6}}*E_{\text{or}\text{6}}*E_{\text{or}\text{6}}$	0.0740	0.0832	11.06%
$T=\left(E_{\text{1}}+E_{\text{2}}+E_{\text{3}}+E_{\text{4}}+E_{\text{5}}+E_{\text{6}}+E_{\text{7}}+E_{\text{8}}+E_{\text{9}}\right)*E_{\text{or}\text{1}}*E_{\text{or}\text{6}}*E_{\text{or}\text{6}}*E_{\text{or}\text{6}}*E_{\text{or}\text{6}}$	0.0647	0.0726	10.90%
$\text{T}=\left(E_{\text{1}}+E_{\text{2}}+E_{\text{3}}+E_{\text{4}}+E_{\text{5}}+E_{\text{6}}+E_{\text{7}}+E_{\text{8}}+E_{\text{9}}\right)*E_{\text{or}\text{1}}*E_{\text{or}\text{6}}*E_{\text{or}\text{6}}*E_{\text{or}\text{6}}*E_{\text{or}\text{6}}*E_{\text{or}\text{6}}$	0.0572	0.0642	10.90%

**Table 13:** Algorithm Performance with Increasing AND Gates and Varying Centroid Values (centroid value of 0.7).

Structure Function	Proposed algorithm	Traditional method	Model uncertainty reduction
$T=\left(E_{\text{1}}+E_{\text{2}}+E_{\text{3}}+E_{\text{4}}+E_{\text{5}}+E_{\text{6}}+E_{\text{7}}+E_{\text{8}}+E_{\text{9}}\right)*E_{\text{or}\text{1}}*E_{\text{or}\text{7}}$	0.1069	0.1192	10.32%
$T=\left(E_{\text{1}}+E_{\text{2}}+E_{\text{3}}+E_{\text{4}}+E_{\text{5}}+E_{\text{6}}+E_{\text{7}}+E_{\text{8}}+E_{\text{9}}\right)*E_{\text{or}\text{1}}*E_{\text{or}\text{7}}*E_{\text{or}\text{7}}$	0.0907	0.1018	10.90%
$T=\left(E_{\text{1}}+E_{\text{2}}+E_{\text{3}}+E_{\text{4}}+E_{\text{5}}+E_{\text{6}}+E_{\text{7}}+E_{\text{8}}+E_{\text{9}}\right)*E_{\text{or}\text{1}}*E_{\text{or}\text{7}}*E_{\text{or}\text{7}}*Y_{\text{or}\text{7}}$	0.0781	0.0878	11.05%
$T=\left(E_{\text{1}}+E_{\text{2}}+E_{\text{3}}+E_{\text{4}}+E_{\text{5}}+E_{\text{6}}+E_{\text{7}}+E_{\text{8}}+E_{\text{9}}\right)*E_{\text{or}\text{1}}*E_{\text{or}\text{7}}*E_{\text{or}\text{7}}*Y_{\text{or}\text{7}}*E_{\text{or}\text{7}}$	0.0678	0.0764	11.25%
$\text{T}=\left(E_{\text{1}}+E_{\text{2}}+E_{\text{3}}+E_{\text{4}}+E_{\text{5}}+E_{\text{6}}+E_{\text{7}}+E_{\text{8}}+E_{\text{9}}\right)*E_{\text{or}\text{1}}*E_{\text{or}\text{7}}*E_{\text{or}\text{7}}*Y_{\text{or}\text{7}}*E_{\text{or}\text{7}}*E_{\text{or}\text{7}}$	0.0595	0.0671	11.33%

**Table 14:** Algorithm Performance with Increasing AND Gates and Varying Centroid Values (centroid value of 0.8).

Structure Function	Proposed algorithm	Traditional method	Model uncertainty reduction
$T=\left(E_{\text{1}}+E_{\text{2}}+E_{\text{3}}+E_{\text{4}}+E_{\text{5}}+E_{\text{6}}+E_{\text{7}}+E_{\text{8}}+E_{\text{9}}\right)*E_{\text{or}\text{1}}*E_{\text{or}\text{8}}$	0.1109	0.1231	9.91%
$T=\left(E_{\text{1}}+E_{\text{2}}+E_{\text{3}}+E_{\text{4}}+E_{\text{5}}+E_{\text{6}}+E_{\text{7}}+E_{\text{8}}+E_{\text{9}}\right)*E_{\text{or}\text{1}}*E_{\text{or}\text{8}}*E_{\text{or}\text{8}}$	0.0963	0.1075	10.42%
$T=\left(E_{\text{1}}+E_{\text{2}}+E_{\text{3}}+E_{\text{4}}+E_{\text{5}}+E_{\text{6}}+E_{\text{7}}+E_{\text{8}}+E_{\text{9}}\right)*E_{\text{or}\text{1}}*E_{\text{or}\text{8}}*E_{\text{or}\text{8}}*E_{\text{or}\text{8}}$	0.0842	0.0940	10.43%
$T=\left(E_{\text{1}}+E_{\text{2}}+E_{\text{3}}+E_{\text{4}}+E_{\text{5}}+E_{\text{6}}+E_{\text{7}}+E_{\text{8}}+E_{\text{9}}\right)*E_{\text{or}\text{1}}*E_{\text{or}\text{8}}*E_{\text{or}\text{8}}*E_{\text{or}\text{8}}*E_{\text{or}\text{8}}$	0.0739	0.0826	10.53%
$\text{T}=\left(E_{\text{1}}+E_{\text{2}}+E_{\text{3}}+E_{\text{4}}+E_{\text{5}}+E_{\text{6}}+E_{\text{7}}+E_{\text{8}}+E_{\text{9}}\right)*E_{\text{or}\text{1}}*E_{\text{or}\text{8}}*E_{\text{or}\text{8}}*E_{\text{or}\text{8}}*E_{\text{or}\text{8}}*E_{\text{or}\text{8}}$	0.0650	0.0728	10.71%

The data is synthesized in S17 Fig, which visually consolidates the results from all tested scenarios.

The most significant conclusion from this comprehensive analysis is the narrow range of performance variation. As the system scales up in complexity and the input parameters vary widely, the model uncertainty reduction effect remains tightly bounded between 9.23% and 11.33%. This indicates that the algorithm’s advantage is not contingent on a specific system configuration or input value. It delivers reliable, high-quality results across a broad spectrum of conditions.

This exceptional stability demonstrates that whether dealing with a few or many AND events, and whether those events have high or moderate probability, the algorithm consistently controls uncertainty propagation. This makes it particularly valuable for modeling large, complex systems where the exact number of components and their failure probabilities may be subject to uncertainty, providing “robust theoretical support” for safety-critical decision-making.

### Robustness verification under input uncertainties

#### Methodology: Probability perturbation approach.

To evaluate robustness, we selected representative events from the OREDA dataset with high, medium, and low failure probabilities: E4 (central value: 0.672, high probability), E5 (central value: 0.503, medium probability), and E7 (central value: 0.008, low probability).

For each event, random perturbations of ±5%, ± 10%, and ±15% were applied to its central probability value while preserving the trapezoidal fuzzy number structure. At each perturbation level, 20 independent trials were conducted using different random seeds. The Uncertainty Reduction (UR) metric was recorded for each trial, and statistical analysis (mean, standard deviation, range) was performed to quantify robustness.

The complete dataset comprising 60 perturbation tests, including all input values and corresponding UR results for events E4, E5, and E7, is provided in S1, S2, S3, S4, S5, S6, S7, S8, S9 Tables to ensure transparency and reproducibility.

#### Results for high-probability events (E4).

The uncertainty reduction performance for the high-probability event E4 under different perturbation levels is presented in Table 15.

**Table 15:** Uncertainty reduction performance under probability perturbations for event E4.

Perturbation Level	Mean UR (%)	Std. Dev.	Min (%)	Max (%)	Range (%)
±5%	2.3570%	0.0407%	2.3000%	2.4200%	0.1200%
±10%	2.3480%	0.0918%	2.1700%	2.5600%	0.3900%
±15%	2.3135%	0.1868%	2.0600%	2.7600%	0.7000%

The algorithm demonstrated stability under mild perturbations (±5%). As the perturbation magnitude increased, the variability in results also increased, yet remained within acceptable limits. The algorithm exhibited adaptability: output fluctuations grew with larger input perturbations, but without performance collapse. A positive correlation exists between perturbation level and output variability. Notably, even under ±15% perturbation, the standard deviation remained relatively low.

#### Results for medium-probability events (E5).

Table 16 presents the uncertainty reduction performance for the medium-probability event E5, which demonstrated high stability across all perturbation levels.

**Table 16:** Uncertainty reduction performance under probability perturbations for event E5.

Perturbation Level	Mean UR (%)	Std. Dev.	Min (%)	Max (%)	Range (%)
**±5%**	2.8705%	0.0545%	2.7900%	3.0100%	0.2200%
**±10%**	2.8485%	0.0685%	2.7800%	3.0200%	0.2400%
**±15%**	2.8990%	0.0835%	2.7300%	3.0900%	0.3600%

The algorithm showed consistent stability across all perturbation levels for event E5, indicating strong robustness for medium-probability events. It adapted well to perturbations, with output variability increasing only slightly as the perturbation magnitude grew, remaining within a small range. The positive correlation between perturbation level and output variability was observed, but the standard deviation stayed low (e.g., 0.0835% at ±15%). While the distribution of results became slightly more dispersed with higher perturbations, it maintained good consistency and symmetry. These results underscore the algorithm’s excellent capability in handling medium-probability events, which are often the most common fault type in practical engineering applications.

#### Results for low-probability events (E7).

The results for the low-probability event E7 (Table 17) reveal the algorithm’s exceptional robustness.

**Table 17:** Uncertainty reduction performance under probability perturbations for event E7.

Perturbation Level	Mean UR (%)	Std. Dev.	Min (%)	Max (%)	Range (%)
±5%	5.2340%	0.0060%	5.2200%	5.2400%	0.0200%
±10%	5.2320%	0.0106%	5.2200%	5.2500%	0.0300%
±15%	5.2300%	0.0108%	5.2200%	5.2500%	0.0300%

For the low-probability event E7, the algorithm exhibited exceptional stability. Under input perturbations ranging from ±5% to ±15%, the mean UR values remained tightly clustered between 5.230% and 5.234%, with extremely low standard deviations (< 0.00011). This indicates that the algorithm provides highly reliable and consistent results even for low-probability events, which are often challenging to measure precisely, demonstrating very strong robustness.

#### Comparative stability analysis.

The algorithm is not entirely insensitive to input perturbations, but its response is moderate and largely linear. The fluctuation in output results is significantly smaller than the input perturbation (e.g., a ± 15% input perturbation resulted in less than ±1% output fluctuation). This demonstrates the algorithm’s effective inherent capability to suppress the propagation of input uncertainty, meeting the robustness requirements for industrial applications.

The algorithm’s robustness is not uniform across all inputs; instead, it intelligently adjusts its sensitivity based on the event’s intrinsic importance within the system, as reflected by its base probability. This characteristic is a significant advantage for practical engineering applications. It implies that careful data collection remains crucial for high-probability components, while the algorithm can tolerate greater data uncertainty for numerous low-probability components. This effectively reduces the data acquisition and precision requirements, thereby lowering the overall cost of system modeling.

## Conclusions

This study has introduced a novel computational algorithm designed to tackle the persistent challenge of model uncertainty in trapezoidal fuzzy fault tree analysis. By leveraging the cut-set theorem and operating directly on trapezoidal fuzzy numbers without intermediate defuzzification, the proposed method effectively preserves information integrity and minimizes distortion throughout the uncertainty propagation process. The algorithm provides a generalized framework applicable to fault trees with OR-gates, AND-gates, and mixed logics.

The primary contribution of this work is the development of a precise computational framework that addresses a critical gap in existing methodologies. Comprehensive validation demonstrates the algorithm’s significant advantages. In the benchmark case study, it achieved a superior model uncertainty reduction of 45.25% compared to 36.65% from existing methods, while maintaining a 99.40% consistency with established precise results, confirming its accuracy. Furthermore, extensive simulations based on OREDA data robustly verified its stability and scalability. The algorithm exhibits a “graceful scaling” property, where the benefit of uncertainty reduction becomes more pronounced as system complexity increases (e.g., from 2.40% to 7.49% with growing basic events in OR-gate trees).

A pivotal finding is the algorithm’s exceptional robustness under input uncertainties. The dedicated perturbation analysis (60 tests across events with high, medium, and low probabilities) revealed that output variations remain remarkably small (<±1%) even under significant input perturbations (±15%). This controlled sensitivity, which is intelligently aligned with an event’s base probability (higher robustness for low-probability events), is a key practical advantage. It implies that rigorous data collection can be focused on critical high-probability components, while the algorithm reliably tolerates greater uncertainty for numerous low-probability ones, thereby reducing the overall cost and effort of system risk modeling.

In conclusion, this algorithm offers not only a theoretical advancement in managing model uncertainty but also a practical, robust, and efficient tool for risk assessment in complex engineering systems. Future work will focus on optimizing the computational efficiency for large-scale fault trees and exploring integrations with dynamic models like Bayesian networks to handle time-evolving uncertainties.

## Supporting information

S1 Fig The workflow commences with (1) Fault Tree Construction and subsequently proceeds to (2) Logic Gate Classification, which directs the flow to one of three precise computational branches tailored for AND-gate only, OR-gate only, or mixed-gate fault trees. The process continues with (4) Membership Function Curve Plotting and concludes with (5) Risk Assessment.
(TIF)

S2 Fig Each subplot (A-I) displays the conversion result for a respective basic event. **In each subplot, the original normal fuzzy number is shown as a red dashed line, while the converted trapezoidal fuzzy number is represented by a solid blue line. A blue dashed vertical line indicates the shared fuzzy median value for both distributions in each case. The conversion is based on the principle of equivalent area and identical fuzzy median between the original and converted forms, with the area (S) and median value for each basic event provided in the Table 1**.
(TIF)

S3 Fig.
**(A) Result from the proposed precise algorithm, showing the membership function as a red solid line. The fuzzy median (m**_**TXTAe**_ **) is indicated by the intersection of the black dashed line (area bisector) with the x-axis. (B) Result from the traditional approximate method, showing the distorted membership function as a blue dashed line and its fuzzy median (m**_**TXTAe’**_ **) via a green dashed area bisector. (C) Overlay of (A) and (B), demonstrating the reduction in model uncertainty. The distance between the black dashed line and the green dashed line (m**_**TXTAe**_ **= 0.2192 vs m**_**TXTAe’**_ **= 0.2216) represents the absolute value of the reduction in model uncertainty.**
(TIF)

S4 Fig The subplots compare the outputs of the proposed and traditional algorithms, following the same schema as S3 Fig. **(A) Result from the proposed precise algorithm (red solid line) with its fuzzy median (m**_**TXTBe**_ **= 0.8682). (B) Result from the traditional approximate method (blue dashed line) with its fuzzy median (m**_**TXTBe’**_ **= 0.8339). (C) Overlay of both results, where the difference between the fuzzy medians (Δm = 0.0343) quantifies the reduction in model uncertainty achieved by the proposed algorithm.**
(TIF)

S5 Fig The subplots compare the outputs of the proposed and traditional algorithms, following the same schema as S3 Fig. **(A) Result from the proposed precise algorithm (red solid line) with its fuzzy median (m**_**TX1e**_ **= 0.8535). (B) Result from the traditional approximate method (blue dashed line) with its fuzzy median (m**_**TX1e’**_ **= 0.8199). (C) Overlay of both results, where the difference between the fuzzy medians (Δm = 0.0336) quantifies the reduction in model uncertainty achieved by the proposed algorithm.**
(TIF)

S6 Fig The subplots compare the outputs of the proposed and traditional algorithms, following the same schema as S3 Fig. **(A) Result from the proposed precise algorithm (red solid line) with its fuzzy median (m**_**TX2e**_ **= 0.8680). (B) Result from the traditional approximate method (blue dashed line) with its fuzzy median (m**_**TX2e’**_ **= 0.8338). (C) Overlay of both results, where the difference between the fuzzy medians (Δm = 0.0342) quantifies the reduction in model uncertainty achieved by the proposed algorithm.**
(TIF)

S7 Fig The subplots compare the outputs of the proposed and traditional algorithms, following the same schema as S3 Fig. **(A) Result from the proposed precise algorithm (red solid line) with its fuzzy median (m**_**TX3e**_ **= 0.7555). (B) Result from the traditional approximate method (blue dashed line) with its fuzzy median (m**_**TX3e’**_ **= 0.7043). (C) Overlay of both results, where the difference between the fuzzy medians (Δm = 0.0512) quantifies the reduction in model uncertainty achieved by the proposed algorithm.**
(TIF)

S8 Fig The subplots compare the outputs of the proposed and traditional algorithms, following the same schema as S3 Fig. **(A) Result from the proposed precise algorithm (red solid line) with its fuzzy median (m**_**TX4e**_ **= 0.8500). (B) Result from the traditional approximate method (blue dashed line) with its fuzzy median (m**_**TX4e’**_ **= 0.8207). (C) Overlay of both results, where the difference between the fuzzy medians (Δm = 0.0293) quantifies the reduction in model uncertainty achieved by the proposed algorithm.**
(TIF)

S9 Fig The subplots compare the outputs of the proposed and traditional algorithms, following the same schema as S3 Fig. **(A) Result from the proposed precise algorithm (red solid line) with its fuzzy median (m**_**TX5e**_ **= 0.8425). (B) Result from the traditional approximate method (blue dashed line) with its fuzzy median (m**_**TX5e’**_ **= 0.8154). (C) Overlay of both results, where the difference between the fuzzy medians (Δm = 0.0271) quantifies the reduction in model uncertainty achieved by the proposed algorithm.**
(TIF)

S10 Fig The subplots compare the outputs of the proposed and traditional algorithms, following the same schema as S3 Fig. **(A) Result from the proposed precise algorithm (red solid line) with its fuzzy median (m**_**TX6e**_ **= 0.8681). (B) Result from the traditional approximate method (blue dashed line) with its fuzzy median (m**_**TX6e’**_ **= 0.8338). (C) Overlay of both results, where the difference between the fuzzy medians (Δm = 0.0343) quantifies the reduction in model uncertainty achieved by the proposed algorithm.**
(TIF)

S11 Fig The subplots compare the outputs of the proposed and traditional algorithms, following the same schema as S3 Fig. (A) Result from the proposed precise algorithm (red solid line) with its fuzzy median (m_TX7e_ = 0.8670). **(B) Result from the traditional approximate method (blue dashed line) with its fuzzy median (m**_**TX7e’**_ **= 0.8331). (C) Overlay of both results, where the difference between the fuzzy medians (Δm = 0.0339) quantifies the reduction in model uncertainty achieved by the proposed algorithm.**
(TIF)

S12 Fig The subplots compare the outputs of the proposed and traditional algorithms, following the same schema as S3 Fig. **(A) Result from the proposed precise algorithm (red solid line) with its fuzzy median (m**_**TX8e**_** = 0.8388). (B) Result from the traditional approximate method (blue dashed line) with its fuzzy median (m**_**TX8e’**_** = 0.8095). (C) Overlay of both results, where the difference between the fuzzy medians (Δm = 0.0293) quantifies the reduction in model uncertainty achieved by the proposed algorithm.** The most comprehensive comparison is presented in S13 Fig. It demonstrates that the precise computational results from our method are in near-perfect agreement (99.40% consistency) with the precise results from the benchmark study. Conversely, the approximate results from both methods deviate significantly from their respective precise curves.
(TIF)

S13 Fig The top event probabilities calculated by four methods are compared: the traditional normal fuzzy approximation from the literature (orange solid line), the precise normal fuzzy calculation from the literature (green solid line), the proposed precise trapezoidal fuzzy calculation (blue dashed line), and the traditional trapezoidal fuzzy approximation (black dashed line). **The results demonstrate that the proposed precise trapezoidal fuzzy calculation (blue dashed) and the literature’s precise normal fuzzy calculation (green solid) yield nearly identical results across all fault tree types, following an identical trend. This close agreement validates the reliability and accuracy of the proposed precise computational algorithm.** This divergence is not a discrepancy but a validation of our core thesis: model uncertainty introduced by approximation is a systematic error. The high agreement between the two precise methods confirms the correctness of our algorithm, while the larger uncertainty gap (blue shaded region) in the benchmark study underscores the superior ability of our method to mitigate this error. Therefore, the proposed algorithm not only validates successfully against a known benchmark but also provides a demonstrably more reliable and informative output for risk assessment.
(TIF)

S14 Fig.
**(A-F) Membership functions for the top event probability as the number of basic events increases from 4 to 9. Each subplot compares the results of the proposed precise algorithm (red solid line with fuzzy median m**_**TTe**_**) and the traditional approximate method (blue dashed line with fuzzy median m**_**TTe’**_**). The consistent leftward shift of the proposed algorithm’s fuzzy median demonstrates its superior precision. (G) Bar chart quantifying the relative model uncertainty reduction achieved by the proposed algorithm, which increases progressively from 1.20% to 7.49% as the system scales from 4 to 9 events, confirming the algorithm’s enhanced effectiveness in larger, more complex systems.**This demonstrates a critical aspect of robustness: graceful scaling. The algorithm consistently outperforms the traditional method, and the benefit of using the precise method becomes more pronounced for larger, more complex systems. This predictable enhancement—where increased complexity leads to greater relative accuracy—provides engineers with high confidence when applying the algorithm to real-world systems of unknown or variable scale. The algorithm effectively controls uncertainty propagation, ensuring reliable risk assessment even as the system model grows in complexity.
(TIF)

S15 FigThe fault tree structure is $\;T=\left(E_{1}+E_{2}+E_{3}+E_{4}+E_{5}+E_{6}+E_{7}+E_{8}+E_{9}\right)*E_{𝐨𝐫0}\;$ where the fuzzy median of $\;E_{𝐨𝐫0}$ varies.
**(A–F) Membership functions of the top event probability as the fuzzy median of the AND event increases from 0.03 to 0.08. Each subplot compares the proposed precise algorithm (red solid line, fuzzy median m**_**TTe**_ **) and the traditional method (blue dashed line, fuzzy median m**_**TTe’**_ **). The proposed algorithm consistently yields a lower fuzzy median, reflecting reduced uncertainty. (G) Bubble plot showing the relative uncertainty reduction (bubble size) achieved by the proposed algorithm. As the fuzzy median of the AND event increases, the reduction in model uncertainty decreases consistently (from 10.60% to 5.92%), demonstrating the algorithm’s adaptive robustness across varying input probabilities.**
(TIF)

S16 FigThe fault tree structure is $\;T=\left(E_{1}+E_{2}+E_{3}+E_{4}+E_{5}+E_{6}+E_{7}+E_{8}+E_{9}\right)*E_{𝐨𝐫0}\;$ where the fuzzy median of $\;E_{𝐨𝐫0}$ varies.**(A–F) Membership functions of the top event probability as the fuzzy median of the AND event increases from 0.003 to 0.008. Each subplot compares the proposed precise algorithm (red solid line, fuzzy median m**_**TTe**_**) and the traditional method (blue dashed line, fuzzy median m**_**TTe’**_**). The proposed algorithm consistently yields a lower fuzzy median, reflecting reduced uncertainty. (G) Step chart illustrating the relative uncertainty reduction achieved by the proposed algorithm. As the fuzzy median of the AND event increases, the reduction in model uncertainty decreases consistently (from 9.45% to 5.92%), demonstrating the algorithm’s robust performance even for low-probability events.** Critically, the data reveals that the algorithm’s advantage over the traditional method is preserved regardless of the input probability’s magnitude. The difference in results between the precise and traditional methods follows a consistent pattern, and the percentage of uncertainty reduction remains significant. This demonstrates that the algorithm is not overly sensitive to the specific values of input parameters. It performs reliably whether the additional event has a high or low probability, confirming its robust stability and suitability for assessing systems with diverse risk levels.(TIF)

S17 Fig This analysis extends the fault tree structure from S16 Fig by systematically adding AND events (from 1 to 5) with varying fuzzy medians. **(A) Line plot showing relative uncertainty reduction as the number of AND events increases. Three distinct fuzzy median values (0.2–blue line, 0.3–red line, 0.4–yellow line) demonstrate consistent decreasing trends in uncertainty reduction with additional AND events. All lines maintain consistent ordering (yellow highest, red intermediate, blue lowest) without intersection, confirming predictable behavior across parameter variations. (B) Bar chart comparing uncertainty reduction for AND events with fuzzy medians of 0.5 (blue bars) and 0.6 (red bars). Both sequences show a characteristic pattern of initial increase followed by decrease as more AND events are added. (C) Bubble plot displaying uncertainty reduction for AND events with fuzzy medians of 0.7 (blue bubbles) and 0.8 (red bubbles). Bubble sizes increase consistently with additional AND events while maintaining rightward positional progression. Collectively, these results demonstrate the algorithm’s robust performance maintains predictable uncertainty reduction patterns across combined variations in system complexity (AND event count) and input parameters (fuzzy median values).** The most significant conclusion from this comprehensive analysis is the narrow range of performance variation. As the system scales up in complexity and the input parameters vary widely, the model uncertainty reduction effect remains tightly bounded between 9.23% and 11.33%. This indicates that the algorithm’s advantage is not contingent on a specific system configuration or input value. It delivers reliable, high-quality results across a broad spectrum of conditions.
(TIF)

S1 Table E4 perturbation test set (perturbation level 5%). (PDF)

S2 Table E4 perturbation test set (perturbation level 10%). (PDF)

S3 Table E4 perturbation test set (perturbation level 15%). (PDF)

S4 Table E5 perturbation test set (perturbation level 5%). (PDF)

S5 Table E5 perturbation test set (perturbation level 10%). (PDF)

S6 Table E5 perturbation test set (perturbation level 15%). (PDF)

S7 Table E7 perturbation test set (perturbation level 5%). (PDF)

S8 Table E7 perturbation test set (perturbation level 10%). (PDF)

S9 Table E7 perturbation test set (perturbation level 15%). (PDF)

S1 Appendix Monotonicity Proof for AND-Gate Systems(Left). (PDF)

S2 Appendix Monotonicity Proof for AND-Gate Systems (Right). (PDF)

S3 Appendix Monotonicity Proof for OR-Gate Systems(Left). (PDF)

S4 Appendix Monotonicity Proof for OR-Gate Systems (Right). (PDF)

S5 Appendix Monotonicity Proof for AND OR Gates (Left). (PDF)

S6 Appendix Monotonicity Proof for AND OR Gates (Right). (PDF)

## References

[1] Cacace J, Caccavale R, Finzi A, Grieco R. Combining human guidance and structured task execution during physical human–robot collaboration. J Intell Manuf. 2022;34(7):3053–67. doi: 10.1007/s10845-022-01989-y

[2] Li P, Wang Y, Yang Z. Risk assessment of maritime autonomous surface ships collisions using an FTA-FBN model. Ocean Eng. 2024;309:118444. doi: 10.1016/j.oceaneng.2024.118444

[3] Daas S, Innal F. Uncertainty assessment of improved multistate reliability in the sociotechnical systems based on the polymorphic fuzzy entropy fault tree analysis and triptych cost-benefit-safety analysis. J Loss Prev Process Ind. 2025;94:105570. doi: 10.1016/j.jlp.2025.105570

[4] Eltahan FM, Toderas M, Mansour MS, El-Ashtoukhy ESZ, Abdou MA, Shokry F. Applying a semi-quantitative risk assessment on petroleum production unit. Sci Rep. 2024;14(1):7603. doi: 10.1038/s41598-024-57600-2 38556541 PMC10982294

[5] Kuzu AC, Senol YE. Fault tree analysis of cargo leakage from manifold connection in fuzzy environment: A novel case of anhydrous ammonia. Ocean Eng. 2021;238:109720. doi: 10.1016/j.oceaneng.2021.109720

[6] He W, Yin X, Shao Y, Chen D, Mi J, Jiao Y. Fuzzy Fault Tree Maintenance Decision Analysis for Aviation Fuel Pumps Based on Nutcracker Optimization Algorithm–Graph Neural Network Improvement. Mathematics. 2024;13(1):123. doi: 10.3390/math13010123

[7] Wen J, Zhang L, Guo Z, Tang W, Shang S, Liu M, et al. Reliability and safety assessment of submarine pipeline stopper based on Fuzzy Comprehensive Dynamic Bayesian Network. Ocean Eng. 2024;298:117099. doi: 10.1016/j.oceaneng.2024.117099

[8] Guo X, Ji J, Khan F, Ding L, Tong Q. A novel fuzzy dynamic Bayesian network for dynamic risk assessment and uncertainty propagation quantification in uncertainty environment. Saf Sci. 2021;141:105285. doi: 10.1016/j.ssci.2021.105285

[9] Animah I. Application of bayesian network in the maritime industry: Comprehensive literature review. Ocean Eng. 2024;302:117610. doi: 10.1016/j.oceaneng.2024.117610

[10] Purba JH. A fuzzy-based reliability approach to evaluate basic events of fault tree analysis for nuclear power plant probabilistic safety assessment. Ann Nucl Energy. 2014;70:21–9. doi: 10.1016/j.anucene.2014.02.022

[11] Ferdous R, Khan F, Sadiq R, Amyotte P, Veitch B. Fault and event tree analyses for process systems risk analysis: uncertainty handling formulations. Risk Anal. 2011;31(1):86–107. doi: 10.1111/j.1539-6924.2010.01475.x 20731791

[12] Sadiq R, Saint-Martin E, Kleiner Y. Predicting risk of water quality failures in distribution networks under uncertainties using fault-tree analysis. Urban Water J. 2008;5(4):287–304. doi: 10.1080/15730620802213504

[13] Bjerga T, Aven T, Zio E. An illustration of the use of an approach for treating model uncertainties in risk assessment. Reliab Eng Syst Saf. 2014;125:46–53. doi: 10.1016/j.ress.2014.01.014

[14] Suresh PV, Babar AK, Raj VV. Uncertainty in fault tree analysis: A fuzzy approach. Fuzzy Sets Syst. 1996;83(2):135–41. doi: 10.1016/0165-0114(95)00386-x

[15] Yazdi M, Kabir S, Walker M. Uncertainty handling in fault tree based risk assessment: State of the art and future perspectives. Process Saf Environ Prot. 2019;131:89–104. doi: 10.1016/j.psep.2019.09.003

[16] Liu P, Yang L, Gao Z, Li S, Gao Y. Fault tree analysis combined with quantitative analysis for high-speed railway accidents. Saf Sci. 2015;79:344–57. doi: 10.1016/j.ssci.2015.06.017

[17] Kaushik M, Kumar M. An α-cut interval based IF-importance measure for intuitionistic fuzzy fault tree analysis of subsea oil and gas production system. Appl Ocean Res. 2022;125:103229. doi: 10.1016/j.apor.2022.103229

[18] Jianxing Y, Haicheng C, Yang Y, Zhenglong Y. A weakest t-norm based fuzzy fault tree approach for leakage risk assessment of submarine pipeline. J Loss Prev Process Ind. 2019;62:103968. doi: 10.1016/j.jlp.2019.103968

[19] Komal. Novel approach to analyse vague reliability of repairable industrial systems. Comput Ind Eng. 2022;169:108199. doi: 10.1016/j.cie.2022.108199

[20] Badida P, Balasubramaniam Y, Jayaprakash J. Risk evaluation of oil and natural gas pipelines due to natural hazards using fuzzy fault tree analysis. J Nat Gas Sci Eng. 2019;66:284–92. doi: 10.1016/j.jngse.2019.04.010

[21] Lu M, Jin Y, Lin J, Liu Q, Du Y, Yang Y. Fuzzy fault tree analysis of EVAC system based on improved SAM- FFTA with butterfly optimization algorithm. Eng Fail Anal. 2023;154:107658. doi: 10.1016/j.engfailanal.2023.107658

[22] Zhang C, Yu C, Yuan L, Balezentis T, Zeng S. Assessment of Conductivity-Temperature-Depth via multi-criteria approach: Regret theory based model on the pythagorean fuzzy environment. Ocean Eng. 2022;266:112740. doi: 10.1016/j.oceaneng.2022.112740

[23] Sun H, Yang Z, Wang L, Xie J. Leakage failure probability assessment of submarine pipelines using a novel pythagorean fuzzy bayesian network methodology. Ocean Eng. 2023;288:115954. doi: 10.1016/j.oceaneng.2023.115954

[24] Kaushik M, Kumar M. An integrated approach of intuitionistic fuzzy fault tree and Bayesian network analysis applicable to risk analysis of ship mooring operations. Ocean Eng. 2023;269:113411. doi: 10.1016/j.oceaneng.2022.113411

[25] Tan J, Chen X, Bu Y, Wang F, Wang J, Huang X, et al. Incorporating FFTA based safety assessment of lithium-ion battery energy storage systems in multi-objective optimization for integrated energy systems. Appl Energy. 2024;367:123472. doi: 10.1016/j.apenergy.2024.123472

[26] Ding R, Liu Z, Xu J, Meng F, Sui Y, Men X. A novel approach for reliability assessment of residual heat removal system for HPR1000 based on failure mode and effect analysis, fault tree analysis, and fuzzy Bayesian network methods. Reliab Eng Syst Saf. 2021;216:107911. doi: 10.1016/j.ress.2021.107911

[27] Bi Y, Wang S, Zhang C, Cong H, Qu B, Li J, et al. Safety and reliability analysis of the solid propellant casting molding process based on FFTA and PSO-BPNN. Process Saf Environ Prot. 2022;164:528–38. doi: 10.1016/j.psep.2022.06.032

[28] Sakar C, Sokukcu M. Dynamic analysis of pilot transfer accidents. Ocean Eng. 2023;287:115823. doi: 10.1016/j.oceaneng.2023.115823

[29] Meng X, Zhu J, Chen G, Shi J, Li T, Song G. Dynamic and quantitative risk assessment under uncertainty during deepwater managed pressure drilling. J Clean Prod. 2022;334:130249. doi: 10.1016/j.jclepro.2021.130249

[30] Ahmed S, Li T, Huang S, Cao J. Dynamic and quantitative risk assessment of Cruise ship pod propulsion system failure: An integrated Type-2 Fuzzy-Bayesian approach. Ocean Eng. 2023;279:114601. doi: 10.1016/j.oceaneng.2023.114601

[31] Singh K, Kaushik M, Kumar M. Integrating α-cut interval based fuzzy fault tree analysis with Bayesian network for criticality analysis of submarine pipeline leakage: A novel approach. Process Saf Environ Prot. 2022;166:189–201. doi: 10.1016/j.psep.2022.07.058

[32] Djemai Z, Aissani N, Bekrar A, Lounis Z. Risk analysis of petroleum storage tank based on uncertain data incorporated into mapped Bow-tie to Bayesian network. Process Saf Environ Prot. 2024;190:1202–21. doi: 10.1016/j.psep.2024.07.114

[33] Sarıalioğlu S, Uğurlu Ö, Aydın M, Vardar B, Wang J. A hybrid model for human-factor analysis of engine-room fires on ships: HFACS-PV&FFTA. Ocean Eng. 2020;217:107992. doi: 10.1016/j.oceaneng.2020.107992

[34] Sezer SI, Camliyurt G, Aydin M, Akyuz E, Gardoni P. A bow-tie extended D-S evidence-HEART modelling for risk analysis of cargo tank cracks on oil/chemical tanker. Reliab Eng Syst Safe. 2023;237:109346. doi: 10.1016/j.ress.2023.109346

[35] Huang W, Liu Y, Zhang Y, Zhang R, Xu M, De Dieu GJ, et al. Fault Tree and Fuzzy D-S Evidential Reasoning combined approach: An application in railway dangerous goods transportation system accident analysis. Inf Sci. 2020;520:117–29. doi: 10.1016/j.ins.2019.12.089

[36] Erdem P, Akyuz E. An interval type-2 fuzzy SLIM approach to predict human error in maritime transportation. Ocean Eng. 2021;232:109161. doi: 10.1016/j.oceaneng.2021.109161

[37] Celik M, Lavasani SM, Wang J. A risk-based modelling approach to enhance shipping accident investigation. Saf Sci. 2010;48(1):18–27. doi: 10.1016/j.ssci.2009.04.007

[38] Gürgen S, Yazır D, Konur O. Fuzzy fault tree analysis for loss of ship steering ability. Ocean Eng. 2023;279:114419. doi: 10.1016/j.oceaneng.2023.114419

[39] Qiao D, Zhou X, Ye X, Tang G, Lu L, Ou J. Security risk assessment of submerged floating tunnel based on fault tree and multistate fuzzy Bayesian network. Ocean Coast Manag. 2024;258:107355. doi: 10.1016/j.ocecoaman.2024.107355

[40] Sokukcu M, Sakar C. Risk analysis of collision accidents during underway STS berthing maneuver through integrating fault tree analysis (FTA) into Bayesian network (BN). Appl Ocean Res. 2022;126:103290. doi: 10.1016/j.apor.2022.103290

[41] Onisawa T. An approach to human reliability in man-machine systems using error possibility. Fuzzy Sets Syst. 1988;27(2):87–103. doi: 10.1016/0165-0114(88)90140-6

[42] Kabir S, Walker M, Papadopoulos Y, Rüde E, Securius P. Fuzzy temporal fault tree analysis of dynamic systems. Int J Approx Reason. 2016;77:20–37. doi: 10.1016/j.ijar.2016.05.006

[43] Lutfi Tunçel A, Bal Beşikçi E, Akyuz E, Arslan O. Safety analysis of fire and explosion (F&E) accidents risk in bulk carrier ships under fuzzy fault tree approach. Saf Sci. 2023;158:105972. doi: 10.1016/j.ssci.2022.105972

[44] Wang Z, Wang J, Guan P, Song W. SIL assessment of in-service safety instrumented systems in the chemical industry based on FBN-LOPA. Process Saf Environ Prot. 2025;195:106740. doi: 10.1016/j.psep.2024.12.121

[45] Zhang J, Kang J, Sun L, Bai X. Risk assessment of floating offshore wind turbines based on fuzzy fault tree analysis. Ocean Eng. 2021;239:109859. doi: 10.1016/j.oceaneng.2021.109859

[46] Yazdi M, Zarei E. Uncertainty Handling in the Safety Risk Analysis: An Integrated Approach Based on Fuzzy Fault Tree Analysis. J Fail Anal Preven. 2018;18(2):392–404. doi: 10.1007/s11668-018-0421-9

[47] Guo X, Ji J, Khan F, Ding L, Yang Y. Fuzzy Bayesian network based on an improved similarity aggregation method for risk assessment of storage tank accident. Process Saf Environ Prot. 2021;149:817–30. doi: 10.1016/j.psep.2021.03.017

[48] Brunelli M, Mezei J. An inquiry into approximate operations on fuzzy numbers. Int J Approx Reason. 2017;81:147–59. doi: 10.1016/j.ijar.2016.11.011

[49] Diao X, Jiang J, Mebarki A, Ni L, Duo Y, Chen S, et al. Risk analysis of domino effect of leakage accident of petrochemical pipeline based on analytic hierarchy process and fuzzy fault tree analysis. Saf Sci. 2025;187:106852. doi: 10.1016/j.ssci.2025.106852

[50] Zadeh LA. Fuzzy sets. Inf Control. 1965;8(3):338–53. doi: 10.1016/s0019-9958(65)90241-x

[51] Giachetti RE, Young RE. A parametric representation of fuzzy numbers and their arithmetic operators. Fuzzy Sets Syst. 1997;91(2):185–202. doi: 10.1016/s0165-0114(97)00140-1

[52] Zadeh LA. Fuzzy sets as a basis for a theory of possibility. Fuzzy Sets Syst. 1978;1(1):3–28. doi: 10.1016/0165-0114(78)90029-5

[53] Zadeh LA. Probability measures of fuzzy events. J Math Anal Appl. 1968;23:421–7.

[54] Klinke A, Renn O. A new approach to risk evaluation and management: risk-based, precaution-based, and discourse-based strategies. Risk Anal. 2002;22(6):1071–94. doi: 10.1111/1539-6924.00274 12530780

[55] Zhang Z, Guo Y, Wang D, Li G, Peng D. A Noisy-OR gate based fuzzy fault tree approach for micro-leakage evaluation of bolt-gasket-flange connection (BGFC). J Loss Prev Process Ind. 2021;71:104521. doi: 10.1016/j.jlp.2021.104521

[56] Garg A, Maiti J, Kumar A. Granulized Z‐OWA aggregation operator and its application in fuzzy risk assessment. Int J of Intelligent Sys. 2021;37(2):1479–508. doi: 10.1002/int.22682

[57] Aydin M, Akyuz E, Turan O, Arslan O. Validation of risk analysis for ship collision in narrow waters by using fuzzy Bayesian networks approach. Ocean Eng. 2021;231:108973. doi: 10.1016/j.oceaneng.2021.108973

[58] Shi S, Jiang B, Meng X. Assessment of gas and dust explosion in coal mines by means of fuzzy fault tree analysis. Int J Min Sci Technol. 2018;28(6):991–8. doi: 10.1016/j.ijmst.2018.07.007

[59] Kumar M, Kaushik M. System failure probability evaluation using fault tree analysis and expert opinions in intuitionistic fuzzy environment. J Loss Prev Process Ind. 2020;67:104236. doi: 10.1016/j.jlp.2020.104236

[60] Zhang Y, Zhao L. A Novel Underlying Algorithm for Reducing Uncertainty in Process Industry Risk Assessment. Processes. 2024;12(7):1292. doi: 10.3390/pr12071292

[61] Dubois D, Prade H. Systems of linear fuzzy constraints. Fuzzy Sets Syst. 1980;3(1):37–48. doi: 10.1016/0165-0114(80)90004-4

[62] OREDA. Offshore Reliability Data Handbook. 4th edition. Trondheim, Norway: DNV; 2002, 568.

