# Supplementary figures and images for: A novel algorithm for model uncertainty reduction in trapezoidal fuzzy fault tree risk assessment
Source: PLoS One. 2025 Dec 15;20(12):e0335759. doi: 10.1371/journal.pone.0335759 (PMC12704870; doi:10.1371/journal.pone.0335759)

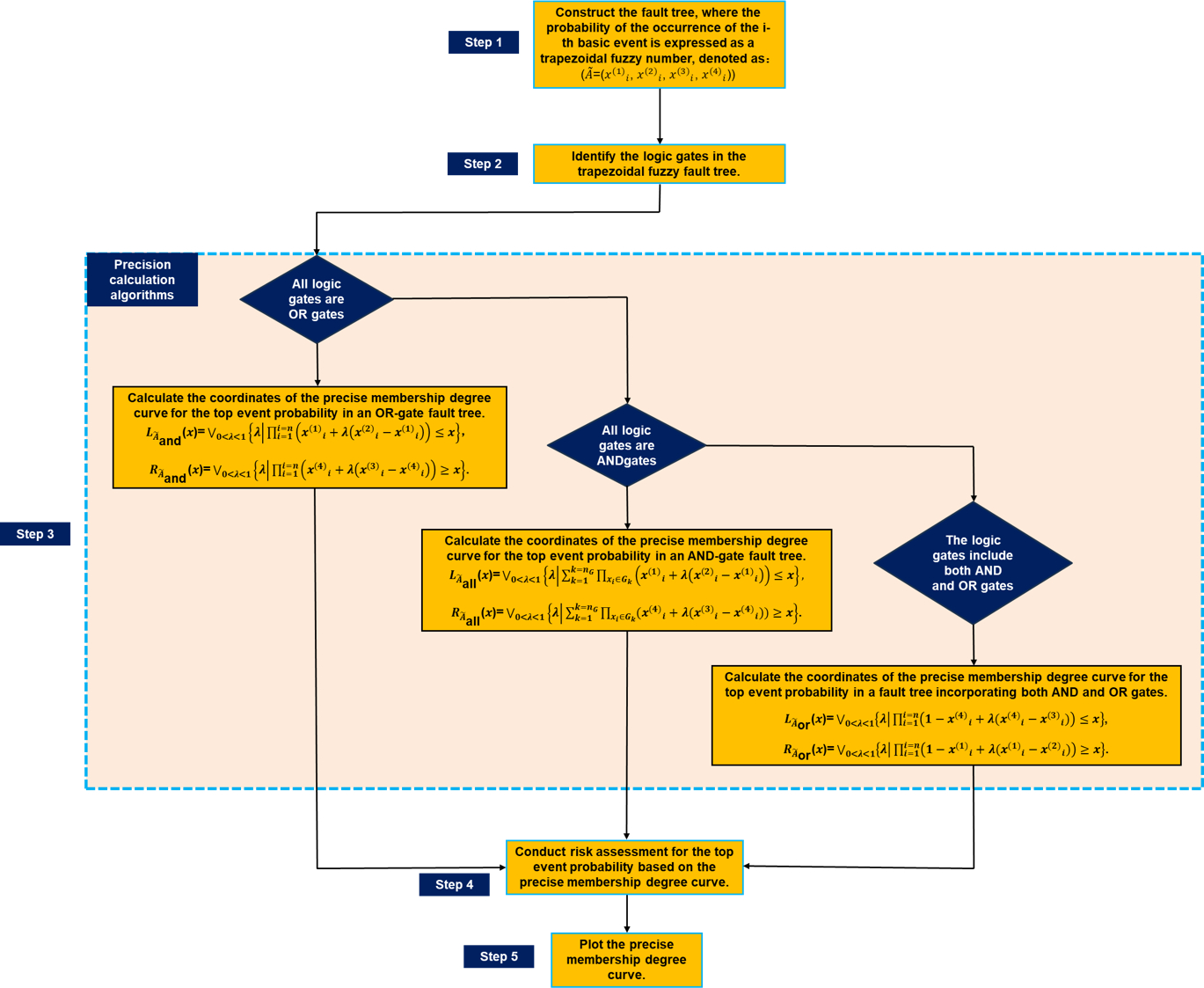

Supplement: S1 Fig — The process continues with (4) Membership Function Curve Plotting and concludes with (5) Risk Assessment. (TIF) [file pone.0335759.s001.tif]

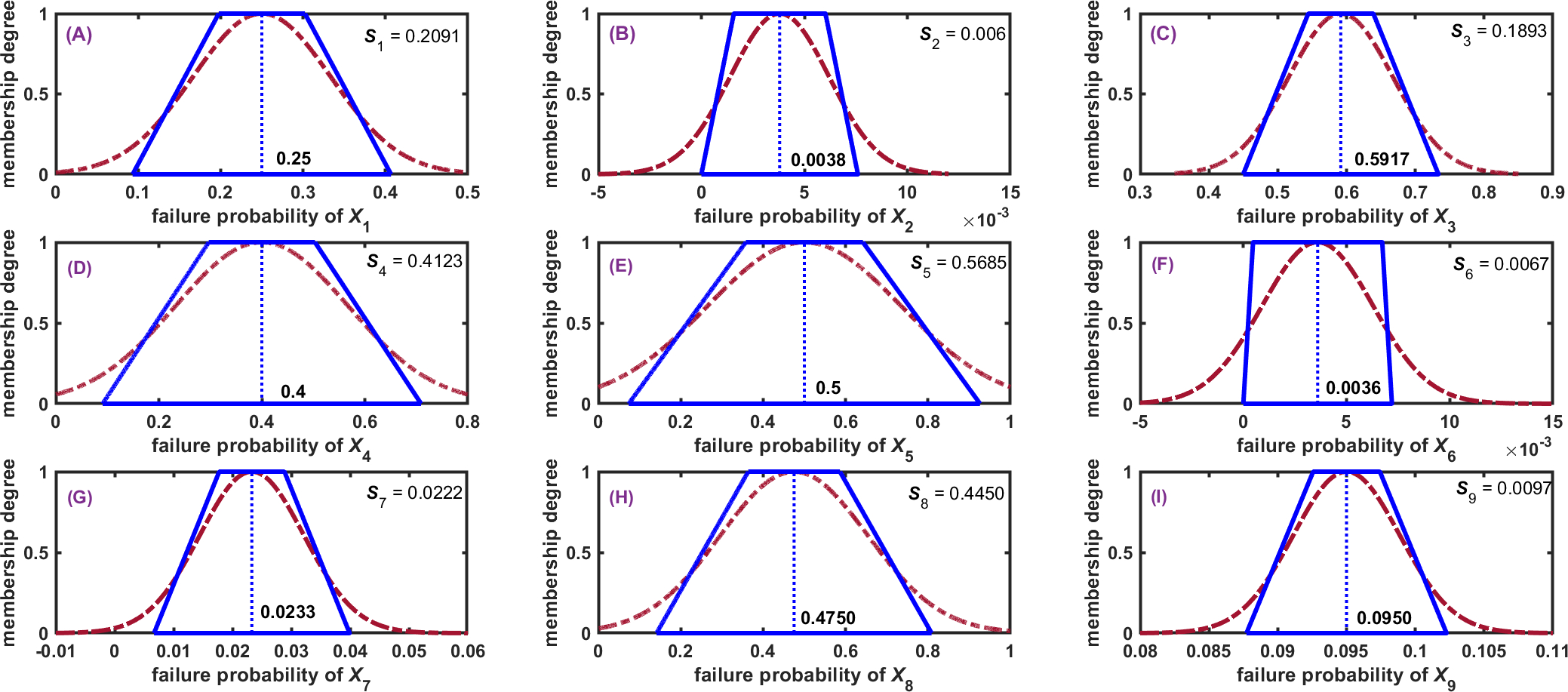

Supplement: S2 Fig — In each subplot, the original normal fuzzy number is shown as a red dashed line, while the converted trapezoidal fuzzy number is represented by a solid blue line. A blue dashed vertical line indicates the shared fuzzy median value for both distributions in each case. The conversion is based on the principle of equivalent area and identical fuzzy median between the original and converted forms, with the area (S) and median value for each basic event provided in the Table 1. (TIF) [file pone.0335759.s002.tif]

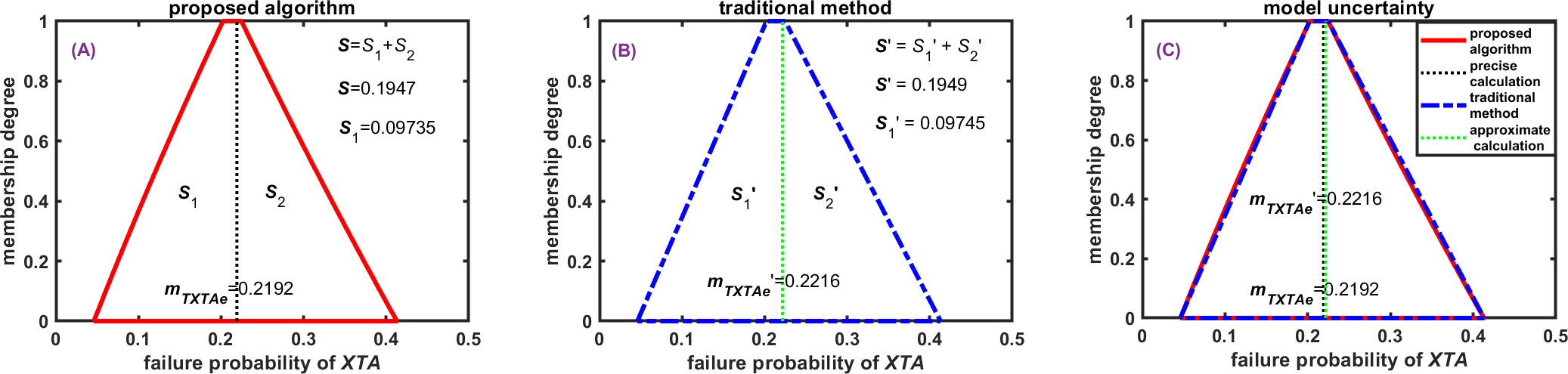

Supplement: S3 Fig. — (A) Result from the proposed precise algorithm, showing the membership function as a red solid line. The fuzzy median (m TXTAe ) is indicated by the intersection of the black dashed line (area bisector) with the x-axis. (B) Result from the traditional approximate method, showing the distorted membership function as a blue dashed line and its fuzzy median (m TXTAe’ ) via a green dashed area bisector. (C) Overlay of (A) and (B), demonstrating the reduction in model uncertainty. The distance between the black dashed line and the green dashed line (m TXTAe = 0.2192 vs m TXTAe’ = 0.2216) represents the absolute value of the reduction in model uncertainty. (TIF) [file pone.0335759.s003.tif]

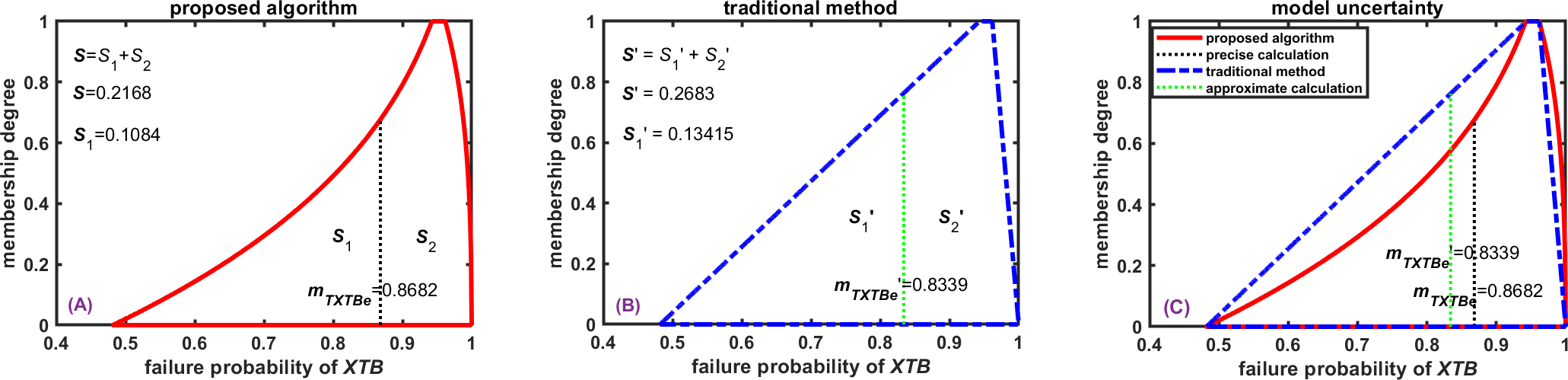

Supplement: S4 Fig — (A) Result from the proposed precise algorithm (red solid line) with its fuzzy median (m TXTBe = 0.8682). (B) Result from the traditional approximate method (blue dashed line) with its fuzzy median (m TXTBe’ = 0.8339). (C) Overlay of both results, where the difference between the fuzzy medians (Δm = 0.0343) quantifies the reduction in model uncertainty achieved by the proposed algorithm. (TIF) [file pone.0335759.s004.tif]

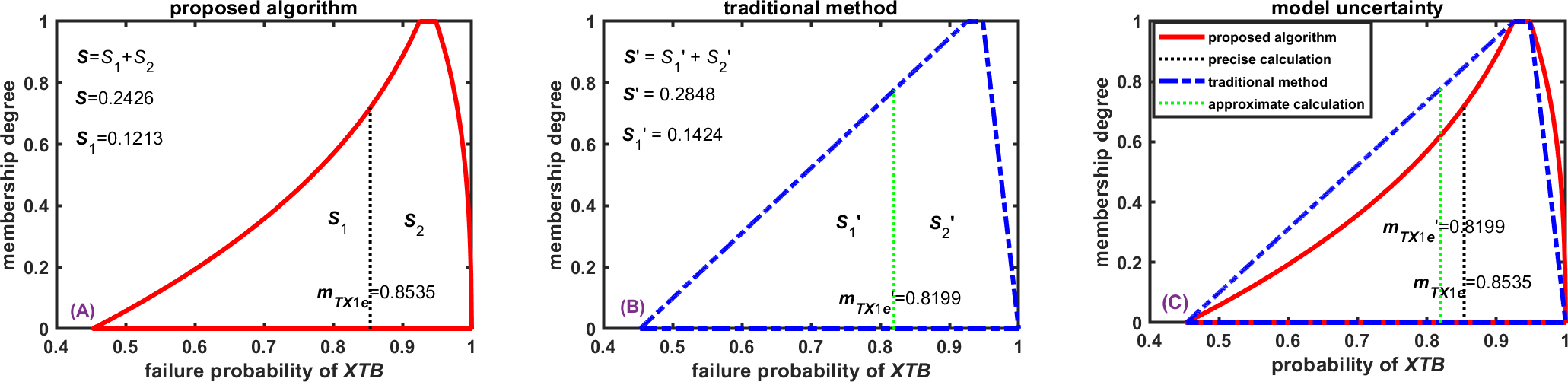

Supplement: S5 Fig — (A) Result from the proposed precise algorithm (red solid line) with its fuzzy median (m TX1e = 0.8535). (B) Result from the traditional approximate method (blue dashed line) with its fuzzy median (m TX1e’ = 0.8199). (C) Overlay of both results, where the difference between the fuzzy medians (Δm = 0.0336) quantifies the reduction in model uncertainty achieved by the proposed algorithm. (TIF) [file pone.0335759.s005.tif]

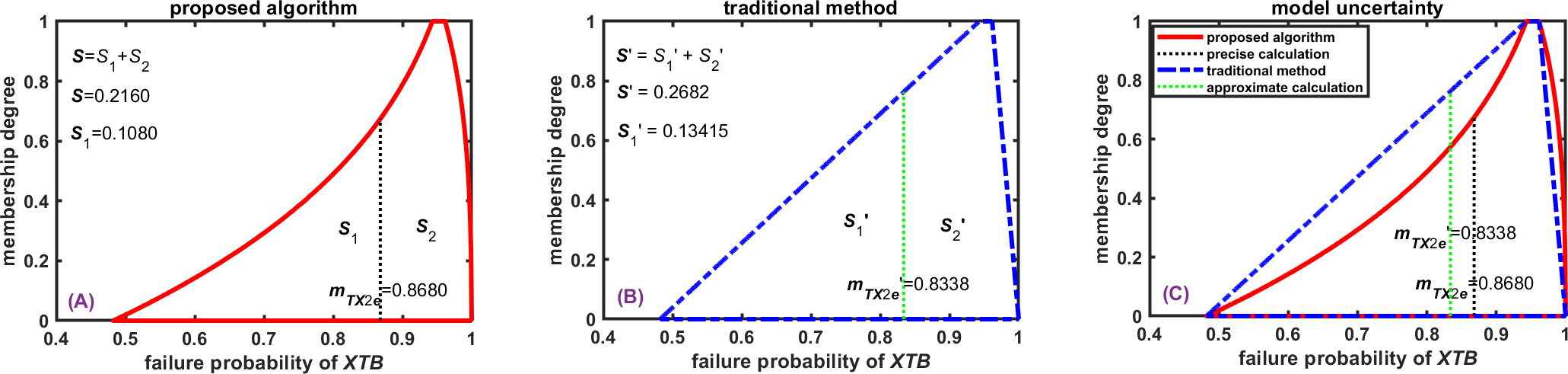

Supplement: S6 Fig — (A) Result from the proposed precise algorithm (red solid line) with its fuzzy median (m TX2e = 0.8680). (B) Result from the traditional approximate method (blue dashed line) with its fuzzy median (m TX2e’ = 0.8338). (C) Overlay of both results, where the difference between the fuzzy medians (Δm = 0.0342) quantifies the reduction in model uncertainty achieved by the proposed algorithm. (TIF) [file pone.0335759.s006.tif]

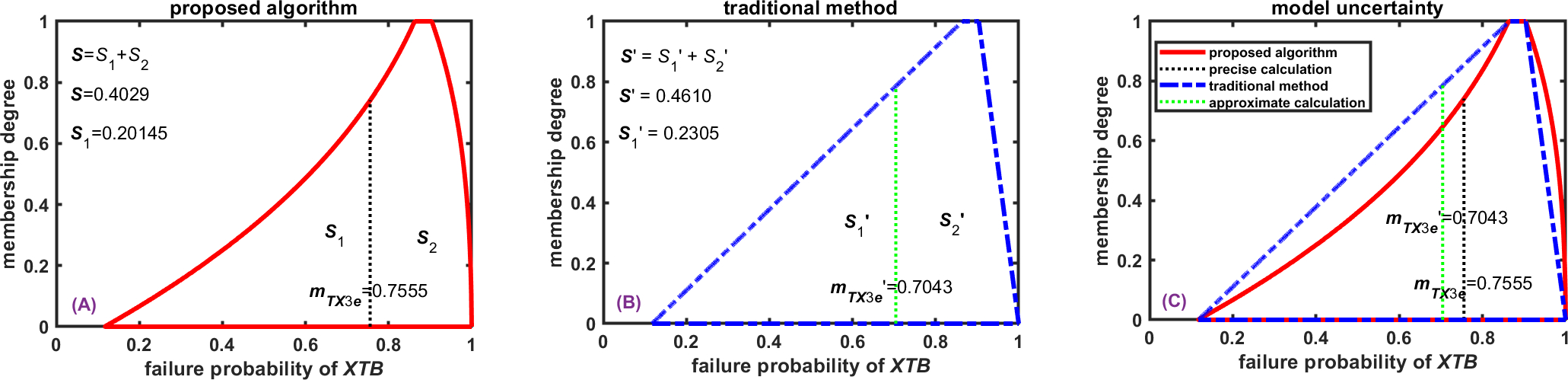

Supplement: S7 Fig — (A) Result from the proposed precise algorithm (red solid line) with its fuzzy median (m TX3e = 0.7555). (B) Result from the traditional approximate method (blue dashed line) with its fuzzy median (m TX3e’ = 0.7043). (C) Overlay of both results, where the difference between the fuzzy medians (Δm = 0.0512) quantifies the reduction in model uncertainty achieved by the proposed algorithm. (TIF) [file pone.0335759.s007.tif]

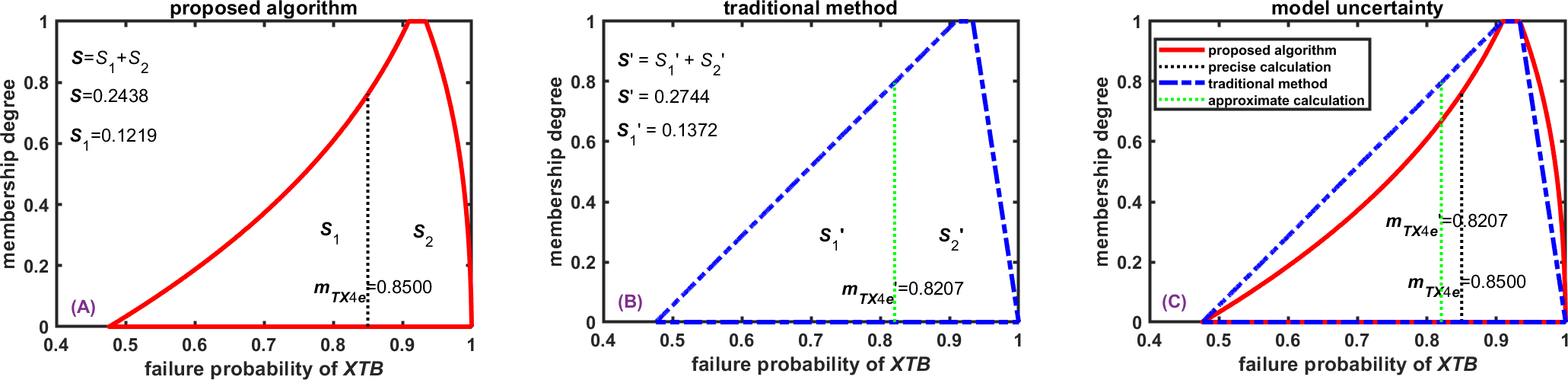

Supplement: S8 Fig — (A) Result from the proposed precise algorithm (red solid line) with its fuzzy median (m TX4e = 0.8500). (B) Result from the traditional approximate method (blue dashed line) with its fuzzy median (m TX4e’ = 0.8207). (C) Overlay of both results, where the difference between the fuzzy medians (Δm = 0.0293) quantifies the reduction in model uncertainty achieved by the proposed algorithm. (TIF) [file pone.0335759.s008.tif]

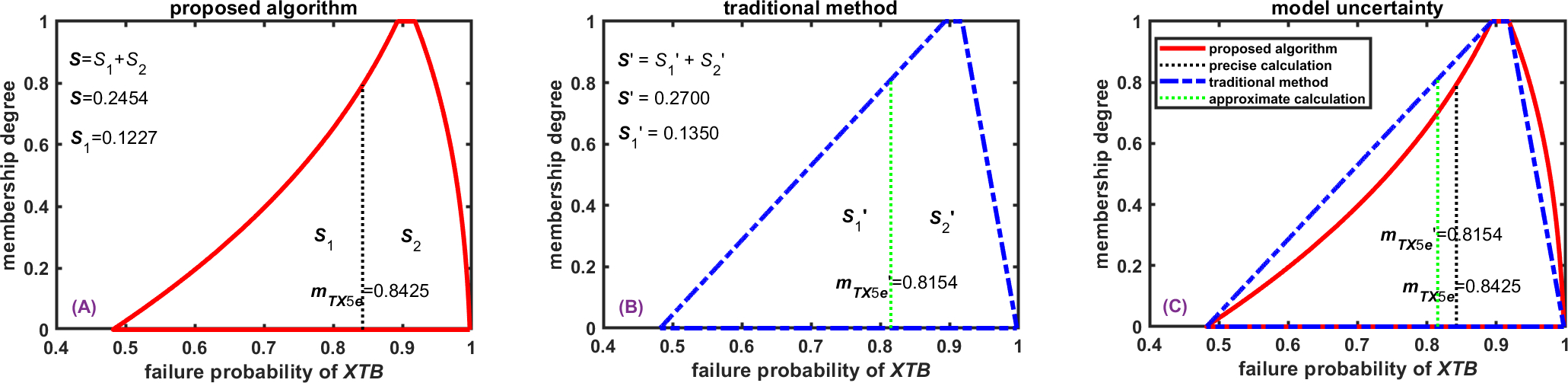

Supplement: S9 Fig — (A) Result from the proposed precise algorithm (red solid line) with its fuzzy median (m TX5e = 0.8425). (B) Result from the traditional approximate method (blue dashed line) with its fuzzy median (m TX5e’ = 0.8154). (C) Overlay of both results, where the difference between the fuzzy medians (Δm = 0.0271) quantifies the reduction in model uncertainty achieved by the proposed algorithm. (TIF) [file pone.0335759.s009.tif]

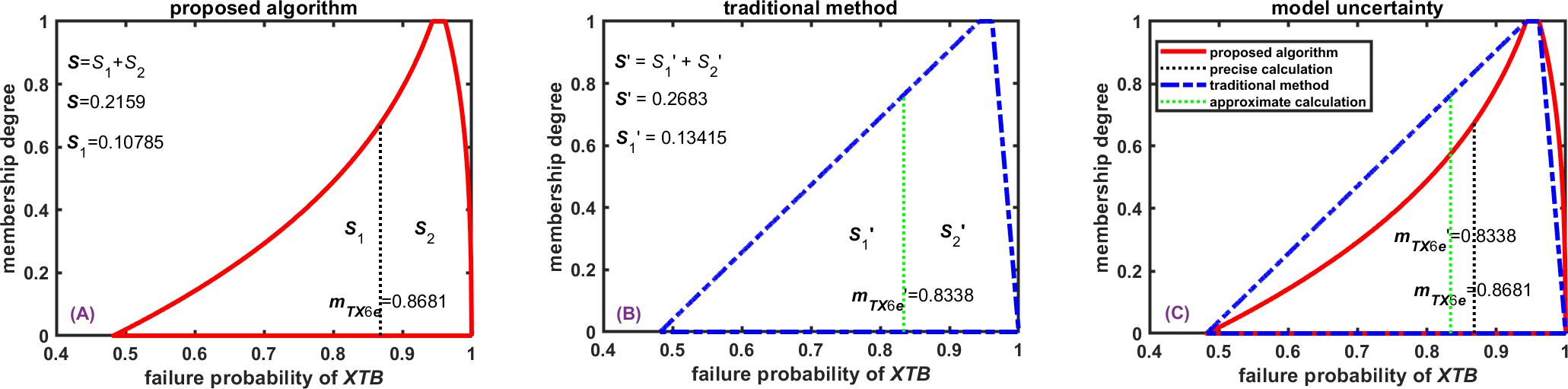

Supplement: S10 Fig — (A) Result from the proposed precise algorithm (red solid line) with its fuzzy median (m TX6e = 0.8681). (B) Result from the traditional approximate method (blue dashed line) with its fuzzy median (m TX6e’ = 0.8338). (C) Overlay of both results, where the difference between the fuzzy medians (Δm = 0.0343) quantifies the reduction in model uncertainty achieved by the proposed algorithm. (TIF) [file pone.0335759.s010.tif]

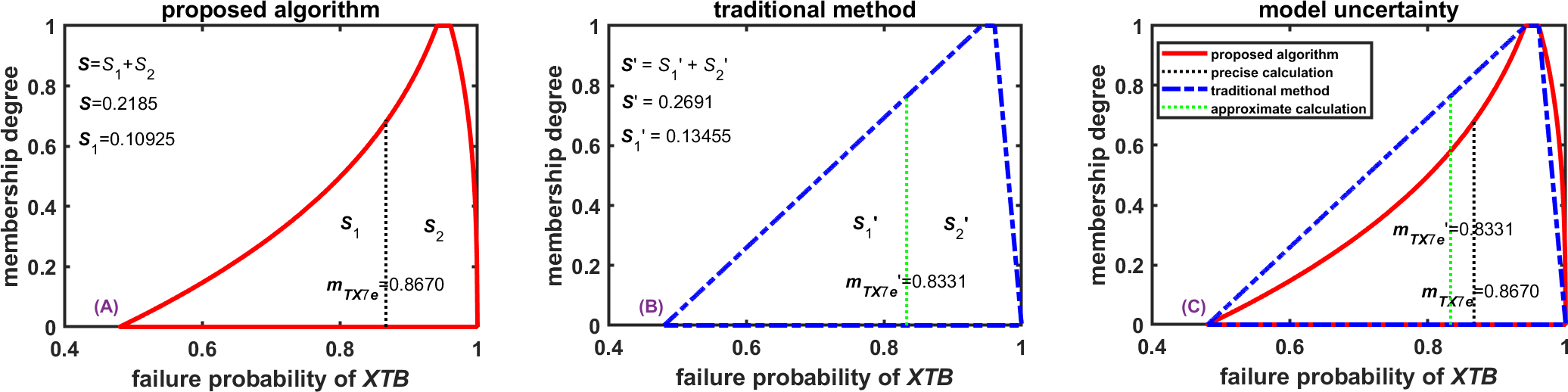

Supplement: S11 Fig — (B) Result from the traditional approximate method (blue dashed line) with its fuzzy median (m TX7e’ = 0.8331). (C) Overlay of both results, where the difference between the fuzzy medians (Δm = 0.0339) quantifies the reduction in model uncertainty achieved by the proposed algorithm. (TIF) [file pone.0335759.s011.tif]

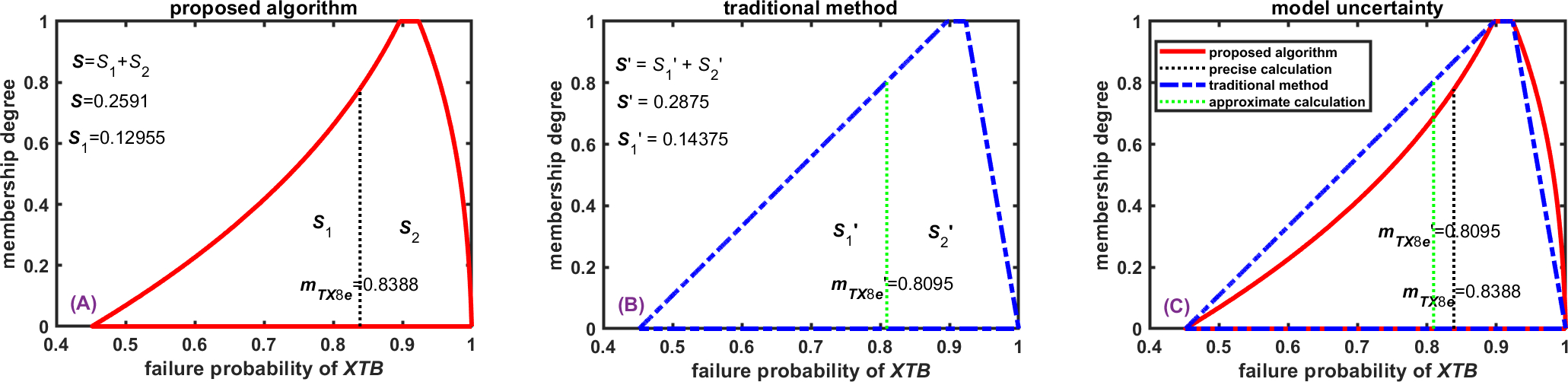

Supplement: S12 Fig — (A) Result from the proposed precise algorithm (red solid line) with its fuzzy median (mTX8e = 0.8388). (B) Result from the traditional approximate method (blue dashed line) with its fuzzy median (mTX8e’ = 0.8095). (C) Overlay of both results, where the difference between the fuzzy medians (Δm = 0.0293) quantifies the reduction in model uncertainty achieved by the proposed algorithm. The most comprehensive comparison is presented in S13 Fig. It demonstrates that the precise computational results from our method are in near-perfect agreement (99.40% consistency) with the precise results from the benchmark study. Conversely, the approximate results from both methods deviate significantly from their respective precise curves. (TIF) [file pone.0335759.s012.tif]

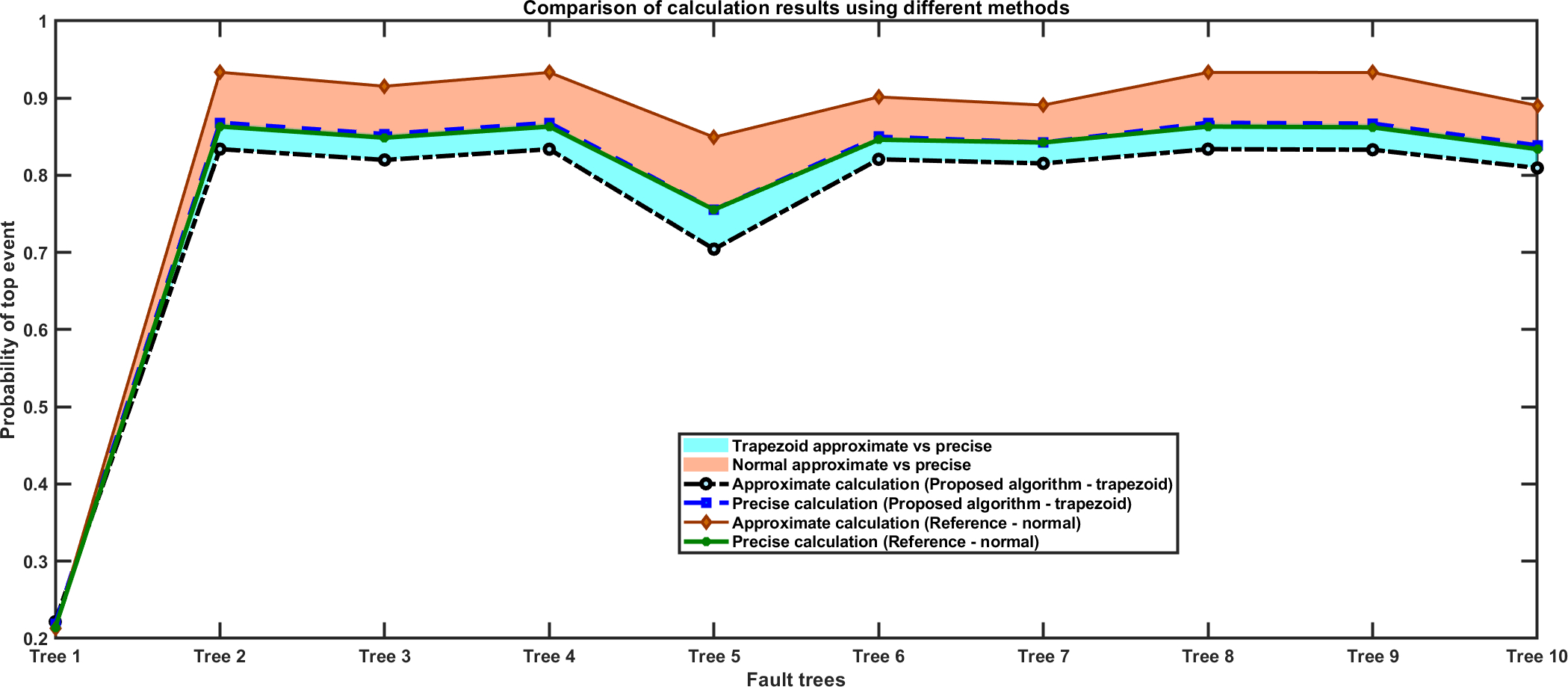

Supplement: S13 Fig — The results demonstrate that the proposed precise trapezoidal fuzzy calculation (blue dashed) and the literature’s precise normal fuzzy calculation (green solid) yield nearly identical results across all fault tree types, following an identical trend. This close agreement validates the reliability and accuracy of the proposed precise computational algorithm. This divergence is not a discrepancy but a validation of our core thesis: model uncertainty introduced by approximation is a systematic error. The high agreement between the two precise methods confirms the correctness of our algorithm, while the larger uncertainty gap (blue shaded region) in the benchmark study underscores the superior ability of our method to mitigate this error. Therefore, the proposed algorithm not only validates successfully against a known benchmark but also provides a demonstrably more reliable and informative output for risk assessment. (TIF) [file pone.0335759.s013.tif]

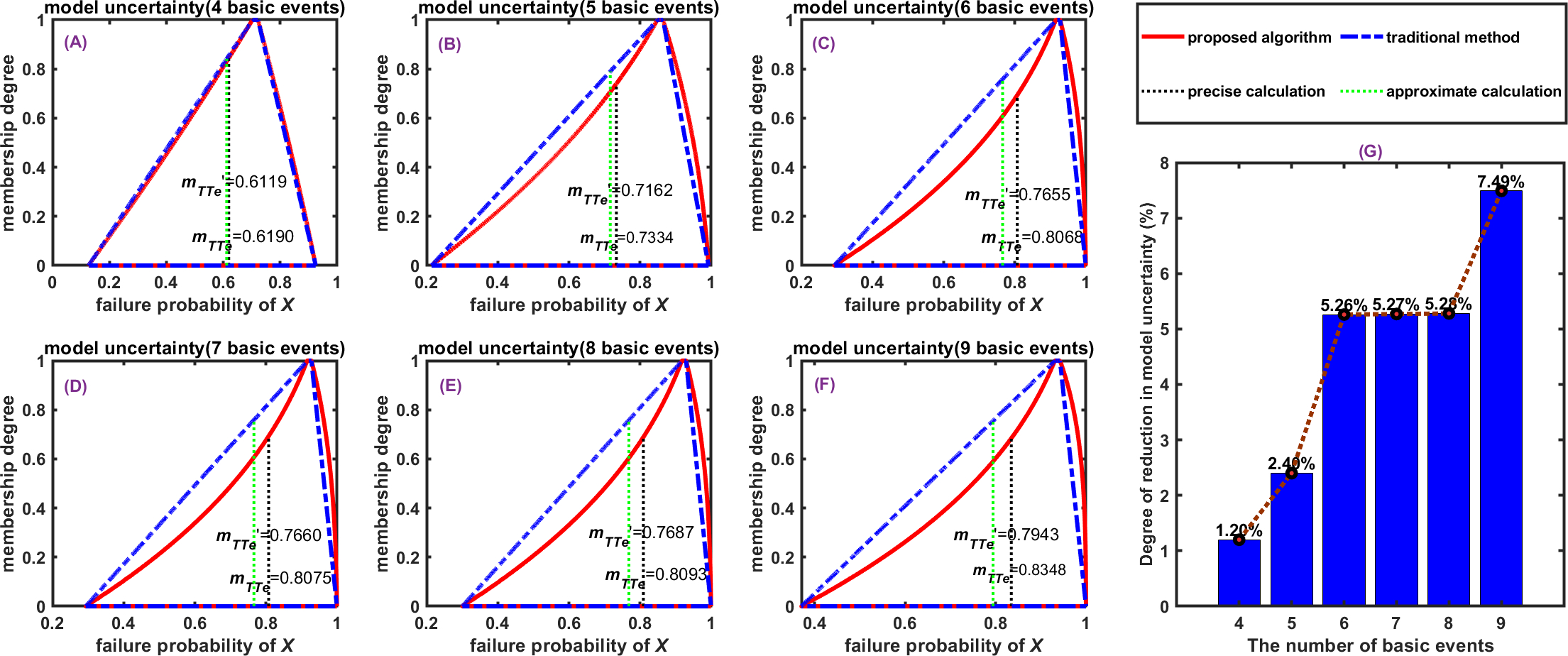

Supplement: S14 Fig. — (A-F) Membership functions for the top event probability as the number of basic events increases from 4 to 9. Each subplot compares the results of the proposed precise algorithm (red solid line with fuzzy median mTTe) and the traditional approximate method (blue dashed line with fuzzy median mTTe’). The consistent leftward shift of the proposed algorithm’s fuzzy median demonstrates its superior precision. (G) Bar chart quantifying the relative model uncertainty reduction achieved by the proposed algorithm, which increases progressively from 1.20% to 7.49% as the system scales from 4 to 9 events, confirming the algorithm’s enhanced effectiveness in larger, more complex systems.This demonstrates a critical aspect of robustness: graceful scaling. The algorithm consistently outperforms the traditional method, and the benefit of using the precise method becomes more pronounced for larger, more complex systems. This predictable enhancement—where increased complexity leads to greater relative accuracy—provides engineers with high confidence when applying the algorithm to real-world systems of unknown or variable scale. The algorithm effectively controls uncertainty propagation, ensuring reliable risk assessment even as the system model grows in complexity. (TIF) [file pone.0335759.s014.tif]

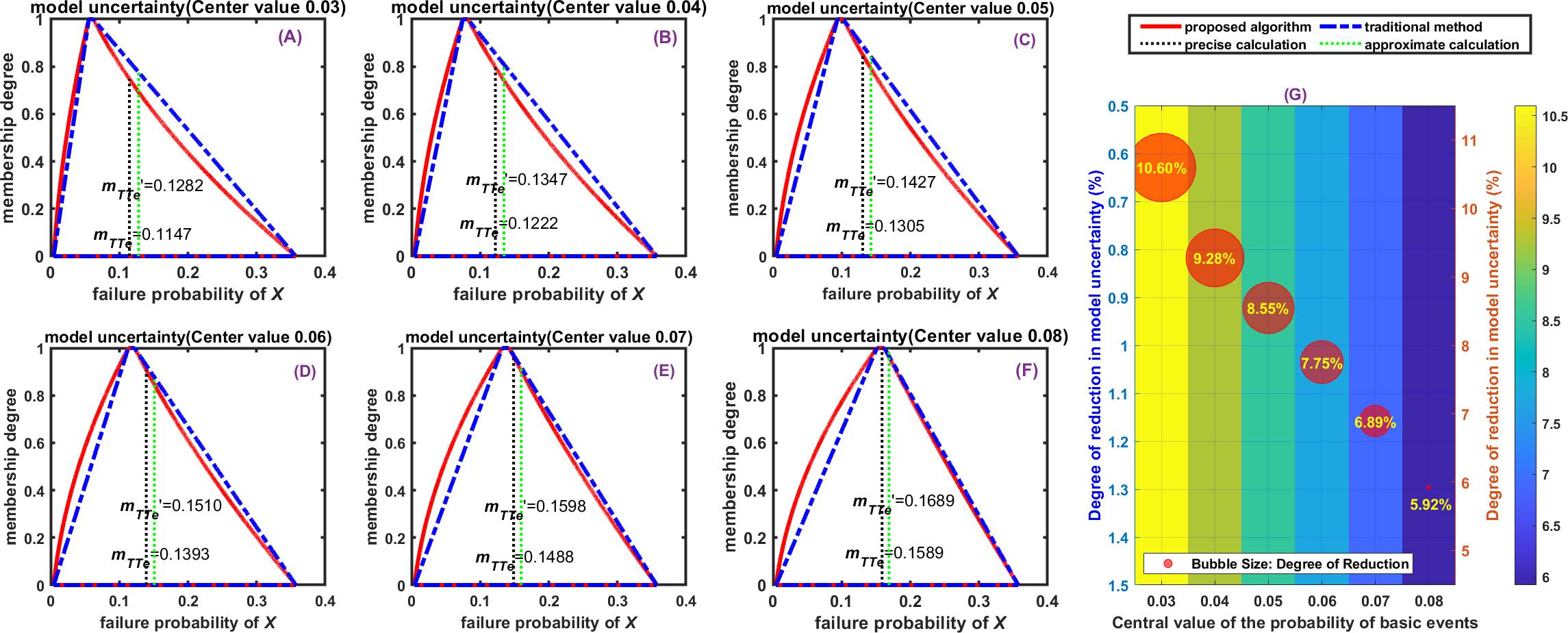

Supplement: S15 Fig — (A–F) Membership functions of the top event probability as the fuzzy median of the AND event increases from 0.03 to 0.08. Each subplot compares the proposed precise algorithm (red solid line, fuzzy median m TTe ) and the traditional method (blue dashed line, fuzzy median m TTe’ ). The proposed algorithm consistently yields a lower fuzzy median, reflecting reduced uncertainty. (G) Bubble plot showing the relative uncertainty reduction (bubble size) achieved by the proposed algorithm. As the fuzzy median of the AND event increases, the reduction in model uncertainty decreases consistently (from 10.60% to 5.92%), demonstrating the algorithm’s adaptive robustness across varying input probabilities. (TIF) [file pone.0335759.s015.tif]

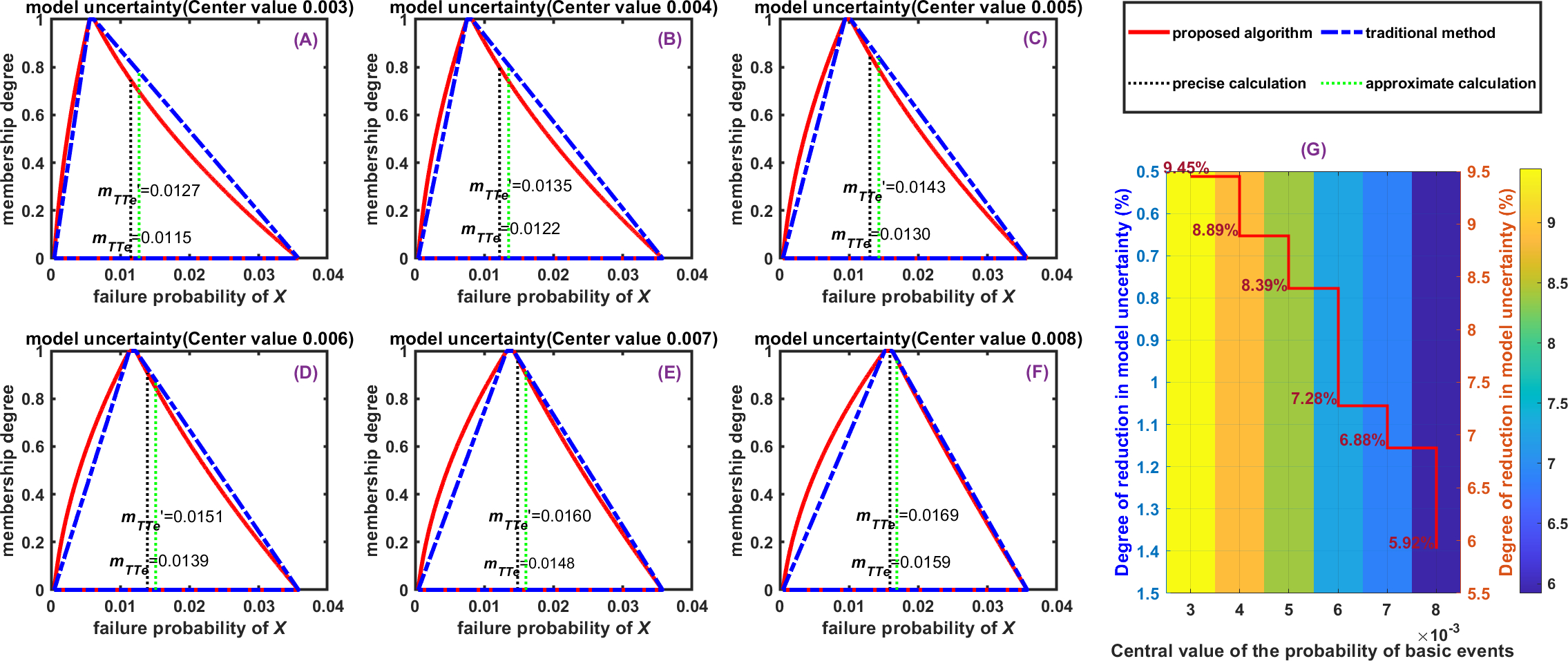

Supplement: S16 Fig — (A–F) Membership functions of the top event probability as the fuzzy median of the AND event increases from 0.003 to 0.008. Each subplot compares the proposed precise algorithm (red solid line, fuzzy median mTTe) and the traditional method (blue dashed line, fuzzy median mTTe’). The proposed algorithm consistently yields a lower fuzzy median, reflecting reduced uncertainty. (G) Step chart illustrating the relative uncertainty reduction achieved by the proposed algorithm. As the fuzzy median of the AND event increases, the reduction in model uncertainty decreases consistently (from 9.45% to 5.92%), demonstrating the algorithm’s robust performance even for low-probability events. Critically, the data reveals that the algorithm’s advantage over the traditional method is preserved regardless of the input probability’s magnitude. The difference in results between the precise and traditional methods follows a consistent pattern, and the percentage of uncertainty reduction remains significant. This demonstrates that the algorithm is not overly sensitive to the specific values of input parameters. It performs reliably whether the additional event has a high or low probability, confirming its robust stability and suitability for assessing systems with diverse risk levels. (TIF) [file pone.0335759.s016.tif]

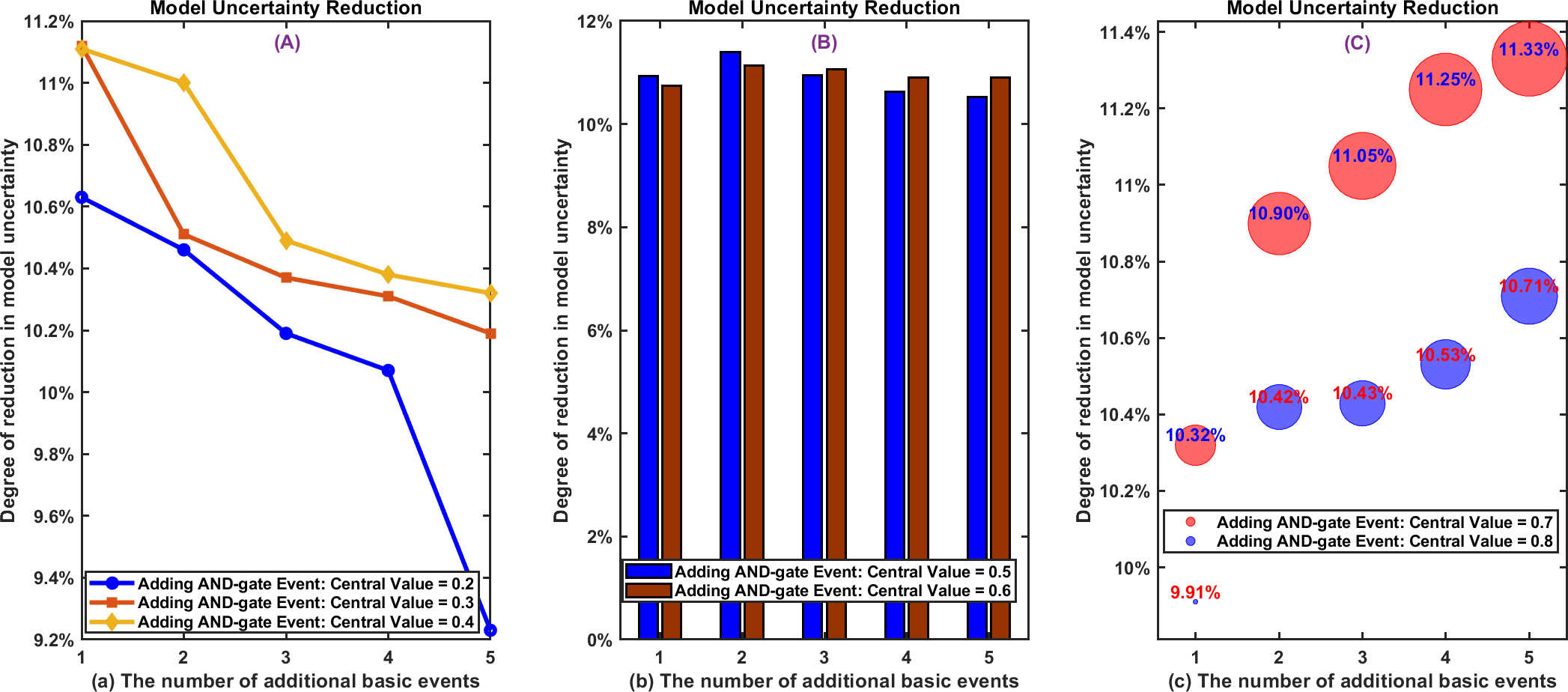

Supplement: S17 Fig — (A) Line plot showing relative uncertainty reduction as the number of AND events increases. Three distinct fuzzy median values (0.2–blue line, 0.3–red line, 0.4–yellow line) demonstrate consistent decreasing trends in uncertainty reduction with additional AND events. All lines maintain consistent ordering (yellow highest, red intermediate, blue lowest) without intersection, confirming predictable behavior across parameter variations. (B) Bar chart comparing uncertainty reduction for AND events with fuzzy medians of 0.5 (blue bars) and 0.6 (red bars). Both sequences show a characteristic pattern of initial increase followed by decrease as more AND events are added. (C) Bubble plot displaying uncertainty reduction for AND events with fuzzy medians of 0.7 (blue bubbles) and 0.8 (red bubbles). Bubble sizes increase consistently with additional AND events while maintaining rightward positional progression. Collectively, these results demonstrate the algorithm’s robust performance maintains predictable uncertainty reduction patterns across combined variations in system complexity (AND event count) and input parameters (fuzzy median values). The most significant conclusion from this comprehensive analysis is the narrow range of performance variation. As the system scales up in complexity and the input parameters vary widely, the model uncertainty reduction effect remains tightly bounded between 9.23% and 11.33%. This indicates that the algorithm’s advantage is not contingent on a specific system configuration or input value. It delivers reliable, high-quality results across a broad spectrum of conditions. (TIF) [file pone.0335759.s017.tif]
