# Supplementary material for: A novel algorithm for model uncertainty reduction in trapezoidal fuzzy fault tree risk assessment
Source: PLoS One. 2025 Dec 15;20(12):e0335759. doi: 10.1371/journal.pone.0335759 (PMC12704870; doi:10.1371/journal.pone.0335759)
Supplement: S1 Table — (PDF) [file pone.0335759.s018.pdf]

S1 Table. E4 perturbation test set (perturbation level: 5%)

Sample	a	b	c	d	Precise calculation	Approximate calculation	Reduction in uncertainty
1	0.4625	0.5946	0.7268	0.8589	0.8892	0.8691	2.32%
2	0.4540	0.5838	0.7135	0.8432	0.8870	0.8664	2.38%
3	0.4633	0.5956	0.7280	0.8603	0.8894	0.8693	2.31%
4	0.4526	0.5819	0.7112	0.8405	0.8867	0.8660	2.39%
5	0.4885	0.6280	0.7676	0.9072	0.8980	0.8773	2.36%
6	0.4513	0.5803	0.7092	0.8382	0.8863	0.8655	2.40%
7	0.4906	0.6308	0.7710	0.9112	0.8985	0.8778	2.36%
8	0.4656	0.5987	0.7317	0.8648	0.8900	0.8700	2.30%
9	0.4491	0.5774	0.7057	0.8341	0.8857	0.8648	2.42%
10	0.4630	0.5953	0.7275	0.8598	0.8894	0.8692	2.32%
11	0.4815	0.6191	0.7566	0.8942	0.8952	0.8751	2.30%
12	0.4843	0.6226	0.7610	0.8993	0.8969	0.8759	2.39%
13	0.4725	0.6075	0.7425	0.8775	0.8928	0.8722	2.37%
14	0.4792	0.6161	0.7530	0.8899	0.8946	0.8743	2.32%
15	0.4889	0.6286	0.7683	0.9080	0.8981	0.8773	2.36%
16	0.4495	0.5779	0.7063	0.8347	0.8858	0.8650	2.41%
17	0.4612	0.5929	0.7247	0.8564	0.8889	0.8687	2.33%
18	0.4491	0.5774	0.7057	0.8340	0.8857	0.8648	2.42%
19	0.4561	0.5864	0.7167	0.8470	0.8876	0.8670	2.37%
20	0.4808	0.6181	0.7555	0.8928	0.8950	0.8748	2.31%
