# Supplementary material for: A novel algorithm for model uncertainty reduction in trapezoidal fuzzy fault tree risk assessment
Source: PLoS One. 2025 Dec 15;20(12):e0335759. doi: 10.1371/journal.pone.0335759 (PMC12704870; doi:10.1371/journal.pone.0335759)
Supplement: S2 Table — (PDF) [file pone.0335759.s019.pdf]

S2 Table. E4 perturbation test set (perturbation level: 10%)

Sample	a	b	c	d	Precise calculation	Approximate calculation	Reduction in uncertainty
1	0.4913	0.6316	0.7720	0.9123	0.8987	0.8781	2.34%
2	0.5059	0.6505	0.7951	0.9396	0.9025	0.8827	2.24%
3	0.4782	0.6148	0.7514	0.8880	0.8943	0.8740	2.33%
4	0.4300	0.5529	0.6757	0.7986	0.8808	0.8818	2.56%
5	0.5102	0.6559	0.8017	0.9475	0.9036	0.8840	2.21%
6	0.4987	0.6411	0.7836	0.9261	0.9006	0.8804	2.29%
7	0.4503	0.5789	0.7076	0.8362	0.8900	0.8693	2.38%
8	0.4745	0.6101	0.7457	0.8812	0.8933	0.8729	2.36%
9	0.5160	0.6634	0.8109	0.9583	0.9051	0.8859	2.17%
10	0.4907	0.6309	0.7711	0.9113	0.8985	0.8779	2.35%
11	0.5023	0.6458	0.7893	0.9328	0.9015	0.8815	2.27%
12	0.4641	0.5967	0.7293	0.8619	0.8896	0.8696	2.30%
13	0.4676	0.6012	0.7349	0.8685	0.8915	0.8706	2.40%
14	0.4761	0.6121	0.7482	0.8842	0.8937	0.8733	2.34%
15	0.4487	0.5769	0.7051	0.8333	0.8856	0.8647	2.42%
16	0.4938	0.6349	0.7760	0.9171	0.8993	0.8789	2.33%
17	0.4708	0.6053	0.7398	0.8743	0.8924	0.8717	2.37%
18	0.4842	0.6226	0.7609	0.8993	0.8969	0.8759	2.40%
19	0.4523	0.5815	0.7108	0.8400	0.8866	0.8658	2.39%
20	0.4364	0.5611	0.6858	0.8105	0.8825	0.8608	2.51%
