# Supplementary material for: A novel algorithm for model uncertainty reduction in trapezoidal fuzzy fault tree risk assessment
Source: PLoS One. 2025 Dec 15;20(12):e0335759. doi: 10.1371/journal.pone.0335759 (PMC12704870; doi:10.1371/journal.pone.0335759)
Supplement: S3 Table — (PDF) [file pone.0335759.s020.pdf]

S3 Table. E4 perturbation test set (perturbation level: 15%)

Sample	a	b	c	d	Precise calculation	Approximate calculation	Reduction in uncertainty
**1**	0.4670	0.6004	0.7338	0.8672	0.8904	0.8704	2.29%
**2**	0.4510	0.5798	0.7087	0.8376	0.8862	0.8654	2.40%
**3**	0.5111	0.6571	0.8031	0.9491	0.9038	0.8843	2.21%
**4**	0.5100	0.6557	0.8014	0.9471	0.9035	0.8840	2.21%
**5**	0.4942	0.6354	0.7766	0.9178	0.8994	0.8791	2.32%
**6**	0.4187	0.5383	0.6579	0.7775	0.8789	0.8553	2.75%
**7**	0.4029	0.5180	0.6331	0.7482	0.8738	0.8503	2.76%
**8**	0.4788	0.6157	0.7525	0.8893	0.8945	0.8742	2.32%
**9**	0.4423	0.5687	0.6950	0.8214	0.8840	0.8627	2.46%
**10**	0.5324	0.6845	0.8366	0.9888	0.9093	0.8910	2.06%
**11**	0.5383	0.6921	0.8458	0.9996	0.9119	0.8929	2.13%
**12**	0.4403	0.5661	0.6919	0.8177	0.8835	0.8620	2.47%
**13**	0.5129	0.6594	0.8059	0.9524	0.9043	0.8848	2.20%
**14**	0.5263	0.6767	0.8270	0.9774	0.9078	0.8891	2.10%
**15**	0.4842	0.6225	0.7608	0.8992	0.8958	0.8759	2.28%
**16**	0.5246	0.6745	0.8244	0.9742	0.9073	0.8885	2.12%
**17**	0.5330	0.6853	0.8376	0.9899	0.8904	0.8704	2.29%
**18**	0.4773	0.6137	0.7501	0.8865	0.8941	0.8737	2.33%
**19**	0.5026	0.6462	0.7898	0.9335	0.9016	0.8816	2.27%
**20**	0.4812	0.6187	0.7562	0.8937	0.8951	0.8750	2.30%
