# Supplementary material for: A novel algorithm for model uncertainty reduction in trapezoidal fuzzy fault tree risk assessment
Source: PLoS One. 2025 Dec 15;20(12):e0335759. doi: 10.1371/journal.pone.0335759 (PMC12704870; doi:10.1371/journal.pone.0335759)
Supplement: S4 Table — (PDF) [file pone.0335759.s021.pdf]

S4 Table. E5 perturbation test set (perturbation level: 5%)

Sample	a	b	c	d	Precise calculation	Approximate calculation	Reduction in uncertainty
**1**	0.3354	0.4312	0.5271	0.6229	0.8665	0.8421	2.90%,
**2**	0.3502	0.4503	0.5503	0.6504	0.8701	0.8461	2.95%,
**3**	0.3573	0.4593	0.5614	0.6635	0.8723	0.8481	2.85%,
**4**	0.3528	0.4537	0.5545	0.6553	0.8715	0.8468	2.92%
**5**	0.3476	0.4469	0.5462	0.6456	0.8696	0.8454	2.86%,
**6**	0.3675	0.4725	0.5775	0.6825	0.8778	0.8537	2.83%
**7**	0.3637	0.4676	0.5715	0.6754	0.8700	0.8445	3.02%
**8**	0.3644	0.4685	0.5726	0.6767	0.8698	0.8458	2.84%,
**9**	0.3476	0.4469	0.5462	0.6456	0.8663	0.8418	2.91%,
**10**	0.3554	0.4569	0.5585	0.6600	0.8759	0.8521	2.78%,
**11**	0.3652	0.4696	0.5739	0.6783	0.8781	0.8540	2.81%
**12**	0.3674	0.4723	0.5773	0.6822	0.8784	0.8546	2.79%
**13**	0.3580	0.4603	0.5626	0.6649	0.8721	0.8478	2.86%,
**14**	0.3418	0.4394	0.5371	0.6347	0.8726	0.8485	2.84%,
**15**	0.3575	0.4597	0.5618	0.6640	0.8651	0.8399	3.01%,
**16**	0.3370	0.4333	0.5296	0.6259	0.8783	0.8544	2.79%
**17**	0.3488	0.4485	0.5481	0.6478	0.8698	0.8457	2.85%,
**18**	0.3580	0.4603	0.5625	0.6648	0.8724	0.8483	2.85%,
**19**	0.3674	0.4723	0.5773	0.6823	0.8750	0.8508	2.84%
**20**	0.3630	0.4668	0.5705	0.6742	0.8743	0.8497	2.90%
