# Supplementary material for: A novel algorithm for model uncertainty reduction in trapezoidal fuzzy fault tree risk assessment
Source: PLoS One. 2025 Dec 15;20(12):e0335759. doi: 10.1371/journal.pone.0335759 (PMC12704870; doi:10.1371/journal.pone.0335759)
Supplement: S5 Table — (PDF) [file pone.0335759.s022.pdf]

S5 Table. E5 perturbation test set (perturbation level: 10%)

Sample	a	b	c	d	Precise calculation	Approximate calculation	Reduction in uncertainty
**1**	0.3510	0.4513	0.5516	0.6519	0.8702	0.8464	2.81%,
**2**	0.3702	0.4759	0.5817	0.6875	0.8755	0.8516	2.81%,
**3**	0.3463	0.4452	0.5441	0.6431	0.8694	0.8451	2.87%,
**4**	0.3853	0.4954	0.6055	0.7156	0.8801	0.8558	2.85%
**5**	0.3865	0.4969	0.6073	0.7177	0.8803	0.8561	2.83%
**6**	0.3777	0.4857	0.5936	0.7015	0.8778	0.8537	2.83%
**7**	0.3443	0.4426	0.5410	0.6394	0.8690	0.8445	2.91%,
**8**	0.3489	0.4486	0.5483	0.6480	0.8698	0.8458	2.84%,
**9**	0.3343	0.4298	0.5253	0.6208	0.8698	0.8458	2.84%,
**10**	0.3721	0.4785	0.5848	0.6911	0.8769	0.8521	2.90%
**11**	0.3791	0.4874	0.5957	0.7040	0.8781	0.8540	2.80%
**12**	0.3812	0.4902	0.5991	0.7080	0.8784	0.8546	2.79%
**13**	0.3562	0.4580	0.5597	0.6615	0.8721	0.8478	2.86%
**14**	0.3591	0.4617	0.5642	0.6668	0.8726	0.8485	2.84%,
**15**	0.3274	0.4209	0.5144	0.6080	0.8651	0.8399	3.01%,
**16**	0.3802	0.4889	0.5975	0.7062	0.8783	0.8544	2.79%
**17**	0.3486	0.4482	0.5478	0.6474	0.8698	0.8457	2.85%,
**18**	0.3314	0.4261	0.5207	0.6154	0.8658	0.8410	2.95%,
**19**	0.3802	0.4889	0.5975	0.7062	0.8783	0.8544	2.79%
**20**	0.3706	0.4765	0.5824	0.6882	0.8756	0.8517	2.81%
