# Supplementary material for: A novel algorithm for model uncertainty reduction in trapezoidal fuzzy fault tree risk assessment
Source: PLoS One. 2025 Dec 15;20(12):e0335759. doi: 10.1371/journal.pone.0335759 (PMC12704870; doi:10.1371/journal.pone.0335759)
Supplement: S6 Table — (PDF) [file pone.0335759.s023.pdf]

S6 Table. E5 perturbation test set (perturbation level: 15%)

Sample	a	b	c	d	Precise calculation	Approximate calculation	Reduction in uncertainty
**1**	0.3925	0.5046	0.6168	0.7289	0.8814	0.8577	2.76%
**2**	0.3294	0.4235	0.5176	0.6117	0.8655	0.8405	2.97%,
**3**	0.3704	0.4762	0.5821	0.6879	0.8766	0.8516	2.92%
**4**	0.3695	0.4750	0.5806	0.6861	0.8764	0.8514	2.93%
**5**	0.3123	0.4015	0.4907	0.5799	0.8615	0.8357	3.09%
**6**	0.3423	0.4401	0.5379	0.6357	0.8687	0.8440	2.93%
**7**	0.3284	0.4222	0.5160	0.6098	0.8653	0.8402	2.99%
**8**	0.3750	0.4821	0.5893	0.6964	0.8773	0.8530	2.86%
**9**	0.3292	0.4233	0.5173	0.6114	0.8654	0.8403	2.99%,
**10**	0.3940	0.5065	0.6191	0.7316	0.8816	0.8582	2.73%
**11**	0.3866	0.4971	0.6075	0.7180	0.8804	0.8561	2.84%
**12**	0.3405	0.4378	0.5350	0.6323	0.8684	0.8434	2.96%,
**13**	0.3519	0.4524	0.5530	0.6535	0.8713	0.8466	2.92%
**14**	0.3727	0.4792	0.5856	0.6921	0.8760	0.8523	2.90%,
**15**	0.3874	0.4981	0.6088	0.7195	0.8805	0.8563	2.82%
**16**	0.3637	0.4676	0.5715	0.6754	0.8744	0.8499	2.89%
**17**	0.3600	0.4629	0.5657	0.6686	0.8728	0.8488	2.83%,
**18**	0.3337	0.4291	0.5244	0.6198	0.8662	0.8416	2.93%
**19**	0.3475	0.4468	0.5461	0.6454	0.8696	0.8454	2.86%
**20**	0.3747	0.4817	0.5888	0.6958	0.8773	0.8529	2.86%
