# Supplementary material for: A novel algorithm for model uncertainty reduction in trapezoidal fuzzy fault tree risk assessment
Source: PLoS One. 2025 Dec 15;20(12):e0335759. doi: 10.1371/journal.pone.0335759 (PMC12704870; doi:10.1371/journal.pone.0335759)
Supplement: S7 Table — (PDF) [file pone.0335759.s024.pdf]

S7 Table. E7 perturbation test set (perturbation level: 5%)

Sample	a	b	c	d	Precise calculation	Approximate calculation	Reduction in uncertainty
**1**	0.0058	0.0075	0.0091	0.0108	0.8368	0.7952	5.23%
**2**	0.0057	0.0074	0.0090	0.0106	0.8368	0.7951	5.24%
**3**	0.0053	0.0069	0.0084	0.0099	0.8367	0.7951	5.23%
**4**	0.0057	0.0073	0.0090	0.0106	0.8368	0.7951	5.24%
**5**	0.0056	0.0072	0.0087	0.0103	0.8368	0.7952	5.23%
**6**	0.0056	0.0072	0.0087	0.0103	0.8368	0.7952	5.23%
**7**	0.0054	0.0069	0.0085	0.0100	0.8367	0.7952	5.22%
**8**	0.0058	0.0074	0.0091	0.0107	0.8368	0.7952	5.23%
**9**	0.0055	0.0071	0.0086	0.0102	0.8368	0.7951	5.24%
**10**	0.0055	0.0070	0.0086	0.0101	0.8367	0.7951	5.23%
**11**	0.0055	0.0071	0.0087	0.0102	0.8368	0.7951	5.24%
**12**	0.0055	0.0071	0.0087	0.0103	0.8368	0.7951	5.24%
**13**	0.0056	0.0072	0.0088	0.0104	0.8368	0.7952	5.23%
**14**	0.0056	0.0072	0.0089	0.0105	0.8368	0.7952	5.23%
**15**	0.0055	0.0071	0.0087	0.0103	0.8368	0.7951	5.24%
**16**	0.0055	0.0071	0.0087	0.0103	0.8368	0.7951	5.24%
**17**	0.0056	0.0072	0.0088	0.0104	0.8368	0.7952	5.23%
**18**	0.0057	0.0073	0.0089	0.0106	0.8368	0.7951	5.24%
**19**	0.0059	0.0075	0.0092	0.0109	0.8368	0.7952	5.23%
**20**	0.0057	0.0074	0.0090	0.0106	0.8368	0.7951	5.24%
