# Supplementary material for: A novel algorithm for model uncertainty reduction in trapezoidal fuzzy fault tree risk assessment
Source: PLoS One. 2025 Dec 15;20(12):e0335759. doi: 10.1371/journal.pone.0335759 (PMC12704870; doi:10.1371/journal.pone.0335759)
Supplement: S8 Table — (PDF) [file pone.0335759.s025.pdf]

S8 Table. E7 perturbation test set (perturbation level: 10%)

Sample	a	b	c	d	Precise calculation	Approximate calculation	Reduction in uncertainty
1	0.0055	0.0071	0.0086	0.0102	0.8368	0.7951	5.24%
2	0.0060	0.0077	0.0094	0.0111	0.8368	0.7952	5.23%
3	0.0052	0.0067	0.0082	0.0096	0.8367	0.7950	5.25%
4	0.0051	0.0066	0.0080	0.0095	0.8367	0.7951	5.23%
5	0.0051	0.0066	0.0081	0.0095	0.8367	0.7950	5.25%
6	0.0052	0.0067	0.0082	0.0097	0.8367	0.7952	5.22%
7	0.0054	0.0069	0.0085	0.0100	0.8367	0.7952	5.22%
8	0.0054	0.0069	0.0085	0.0100	0.8367	0.7952	5.22%
9	0.0051	0.0065	0.0079	0.0094	0.8367	0.7951	5.23%
10	0.0056	0.0073	0.0089	0.0105	0.8368	0.7952	5.23%
11	0.0051	0.0066	0.0081	0.0096	0.8367	0.7951	5.23%
12	0.0052	0.0067	0.0082	0.0097	0.8367	0.7950	5.25%
13	0.0057	0.0074	0.0090	0.0107	0.8368	0.7951	5.24%
14	0.0060	0.0077	0.0094	0.0111	0.8368	0.7952	5.23%
15	0.0061	0.0079	0.0096	0.0114	0.8368	0.7953	5.22%
16	0.0057	0.0073	0.0089	0.0105	0.8368	0.7951	5.24%
17	0.0062	0.0079	0.0097	0.0114	0.8368	0.7953	5.22%
18	0.0057	0.0073	0.0089	0.0105	0.8368	0.7951	5.24%
19	0.0056	0.0072	0.0088	0.0104	0.8368	0.7952	5.23%
20	0.0054	0.0070	0.0085	0.0100	0.8367	0.7952	5.22%
