# Supplementary material for: A novel algorithm for model uncertainty reduction in trapezoidal fuzzy fault tree risk assessment
Source: PLoS One. 2025 Dec 15;20(12):e0335759. doi: 10.1371/journal.pone.0335759 (PMC12704870; doi:10.1371/journal.pone.0335759)
Supplement: S9 Table — (PDF) [file pone.0335759.s026.pdf]

S9 Table. E7 perturbation test set (perturbation level: 15%)

Sample	a	b	c	d	Precise calculation	Approximate calculation	Reduction in uncertainty
**1**	0.0055	0.0070	0.0086	0.0102	0.8367	0.7951	5.23%
**2**	0.0056	0.0072	0.0088	0.0104	0.8367	0.7951	5.23%
**3**	0.0049	0.0063	0.0077	0.0091	0.8367	0.7950	5.25%
**4**	0.0063	0.0080	0.0098	0.0116	0.8368	0.7953	5.22%
**5**	0.0049	0.0063	0.0077	0.0090	0.8367	0.7950	5.25%
**6**	0.0055	0.0071	0.0086	0.0102	0.8368	0.7951	5.24%
**7**	0.0061	0.0079	0.0097	0.0114	0.8368	0.7953	5.22%
**8**	0.0054	0.0070	0.0085	0.0101	0.8367	0.7952	5.22%
**9**	0.0058	0.0074	0.0091	0.0108	0.8368	0.7952	5.23%
**10**	0.0061	0.0079	0.0096	0.0114	0.8368	0.7953	5.22%
**11**	0.0062	0.0080	0.0098	0.0116	0.8368	0.7953	5.22%
**12**	0.0063	0.0081	0.0099	0.0117	0.8368	0.7953	5.22%
**13**	0.0051	0.0065	0.0080	0.0094	0.8367	0.7951	5.23%
**14**	0.0052	0.0067	0.0082	0.0096	0.8367	0.7950	5.25%
**15**	0.0063	0.0081	0.0099	0.0116	0.8368	0.7953	5.22%
**16**	0.0058	0.0074	0.0090	0.0107	0.8368	0.7952	5.23%
**17**	0.0056	0.0072	0.0088	0.0104	0.8368	0.7952	5.23%
**18**	0.0058	0.0074	0.0091	0.0108	0.8368	0.7952	5.23%
**19**	0.0061	0.0079	0.0096	0.0114	0.8368	0.7953	5.22%
**20**	0.0057	0.0073	0.0089	0.0105	0.8368	0.7951	5.24%
