# Supplementary material for: A novel algorithm for model uncertainty reduction in trapezoidal fuzzy fault tree risk assessment
Source: PLoS One. 2025 Dec 15;20(12):e0335759. doi: 10.1371/journal.pone.0335759 (PMC12704870; doi:10.1371/journal.pone.0335759)
Supplement: S1 Appendix — (PDF) [file pone.0335759.s027.pdf]

S1 Appendix. Monotonicity Proof for AND-Gate Systems(Left)

$$\frac{\mathrm{d}((m_{\tilde{A}_{\text{and}}})_\lambda)}{\mathrm{d}\lambda}=\frac{\mathrm{d}(\prod_{i=1}^{i=n}\left(x^{(1)}{}_i+\lambda\left(x^{(2)}{}_i-x^{(1)}{}_i\right)\right))}{\mathrm{d}\lambda}$$

$$=\left(x^{(2)}{}_1-x^{(1)}{}_1\right)\frac{\prod_{i=1}^{i=n}\left(x^{(1)}{}_i+\lambda\left(x^{(2)}{}_i-x^{(1)}{}_i\right)\right)}{x^{(1)}{}_1+\lambda(x^{(2)}{}_1-x^{(1)}{}_1)}$$

$$+\left(x^{(2)}{}_2-x^{(1)}{}_2\right)\frac{\prod_{i=1}^{i=n}\left(x^{(1)}{}_i+\lambda\left(x^{(2)}{}_i-x^{(1)}{}_i\right)\right)}{x^{(1)}{}_2+\lambda(x^{(2)}{}_2-x^{(1)}{}_2)}+\cdots$$

$$+\left(x^{(2)}{}_n-x^{(1)}{}_n\right)\frac{\prod_{i=1}^{i=n}\left(x^{(1)}{}_i+\lambda\left(x^{(2)}{}_i-x^{(1)}{}_i\right)\right)}{x^{(1)}{}_n+\lambda(x^{(2)}{}_n-x^{(1)}{}_n)}.$$

Because $\left(x^{(2)}{}_i-x^{(1)}{}_i\right)>0$, $x^{(1)}{}_i+\lambda\left(x^{(2)}{}_i-x^{(1)}{}_i\right)>0$, and $\prod_{i=1}^{i=n}\left(x^{(1)}{}_i+\lambda\left(x^{(2)}{}_i-x^{(1)}{}_i\right)\right)>0$, it follows that:

$$\left(x^{(2)}{}_1-x^{(1)}{}_1\right)\frac{\prod_{i=1}^{i=n}\left(x^{(1)}{}_i+\lambda\left(x^{(2)}{}_i-x^{(1)}{}_i\right)\right)}{x^{(1)}{}_1+\lambda(x^{(2)}{}_1-x^{(1)}{}_1)}>0.$$

$$\left(x^{(2)}{}_2-x^{(1)}{}_2\right)\frac{\prod_{i=1}^{i=n}\left(x^{(1)}{}_i+\lambda\left(x^{(2)}{}_i-x^{(1)}{}_i\right)\right)}{x^{(1)}{}_2+\lambda(x^{(2)}{}_2-x^{(1)}{}_2)}>0.$$

$$\left(x^{(2)}{}_n-x^{(1)}{}_n\right)\frac{\prod_{i=1}^{i=n}\left(x^{(1)}{}_i+\lambda\left(x^{(2)}{}_i-x^{(1)}{}_i\right)\right)}{x^{(1)}{}_n+\lambda(x^{(2)}{}_n-x^{(1)}{}_n)}>0.$$

$$\frac{\mathrm{d}((m_{\tilde{A}_{\text{and}}})_\lambda)}{\mathrm{d}\lambda}>0.$$

Therefore, $(m_{\tilde{A}_{\text{and}}})_\lambda=\prod_{i=1}^{i=n}\left(x^{(1)}{}_i+\lambda\left(x^{(2)}{}_i-x^{(1)}{}_i\right)\right)$ is a monotonically increasing function of λ .
