# Supplementary material for: A novel algorithm for model uncertainty reduction in trapezoidal fuzzy fault tree risk assessment
Source: PLoS One. 2025 Dec 15;20(12):e0335759. doi: 10.1371/journal.pone.0335759 (PMC12704870; doi:10.1371/journal.pone.0335759)
Supplement: S2 Appendix — (PDF) [file pone.0335759.s028.pdf]

S2 Appendix. Monotonicity Proof for AND-Gate Systems (Right)

$$\frac{\mathrm{d}((n_{\tilde{A}_{\mathrm{and}}})_\lambda)}{\mathrm{d}\lambda} = \frac{\mathrm{d}(\prod_{i=1}^{i=n}\left(x^{(4)}{}_i + \lambda\left(x^{(3)}{}_i - x^{(4)}{}_i\right)\right))}{\mathrm{d}\lambda}$$

$$= \left(x^{(3)}{}_1 - x^{(4)}{}_1\right)\frac{\prod_{i=1}^{i=n}\left(x^{(4)}{}_i + \lambda\left(x^{(3)}{}_i - x^{(4)}{}_i\right)\right)}{x^{(4)}{}_1 + \lambda(x^{(3)}{}_1 - x^{(4)}{}_1)}$$

$$+ \left(x^{(3)}{}_2 - x^{(4)}{}_2\right)\frac{\prod_{i=1}^{i=n}\left(x^{(4)}{}_i + \lambda\left(x^{(3)}{}_i - x^{(4)}{}_i\right)\right)}{x^{(4)}{}_2 + \lambda(x^{(3)}{}_2 - x^{(4)}{}_2)} + \cdots$$

$$+ \left(x^{(3)}{}_n - x^{(4)}{}_n\right)\frac{\prod_{i=1}^{i=n}\left(x^{(4)}{}_i + \lambda\left(x^{(3)}{}_i - x^{(4)}{}_i\right)\right)}{x^{(4)}{}_n + \lambda(x^{(3)}{}_n - x^{(4)}{}_n)}.$$

Because $\left(x^{(3)}{}_i - x^{(4)}{}_i\right) < 0$, $x^{(4)}{}_i + \lambda\left(x^{(3)}{}_i - x^{(4)}{}_i\right) > 0$, and $\prod_{i=1}^{i=n}\left(x^{(4)}{}_i + \lambda\left(x^{(3)}{}_i - x^{(4)}{}_i\right)\right) > 0$ it follows that:

$$\left(x^{(3)}{}_1 - x^{(4)}{}_1\right)\frac{\prod_{i=1}^{i=n}\left(x^{(4)}{}_i + \lambda\left(x^{(3)}{}_i - x^{(4)}{}_i\right)\right)}{x^{(4)}{}_1 + \lambda(x^{(3)}{}_1 - x^{(4)}{}_1)} < 0.$$

$$\left(x^{(3)}{}_2 - x^{(4)}{}_2\right)\frac{\prod_{i=1}^{i=n}\left(x^{(4)}{}_i + \lambda\left(x^{(3)}{}_i - x^{(4)}{}_i\right)\right)}{x^{(4)}{}_2 + \lambda(x^{(3)}{}_2 - x^{(4)}{}_2)} < 0.$$

$$\left(x^{(3)}{}_n - x^{(4)}{}_n\right)\frac{\prod_{i=1}^{i=n}\left(x^{(4)}{}_i + \lambda\left(x^{(3)}{}_i - x^{(4)}{}_i\right)\right)}{x^{(4)}{}_n + \lambda(x^{(3)}{}_n - x^{(4)}{}_n)} < 0.$$

$$\frac{\mathrm{d}((n_{\tilde{A}_{\mathrm{and}}})_\lambda)}{\mathrm{d}\lambda} < 0.$$

Thus, $(n_{\tilde{A}_{\mathrm{and}}})_\lambda = \prod_{i=1}^{i=n}\left(x^{(4)}{}_i + \lambda\left(x^{(3)}{}_i - x^{(4)}{}_i\right)\right)$ is a monotonically decreasing function of λ .
