# Supplementary material for: A novel algorithm for model uncertainty reduction in trapezoidal fuzzy fault tree risk assessment
Source: PLoS One. 2025 Dec 15;20(12):e0335759. doi: 10.1371/journal.pone.0335759 (PMC12704870; doi:10.1371/journal.pone.0335759)
Supplement: S3 Appendix — (PDF) [file pone.0335759.s029.pdf]

S3 Appendix. Monotonicity Proof for OR-Gate Systems(Left)

$$\frac{\mathrm{d}((m_{\tilde{A}_{\mathrm{or}}})_\lambda)}{\mathrm{d}\lambda} = \frac{\mathrm{d}(\prod_{i=1}^{i=n}(1 - x^{(4)}{}_i + \lambda(x^{(4)}{}_i - x^{(3)}{}_i)))}{\mathrm{d}\lambda}$$

$$= \left(x^{(4)}{}_1 - x^{(3)}{}_1\right) \frac{\prod_{i=1}^{i=n}(1 - x^{(4)}{}_i + \lambda(x^{(4)}{}_i - x^{(3)}{}_i))}{1 - x^{(4)}{}_1 + \lambda(x^{(4)}{}_1 - x^{(3)}{}_1)}$$

$$+ \left(x^{(4)}{}_2 - x^{(3)}{}_2\right) \frac{\prod_{i=1}^{i=n}(1 - x^{(4)}{}_i + \lambda(x^{(4)}{}_i - x^{(3)}{}_i))}{1 - x^{(4)}{}_2 + \lambda(x^{(4)}{}_2 - x^{(3)}{}_2)} + \cdots$$

$$+ \left(x^{(4)}{}_n - x^{(3)}{}_n\right) \frac{\prod_{i=1}^{i=n}\left(1 - x^{(4)}{}_i + \lambda\left(x^{(4)}{}_i - x^{(3)}{}_i\right)\right)}{1 - x^{(4)}{}_n + \lambda(x^{(4)}{}_n - x^{(3)}{}_n)}.$$

Since $\left(x^{(4)}{}_i - x^{(3)}{}_i\right) > 0$, $1 - x^{(4)}{}_i + \lambda(x^{(4)}{}_i - x^{(3)}{}_i) > 0$ and $\prod_{i=1}^{i=n}(1 - x^{(4)}{}_i + \lambda(x^{(4)}{}_i - x^{(3)}{}_i)) > 0$, it follows that

$$\left(x^{(4)}{}_1 - x^{(3)}{}_1\right) \frac{\prod_{i=1}^{i=n}(1 - x^{(4)}{}_i + \lambda(x^{(4)}{}_i - x^{(3)}{}_i))}{1 - x^{(4)}{}_1 + \lambda(x^{(4)}{}_1 - x^{(3)}{}_1)} > 0.$$

$$\left(x^{(4)}{}_2 - x^{(3)}{}_2\right) \frac{\prod_{i=1}^{i=n}(1 - x^{(4)}{}_i + \lambda(x^{(4)}{}_i - x^{(3)}{}_i))}{1 - x^{(4)}{}_2 + \lambda(x^{(4)}{}_2 - x^{(3)}{}_2)} > 0.$$

$$\left(x^{(4)}{}_n - x^{(3)}{}_n\right) \frac{\prod_{i=1}^{i=n}(1 - x^{(4)}{}_i + \lambda(x^{(4)}{}_i - x^{(3)}{}_i))}{1 - x^{(4)}{}_n + \lambda(x^{(4)}{}_n - x^{(3)}{}_n)} > 0.$$

$$\frac{\mathrm{d}((m_{\tilde{A}_{\mathrm{or}}})_\lambda)}{\mathrm{d}\lambda} > 0.$$

Accordingly, $(m_{\tilde{A}_{\mathrm{or}}})_\lambda = \prod_{i=1}^{i=n}(1 - x^{(4)}{}_i + \lambda(x^{(4)}{}_i - x^{(3)}{}_i))$ is a monotonically increasing function of λ .
