# Supplementary material for: A novel algorithm for model uncertainty reduction in trapezoidal fuzzy fault tree risk assessment
Source: PLoS One. 2025 Dec 15;20(12):e0335759. doi: 10.1371/journal.pone.0335759 (PMC12704870; doi:10.1371/journal.pone.0335759)
Supplement: S4 Appendix — (PDF) [file pone.0335759.s030.pdf]

S4 Appendix. Monotonicity Proof for OR-Gate Systems (Right)

$$\frac{\mathrm{d}((n_{\tilde{A}_{\mathrm{or}}})_\lambda)}{\mathrm{d}\lambda} = \frac{\mathrm{d}(\prod_{i=1}^{i=n}(1 - x^{(1)}{}_i + \lambda(x^{(1)}{}_i - x^{(2)}{}_i)))}{\mathrm{d}\lambda}$$

$$= \left(x^{(1)}{}_1 - x^{(2)}{}_1\right) \frac{\prod_{i=1}^{i=n}\left(1 - x^{(1)}{}_i + \lambda(x^{(1)}{}_i - x^{(2)}{}_i)\right)}{1 - x^{(1)}{}_1 + \lambda(x^{(1)}{}_1 - x^{(2)}{}_1)}$$

$$+ \left(x^{(1)}{}_2 - x^{(2)}{}_2\right) \frac{\prod_{i=1}^{i=n}\left(1 - x^{(1)}{}_i + \lambda(x^{(1)}{}_i - x^{(2)}{}_i)\right)}{1 - x^{(1)}{}_2 + \lambda(x^{(1)}{}_2 - x^{(2)}{}_2)} + \cdots$$

$$+ \left(x^{(1)}{}_n - x^{(2)}{}_n\right) \frac{\prod_{i=1}^{i=n}\left(1 - x^{(1)}{}_i + \lambda\left(x^{(1)}{}_i - x^{(2)}{}_i\right)\right)}{1 - x^{(1)}{}_n + \lambda(x^{(1)}{}_n - x^{(2)}{}_n)}.$$

Since $(x^{(1)}{}_i - x^{(2)}{}_i) < 0$, $1 - x^{(1)}{}_i + \lambda(x^{(1)}{}_i - x^{(2)}{}_i) > 0$ and $\prod_{i=1}^{i=n}\left(1 - x^{(1)}{}_i + \lambda(x^{(1)}{}_i - x^{(2)}{}_i)\right) > 0$,

it follows that:

$$\left(x^{(1)}{}_1 - x^{(2)}{}_1\right) \frac{\prod_{i=1}^{i=n}\left(1 - x^{(1)}{}_i + \lambda(x^{(1)}{}_i - x^{(2)}{}_i)\right)}{1 - x^{(1)}{}_1 + \lambda(x^{(1)}{}_1 - x^{(2)}{}_1)} < 0.$$

$$\left(x^{(1)}{}_2 - x^{(2)}{}_2\right) \frac{\prod_{i=1}^{i=n}\left(1 - x^{(1)}{}_i + \lambda(x^{(1)}{}_i - x^{(2)}{}_i)\right)}{1 - x^{(1)}{}_2 + \lambda(x^{(1)}{}_2 - x^{(2)}{}_2)} < 0.$$

$$\left(x^{(1)}{}_n - x^{(2)}{}_n\right) \frac{\prod_{i=1}^{i=n}\left(1 - x^{(1)}{}_i + \lambda(x^{(1)}{}_i - x^{(2)}{}_i)\right)}{1 - x^{(1)}{}_n + \lambda(x^{(1)}{}_n - x^{(2)}{}_n)} < 0.$$

$$\frac{\mathrm{d}((n_{\tilde{A}_{\mathrm{or}}})_\lambda)}{\mathrm{d}\lambda} < 0.$$

Consequently,

$(n_{\tilde{A}_{\mathrm{or}}})_\lambda = \prod_{i=1}^{i=n}\left(1 - x^{(1)}{}_i + \lambda(x^{(1)}{}_i - x^{(2)}{}_i)\right)$ is obtained as a monotonically decreasing function of λ.
