# Supplementary material for: A novel algorithm for model uncertainty reduction in trapezoidal fuzzy fault tree risk assessment
Source: PLoS One. 2025 Dec 15;20(12):e0335759. doi: 10.1371/journal.pone.0335759 (PMC12704870; doi:10.1371/journal.pone.0335759)
Supplement: S5 Appendix — (PDF) [file pone.0335759.s031.pdf]

S5 Appendix. Monotonicity Proof for AND–OR Gates (Left)

Assume the basic events in G_1 are a_1, $a_2, \cdots, a_i, \cdots, a_p$, in G_2 are b_1, $b_2, \cdots, b_j, \cdots, b_q$, and in G_k are c_1, $c_2, \cdots, c_t, \cdots, c_s$. Repeated events are allowed among G_1、G_2、$\cdots G_k$.

Proof of the Monotonic Increase of $(\boldsymbol{m}_{\tilde{A}_{\text{all}}})_{\lambda}$:

$$\frac{\mathrm{d}((m_{\tilde{A}_{\text{all}}})_{\lambda})}{\mathrm{d}\lambda} = \frac{\mathrm{d}(\sum_{k=1}^{k=n_G} \prod_{x_i \in G_k} \left(x^{(1)}{}_i + \lambda\left(x^{(2)}{}_i - x^{(1)}{}_i\right)\right))}{\mathrm{d}\lambda}$$

$$= \Bigg[\Big(x^{(2)}{}_{a_1}$$

$$- x^{(1)}{}_{a_1} \Big) \frac{\prod_{x_{a_i} \in G_1} \left(x^{(1)}{}_{a_i} + \lambda \big(x^{(2)}{}_{a_i} - x^{(1)}{}_{a_i} \big) \right)}{x^{(1)}{}_{a_1} + \lambda \big(x^{(2)}{}_{a_1} - x^{(1)}{}_{a_1} \big)}$$

$$+ \left(x^{(2)}{}_{a_2} - x^{(1)}{}_{a_2} \right) \frac{\prod_{x_{a_i} \in G_1} \left(x^{(1)}{}_{a_i} + \lambda \big(x^{(2)}{}_{a_i} - x^{(1)}{}_{a_i} \big) \right)}{x^{(1)}{}_{a_2} + \lambda \big(x^{(2)}{}_{a_2} - x^{(1)}{}_{a_2} \big)}$$

$$+ \cdots$$

$$+ \Big(x^{(2)}{}_{a_p}$$

$$- x^{(1)}{}_{a_p} \Big) \frac{\prod_{x_{a_i} \in G_1} \left(x^{(1)}{}_{a_i} + \lambda \big(x^{(2)}{}_{a_i} - x^{(1)}{}_{a_i} \big) \right)}{x^{(1)}{}_{a_p} + \lambda \left(x^{(2)}{}_{a_p} - x^{(1)}{}_{a_p} \right)} \Bigg]$$

$$+ \Bigg[\Big(x^{(2)}{}_{b_1}$$

$$- x^{(1)}{}_{b_1} \Big) \frac{\prod_{x_{b_j} \in G_2} \left(x^{(1)}{}_{b_j} + \lambda \left(x^{(2)}{}_{b_j} - x^{(1)}{}_{b_j} \right) \right)}{x^{(1)}{}_{b_1} + \lambda \big(x^{(2)}{}_{b_1} - x^{(1)}{}_{b_1} \big)}$$

$$+ \Big(x^{(2)}{}_{b_2}$$

$$- x^{(1)}{}_{b_2} \Big) \frac{\prod_{x_{b_j} \in G_2} \left(x^{(1)}{}_{b_j} + \lambda \left(x^{(2)}{}_{b_j} - x^{(1)}{}_{b_j} \right) \right)}{x^{(1)}{}_{b_2} + \lambda \big(x^{(2)}{}_{b_2} - x^{(1)}{}_{b_2} \big)} + \cdots$$

$$+ \Big(x^{(2)}{}_{b_q}$$

$$- x^{(1)}{}_{b_q} \Big) \frac{\prod_{x_{b_j} \in G_2} \left(x^{(1)}{}_{b_j} + \lambda \left(x^{(2)}{}_{b_j} - x^{(1)}{}_{b_j} \right) \right)}{x^{(1)}{}_{b_q} + \lambda \left(x^{(2)}{}_{b_q} - x^{(1)}{}_{b_q} \right)} \Bigg]$$

$$+ \cdots \Bigg[\Big(x^{(2)}{}_{c_1}$$

$$- x^{(1)}{}_{c_1} \Big) \frac{\prod_{x_{c_t} \in G_k} \left(x^{(1)}{}_{c_t} + \lambda \big(x^{(2)}{}_{c_t} - x^{(1)}{}_{c_t} \big) \right)}{x^{(1)}{}_{c_1} + \lambda \big(x^{(2)}{}_{c_1} - x^{(1)}{}_{c_1} \big)}$$

$$+ \left(x^{(2)}{}_{c_2} - x^{(1)}{}_{c_2} \right) \frac{\prod_{x_{c_t} \in G_k} \left(x^{(1)}{}_{c_t} + \lambda \big(x^{(2)}{}_{c_t} - x^{(1)}{}_{c_t} \big) \right)}{x^{(1)}{}_{c_2} + \lambda \big(x^{(2)}{}_{c_2} - x^{(1)}{}_{c_2} \big)}$$

$$+ \cdots$$

$$+\left(x^{(2)}{}_{c_s}-x^{(1)}{}_{c_s}\right)\frac{\prod_{x_{c_t}\in G_k}\left(x^{(1)}{}_{c_t}+\lambda\left(x^{(2)}{}_{c_t}-x^{(1)}{}_{c_t}\right)\right)}{x^{(1)}{}_{c_s}+\lambda\left(x^{(2)}{}_{c_s}-x^{(1)}{}_{c_s}\right)}\Bigg].$$

Given $\left(x^{(2)}{}_{a_p}-x^{(1)}{}_{a_p}\right)>0$, $x^{(1)}{}_{a_p}+\lambda\left(x^{(2)}{}_{a_p}-x^{(1)}{}_{a_p}\right)>0$, it is evident that：

$$\prod_{x_{a_i}\in G_1}\left(x^{(1)}{}_{a_i}+\lambda\left(x^{(2)}{}_{a_i}-x^{(1)}{}_{a_i}\right)\right)>0 ,$$

$$\left(x^{(2)}{}_{a_p}-x^{(1)}{}_{a_p}\right)\frac{\prod_{x_{a_i}\in G_1}\left(x^{(1)}{}_{a_i}+\lambda\left(x^{(2)}{}_{a_i}-x^{(1)}{}_{a_i}\right)\right)}{x^{(1)}{}_{a_p}+\lambda\left(x^{(2)}{}_{a_p}-x^{(1)}{}_{a_p}\right)}>0 .$$

Furthermore, considering $\left(x^{(2)}{}_{b_q}-x^{(1)}{}_{b_q}\right)>0$, $x^{(1)}{}_{b_q}+\lambda\left(x^{(2)}{}_{b_q}-x^{(1)}{}_{b_q}\right)>0$, we derive:

$$\prod_{x_{b_j}\in G_2}\left(x^{(1)}{}_{b_j}+\lambda\left(x^{(2)}{}_{b_j}-x^{(1)}{}_{b_j}\right)\right)>0.$$

$$\left(x^{(2)}{}_{b_q}-x^{(1)}{}_{b_q}\right)\frac{\prod_{x_{b_j}\in G_2}\left(x^{(1)}{}_{b_j}+\lambda\left(x^{(2)}{}_{b_j}-x^{(1)}{}_{b_j}\right)\right)}{x^{(1)}{}_{b_q}+\lambda\left(x^{(2)}{}_{b_q}-x^{(1)}{}_{b_q}\right)}>0.$$

Lastly, from $\left(x^{(2)}{}_{c_s}-x^{(1)}{}_{c_s}\right)>0$, $x^{(1)}{}_{c_s}+\lambda\left(x^{(2)}{}_{c_s}-x^{(1)}{}_{c_s}\right)>0$, it follows that：

$$\prod_{x_{c_t}\in G_k}\left(x^{(1)}{}_{c_t}+\lambda\left(x^{(2)}{}_{c_t}-x^{(1)}{}_{c_t}\right)\right)>0,$$

$$\left(x^{(2)}{}_{c_s}-x^{(1)}{}_{c_s}\right)\frac{\prod_{x_{c_t}\in G_k}\left(x^{(1)}{}_{c_t}+\lambda\left(x^{(2)}{}_{c_t}-x^{(1)}{}_{c_t}\right)\right)}{x^{(1)}{}_{c_s}+\lambda\left(x^{(2)}{}_{c_s}-x^{(1)}{}_{c_s}\right)}>0.$$

Combining these results, we conclude that

$$\frac{\mathrm{d}((m_{\tilde{A}_{\mathrm{all}}})_\lambda)}{\mathrm{d}\lambda}>0.$$

Which confirms that $(m_{\tilde{A}_{\mathrm{all}}})_\lambda$ exhibits a monotonically increasing relationship with λ .
