# Supplementary material for: A novel algorithm for model uncertainty reduction in trapezoidal fuzzy fault tree risk assessment
Source: PLoS One. 2025 Dec 15;20(12):e0335759. doi: 10.1371/journal.pone.0335759 (PMC12704870; doi:10.1371/journal.pone.0335759)
Supplement: S6 Appendix — (PDF) [file pone.0335759.s032.pdf]

S6 Appendix. Monotonicity Proof for AND–OR Gates (Right)

Assume the basic events in G_1 are a_1, $a_2, \cdots, a_i, \cdots, a_p$, in G_2 are b_1, $b_2, \cdots, b_j, \cdots, b_q$, and in G_k are c_1, $c_2, \cdots, c_t, \cdots, c_s$. Repeated events are allowed among G_1、G_2、$\cdots G_k$.

The proof of monotonicity for $(\boldsymbol{n}_{\tilde{A}_{\mathrm{all}}})_{\boldsymbol{\lambda}}$

$$\frac{\mathrm{d}((n_{\tilde{A}_{\mathrm{all}}})_{\lambda})}{\mathrm{d}\lambda}=\frac{\mathrm{d}(\sum_{k=1}^{k=n_G}\prod_{x_i\in G_k}(x^{(4)}{}_i+\lambda(x^{(3)}{}_i-x^{(4)}{}_i)))}{\mathrm{d}\lambda}$$

$$=\Bigg[\Big(x^{(3)}{}_{a_1}$$

$$-x^{(4)}{}_{a_1}\Big)\frac{\prod_{x_{a_i}\in G_1}\left(x^{(4)}{}_{a_i}+\lambda\left(x^{(3)}{}_{a_i}-x^{(4)}{}_{a_i}\right)\right)}{x^{(4)}{}_{a_1}+\lambda\left(x^{(3)}{}_{a_1}-x^{(4)}{}_{a_1}\right)}$$

$$+\left(x^{(3)}{}_{a_2}-x^{(4)}{}_{a_2}\right)\frac{\prod_{x_{a_i}\in G_1}\left(x^{(4)}{}_{a_i}+\lambda\left(x^{(3)}{}_{a_i}-x^{(4)}{}_{a_i}\right)\right)}{x^{(4)}{}_{a_2}+\lambda\left(x^{(3)}{}_{a_2}-x^{(4)}{}_{a_2}\right)}$$

$$+\cdots$$

$$+\Big(x^{(3)}{}_{a_p}$$

$$-x^{(4)}{}_{a_p}\Big)\frac{\prod_{x_{a_i}\in G_1}\left(x^{(4)}{}_{a_i}+\lambda\left(x^{(3)}{}_{a_i}-x^{(4)}{}_{a_i}\right)\right)}{x^{(4)}{}_{a_p}+\lambda\left(x^{(3)}{}_{a_p}-x^{(4)}{}_{a_p}\right)}\Bigg]$$

$$+\Bigg[\Big(x^{(3)}{}_{b_1}$$

$$-x^{(4)}{}_{b_1}\Big)\frac{\prod_{x_{b_j}\in G_2}\left(x^{(4)}{}_{b_j}+\lambda\left(x^{(3)}{}_{b_j}-x^{(4)}{}_{b_j}\right)\right)}{x^{(4)}{}_{b_1}+\lambda\left(x^{(3)}{}_{b_1}-x^{(4)}{}_{b_1}\right)}$$

$$+\Big(x^{(3)}{}_{b_2}$$

$$-x^{(4)}{}_{b_2}\Big)\frac{\prod_{x_{b_j}\in G_2}\left(x^{(4)}{}_{b_j}+\lambda\left(x^{(3)}{}_{b_j}-x^{(4)}{}_{b_j}\right)\right)}{x^{(4)}{}_{b_2}+\lambda\left(x^{(3)}{}_{b_2}-x^{(4)}{}_{b_2}\right)}+\cdots$$

$$+\Big(x^{(3)}{}_{b_q}$$

$$-x^{(4)}{}_{b_q}\Big)\frac{\prod_{x_{b_j}\in G_2}\left(x^{(4)}{}_{b_j}+\lambda\left(x^{(3)}{}_{b_j}-x^{(4)}{}_{b_j}\right)\right)}{x^{(4)}{}_{b_q}+\lambda\left(x^{(3)}{}_{b_q}-x^{(4)}{}_{b_q}\right)}\Bigg]$$

$$+\cdots\Bigg[\Big(x^{(3)}{}_{c_1}$$

$$-x^{(4)}{}_{c_1}\Big)\frac{\prod_{x_{c_t}\in G_k}\left(x^{(4)}{}_{c_t}+\lambda\left(x^{(3)}{}_{c_t}-x^{(4)}{}_{c_t}\right)\right)}{x^{(4)}{}_{c_1}+\lambda\left(x^{(3)}{}_{c_1}-x^{(4)}{}_{c_1}\right)}$$

$$+\left(x^{(3)}{}_{c_2}-x^{(4)}{}_{c_2}\right)\frac{\prod_{x_{c_t}\in G_k}\left(x^{(4)}{}_{c_t}+\lambda\left(x^{(3)}{}_{c_t}-x^{(4)}{}_{c_t}\right)\right)}{x^{(4)}{}_{c_2}+\lambda\left(x^{(3)}{}_{c_2}-x^{(4)}{}_{c_2}\right)}$$

$$+\cdots$$

$$+\left(x^{(3)}{}_{c_s}-x^{(4)}{}_{c_s}\right)\frac{\prod_{x_{c_t}\in G_k}\left(x^{(4)}{}_{c_t}+\lambda\left(x^{(3)}{}_{c_t}-x^{(4)}{}_{c_t}\right)\right)}{x^{(4)}{}_{c_s}+\lambda\left(x^{(3)}{}_{c_s}-x^{(4)}{}_{c_s}\right)}\Bigg].$$

Given $\left(x^{(3)}{}_{a_p}-x^{(4)}{}_{a_p}\right)<0$ and $x^{(4)}{}_{a_p}+\lambda\left(x^{(3)}{}_{a_p}-x^{(4)}{}_{a_p}\right)>0$, it is clear that：

$$\prod_{x_{a_i}\in G_1}\left(x^{(4)}{}_{a_i}+\lambda\left(x^{(3)}{}_{a_i}-x^{(4)}{}_{a_i}\right)\right)>0,$$

$$\left(x^{(3)}{}_{a_p}-x^{(4)}{}_{a_p}\right)\frac{\prod_{x_{a_i}\in G_1}\left(x^{(4)}{}_{a_i}+\lambda\left(x^{(3)}{}_{a_i}-x^{(4)}{}_{a_i}\right)\right)}{x^{(4)}{}_{a_p}+\lambda\left(x^{(3)}{}_{a_p}-x^{(4)}{}_{a_p}\right)}<0.$$

Additionally, based on $\left(x^{(3)}{}_{b_q}-x^{(4)}{}_{b_q}\right)<0$ and $x^{(4)}{}_{b_q}+\lambda\left(x^{(3)}{}_{b_q}-x^{(4)}{}_{b_q}\right)>0$, we obtain:

$$\prod_{x_{b_j}\in G_2}\left(x^{(4)}{}_{b_j}+\lambda\left(x^{(3)}{}_{b_j}-x^{(4)}{}_{b_j}\right)\right)>0,$$

$$\left(x^{(3)}{}_{b_q}-x^{(4)}{}_{b_q}\right)\frac{\prod_{x_{b_j}\in G_2}\left(x^{(4)}{}_{b_j}+\lambda\left(x^{(3)}{}_{b_j}-x^{(4)}{}_{b_j}\right)\right)}{x^{(4)}{}_{b_q}+\lambda\left(x^{(3)}{}_{b_q}-x^{(4)}{}_{b_q}\right)}<0.$$

Finally, from $\left(x^{(3)}{}_{c_s}-x^{(4)}{}_{c_s}\right)<0$ and $x^{(4)}{}_{c_s}+\lambda\left(x^{(3)}{}_{c_s}-x^{(4)}{}_{c_s}\right)>0$, we establish

$$\prod_{x_{c_t}\in G_k}\left(x^{(4)}{}_{c_t}+\lambda\left(x^{(3)}{}_{c_t}-x^{(4)}{}_{c_t}\right)\right)>0,$$

$$\left(x^{(3)}{}_{c_s}-x^{(4)}{}_{c_s}\right)\frac{\prod_{x_{c_t}\in G_k}\left(x^{(4)}{}_{c_t}+\lambda\left(x^{(3)}{}_{c_t}-x^{(4)}{}_{c_t}\right)\right)}{x^{(4)}{}_{c_s}+\lambda\left(x^{(3)}{}_{c_s}-x^{(4)}{}_{c_s}\right)}<0.$$

Synthesizing these findings, we conclude that:

$$\frac{\mathrm{d}\left((n_{\tilde{A}_{\mathrm{all}}})_{\lambda}\right)}{\mathrm{d}\lambda}<0.$$
